# Inferring active regulatory networks from gene expression data using a combination of prior knowledge and enrichment analysis

**DOI:** 10.1186/s12859-016-1040-7

**Published:** 2016-06-06

**Authors:** Panagiotis Chouvardas, George Kollias, Christoforos Nikolaou

**Affiliations:** Biomedical Sciences Research Center “Alexander Fleming”, Vari, 16672 Greece; Department of Physiology, Medical School, University of Athens, Athens, 11527 Greece; Computational Genomics Group, Department of Biology, University of Crete, Voutes Campus, Heraklion, 70013 Greece; Division of Immunology, Biomedical Sciences Research Center “Alexander Fleming”, Vari, 16672 Greece; Department of Physiology, School of Medicine, National and Kapodistrian University of Athens, Athens, 11527 Greece

**Keywords:** Transcriptional regulation, Gene regulatory network, Gene set enrichment analysis

## Abstract

**Background:**

Under both physiological and pathological conditions gene expression programs are shaped through the interplay of regulatory proteins and their gene targets, interactions between which form intricate gene regulatory networks (GRN). While the assessment of genome-wide expression for the complete set of genes at a given condition has become rather straight-forward and is performed routinely, we are still far from being able to infer the topology of gene regulation simply by analyzing its “descendant” expression profile. In this work we are trying to overcome the existing limitations for the inference and study of such regulatory networks. We are combining our approach with state-of-the-art gene set enrichment analyses in order to create a tool, called Regulatory Network Enrichment Analysis (RNEA) that will prioritize regulatory and functional characteristics of a genome-wide expression experiment.

**Results:**

RNEA combines prior knowledge, originating from manual literature curation and small-scale experimental data, to construct a reference network of interactions and then uses enrichment analysis coupled with a two-level hierarchical parsing of the network, to infer the most relevant subnetwork for a given experimental setting. It is implemented as an R package, currently supporting human and mouse datasets and was herein tested on one test case for each of the two organisms. In both cases, RNEA’s gene set enrichment analysis was comparable to state-of-the-art methodologies. Moreover, through its distinguishing feature of regulatory subnetwork reconstruction, RNEA was able to define the key transcriptional regulators for the studied systems as supported from the literature.

**Conclusions:**

RNEA constitutes a novel computational approach to obtain regulatory interactions directly from a genome-wide expression profile. Its simple implementation, with minimal requirements from the user is coupled with easy-to-parse enrichment lists and a subnetwork file that may be readily visualized to reveal the most important components of the regulatory hierarchy. The combination of prior information and novel concept of a hierarchical reconstruction of regulatory interactions makes RNEA a very useful tool for a first-level interpretation of gene expression profiles.

**Electronic supplementary material:**

The online version of this article (doi:10.1186/s12859-016-1040-7) contains supplementary material, which is available to authorized users.

## Background

The advent of high-throughput genomics that started with DNA microarrays and is now rapidly shifting to next-generation-sequencing, has been producing a vast amount of information regarding a variety of cellular functions. In the context of gene expression measurements, genome-wide profiling approaches through RNASeq have made possible the monitoring of gene expression at unprecedented resolution, allowing not only the detection of genes present in the cell in only a few mRNA copies, but also revealing the transcriptional complexity reflected in the use of alternative transcript isoforms [1–3]. In this sense, the output of all genome-wide expression profiling approaches, summarized in lists of differentially expressed genes, may be seen as an accurate reflection of the intricate regulatory dynamics that reshape the expression programs of a cell even, under the most subtle perturbations. Such differentially expressed (DE) gene lists are often quite extended, including a great number of genes, for which there is little if any knowledge related to the system under study. In this regard, considerable effort has been directed towards methods for the efficient analysis and interpretation of whole transcriptome read-outs [4–6]. Such analyses focus mostly on the testing of DE genes for enrichment in various functional groupings, such as Gene Ontology (GO) terms [7], or molecular pathways such as those compiled by the Kyoto Encyclopedia of Genes and Genomes (KEGG) [8]. Biologists have thus to choose from a variety of existing tools for data analysis and interpretation.

Over the years, the accumulation of genome-wide data has increased the possible gene groupings and categorizations, for which enrichment analyses may be conducted. These may now include protein families, molecular signatures defined under certain pathological conditions, chromosomal territories or co-expressed gene clusters obtained through meta-analyses of publicly available datasets [9, 10]. Among the various available gene categorizations, those referring to gene regulation are of particular interest, not only because of their wealth, encompassing predicted and experimental transcription factor binding sites and miRNA targets, but mainly because of their potential in inferring the gene regulatory program responsible for the observed expression profile.

Gene regulation takes place in various stages among which, transcription is the one most readily analyzed and easy to quantify. Given a certain stimulus or under specific conditions, the relative abundance of a great number of mRNA species may vary due to both orchestrated changes resulting from the activation of a particular gene expression program and random noise. The main goal of a functional analysis at the regulatory level will thus be to distinguish between the two and, moreover, to propose a hierarchy for the gene regulators involved in the system under examination. The concept of hierarchical regulatory interactions between genes is not new. Master regulators are important drivers of gene expression [11] and defining them is of primary interest at both experimental [12] and theoretical levels [13, 14].

In spite of the increase of available information, the problem of determining the hierarchy of transcriptional regulators involved in given conditions remains an open question. The definition of regulatory networks of interaction is a complex difficult task that may only be achieved through the integration of multiple datasets from various sources (TF binding, miRNA expression, gene expression etc.). Currently, the reconstruction of such global networks has been limited in the context of large genomic consortia (e.g. the ENCODE Project Consortium) [15, 16], or small unicellular organisms [17, 18], but even in these cases the resulting networks are extensive and difficult to interpret. At the same time, gene expression profiles are rapidly accumulating, exploring a vast amount of possible regulatory patterns and pressing for more efficient analysis. In this sense, it becomes plausible to seek ways to predict the regulatory network using only gene expression data, in attempts to treat whole genome expression profiles as a detailed reflection of the underlying regulatory program.

Accumulating genome-wide data, coupled with detailed studies has led to the creation of large compendia of well-defined regulatory interactions for a number of model species, compendia that are now being compiled in specialized databases. The use, however, of the reported resources requires filtering of noisy or trivial information. HTRIdb [14] contains a large number of interactions (>50000), the largest part of which are inferred from ChIP experiments that are known to be extremely noisy. ORegAnno [19] contains a more moderate number of regulatory interactions but in many cases these are reported as based on “unknown evidence” or refer to unknown genes. Smaller databases such as TRED [20], or TFactS [15], on the other hand, are built through a more thorough process that involves manual curation of literature and public datasets. Finally, there are databases employing intermediate approaches such as TRRUST [16], which makes use of a text-mining algorithm coupled with manual curation of the results to populate a database of ~8000 interactions.

**Fig. 1:**
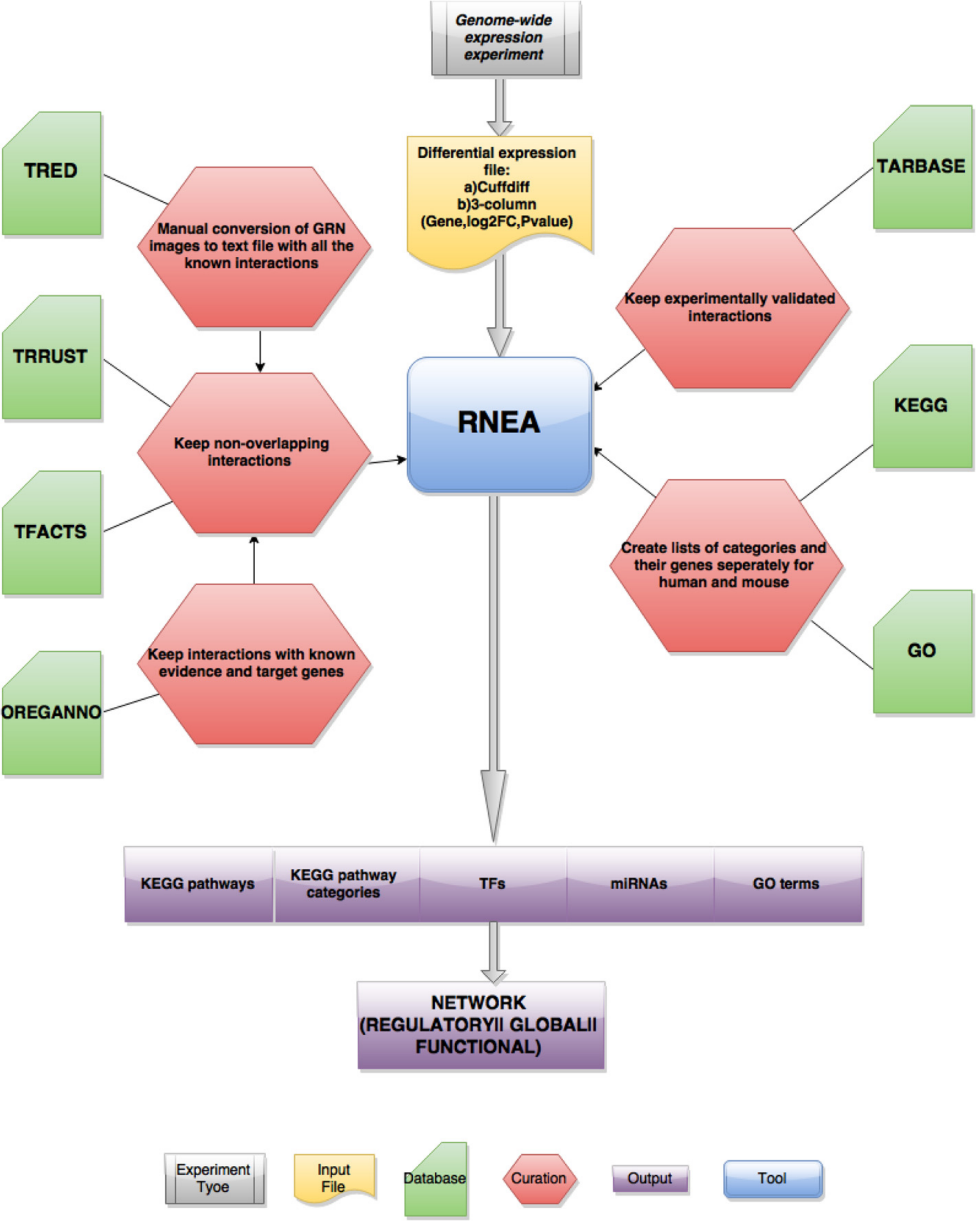
RNEA detailed workflow. RNEA, given a differential expression file (yellow node) of a genome-wide expression experiment (gray node) and based on highly confident prior knowledge (light green nodes) and manual curation (red nodes), extracts lists (purple nodes) of regulators and functional categories whose targets and members respectively are overrepresented among deregulated genes. Additionally, RNEA extracts the regulatory (or functional or global) network “activated” in the specific experiment

In this work we propose an enrichment analysis tool that uses high-quality, curated, prior knowledge on regulatory interactions to infer the hierarchy of gene regulation from a gene expression profile. The main goal is to draw significant information and prioritize important regulators and functional categories from a genome-wide expression experiment. This is done through a combination of a) manually curated prior knowledge, b) a novel approach for the inference of regulatory networks that takes into account their assumed hierarchical organization. We compiled interactions for the human and mouse regulomes from four different databases through a semi-automatic curation process in order to construct two reference networks. We then employ a novel algorithm that reconstructs a relevant regulatory subnetwork based on a combination of enriched regulators and gene target deregulation, given a genome-wide expression profile. In addition, our method, reports enriched transcriptional regulators, miRNAs, KEGG pathways and GO terms in a manner similar to standard over-representation analysis tools such as DAVID [17], or Enrichr [9]. Both processes are integrated in an over-representation analysis tool, called RNEA (Regulatory Network Enrichment Analysis), which provides highly informative outputs for the understanding of the biological system studied (Fig. 1).

We use the proposed methodology to infer regulatory networks for two test cases, one for each organism for which we have compiled information (human, mouse). We show RNEA able to reconstruct networks that are in agreement with existing knowledge of the systems under study, while at the same time they provide lists of additional candidate genes being involved in key processes. Our implementation shows RNEA to be a very useful resource for a first-level analysis of gene expression datasets in order to gain insight in the system of study and to obtain leads for genes and proteins of primary importance.

## Methods

### Pipeline

The Regulatory Network Enrichment Analysis tool (RNEA) is based on a collection of regulatory interactions compiled from manually curated databases. RNEA uses prior knowledge, coupled with standard statistical methods for the inference of active regulators, miRNAs and functional categories. Most importantly, RNEA extracts the presumably active regulatory subnetwork from a global gene regulatory network (GRN) based on the calculated transcriptional regulator - gene target enrichments, showing how significantly enriched regulators interact with their target genes and between each other. RNEA receives a gene expression profile, in the form of a complete list of differential expression values as input. This should include gene name identifiers coupled with differential expression values and significance p-values. Based on this list of differential expression, fold-change values and their accompanying *p*-values, it calculates enrichments for particular gene groupings. The output of RNEA is dual; on one hand it produces lists of over-represented gene categories in the form of current state-of-the-art approaches, but most importantly, it provides the user with a regulatory subnetwork file where the relevant gene interactions are registered. The lists can help identify important regulators and functions, while the created regulatory subnetwork, provides a view of the transcriptional regulation at a system level and may enhance the interpretation of a genome-wide expression experiment.

RNEA has been developed in R, aiming to be a cross-platform and easy-to-use tool. It is compatible with widely used differential expression analysis software such as Cufflinks [2], EdgeR [18] and DESeq [21] and may therefore be easily incorporated in already existing pipelines. Its results are displayed as HTML files with sortable tables, which include the corrected p-values for the functional and the regulatory groups respectively for greater ease of use. The regulatory subnetwork is extracted in a tab separated file format in order to be compatible with typical network visualization software, such as Cytoscape [22]. The source code, alongside the reference networks for human and mouse and detailed documentation may be found at: https://sites.google.com/a/fleming.gr/rnea/.

### Resources

In order to create a highly-confident dataset of transcriptional regulator–gene target interactions we searched for databases, which mainly contain experimentally validated or manually curated interactions. With these criteria, TRED [20], TFactS [15], Oreganno [19] and TRRUST [16] were chosen and lists of human and mouse regulator–gene-target interactions were created separately. TRED is a database designed as a resource for gene regulation. It has gathered data for many elements of regulation, such as promoters, many of which are annotated with computational tools produced by the same group. Their analysis is done genome-wide for human, mouse and rat. In this way, TRED combines promoter annotation with experimental results to assign target genes to transcriptional regulators, assignments, further refined through manual curation of the results and validation from the literature. TRED was organized in modular gene regulatory networks (GRNs) that were created and uploaded in the form of network figures (https://cb.utdallas.edu/TRED/GRN/grn.htm). These GRNs include visual representation of 34 transcription factor (TF) families. All images for human and mouse were downloaded and, in a thorough and time-consuming procedure, each interaction was recorded in a tab-separated text file.

Most of the interactions TFactS includes, overlap the dataset compiled by TRED. Most of the non-overlapping interactions are based on manual curation of literature articles, missing from the TRED reference database. We only kept species-specific interactions because in spite of the extended conservation between human and mouse at gene level, one cannot rule out significant differences existing at the level of protein-protein and protein-DNA interaction hierarchies between the two species. This was observed in the case of TRED, where in many cases the GRNs in human and mouse significantly differed for the same TF family.

**Fig. 2:**
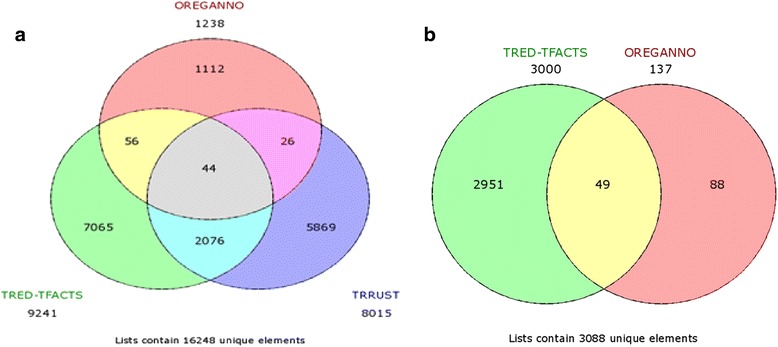
Overlap between recorded regulator-gene target interactions that were compiled for the purposes of our analyses for (**a**) the human and (**b**) the mouse reference network

TRRUST is a large database with literature-curated regulatory interactions. The authors, combining text mining in around 20 million abstracts and manual curation of the results, identified ~8000 interactions between ~750 TF and ~2000 target genes. The only limitation of this highly informative database is that it only contains data on the human regulome. ORegAnno, on the other hand, includes TF-target genes interactions for many species. Annotation is collected from users worldwide, which brings about an inherent variability in the confidence with which each interaction may be reported. The representation of interactions from different resources in our reference network is indicative of the fragmentary nature of the data, available in various databases. For the human reference network less than 0.5 % of interactions were shared between all databases (the same percentage for mouse was 1.5 %). TRRUST shared 14 % of interactions with the precompiled TRED-TFACTS human interaction set, while Oreganno had no more than a 10 % overlap with either TRRUST or TRED-TFACTS. In the case of the mouse reference network, for which no data were available in TRRUST, 95 % of the interactions originated from TRED and TFactS. A graphical representation of the partitioning of interactions in our reference networks may be seen in Fig. 2.

**Fig. 3:**
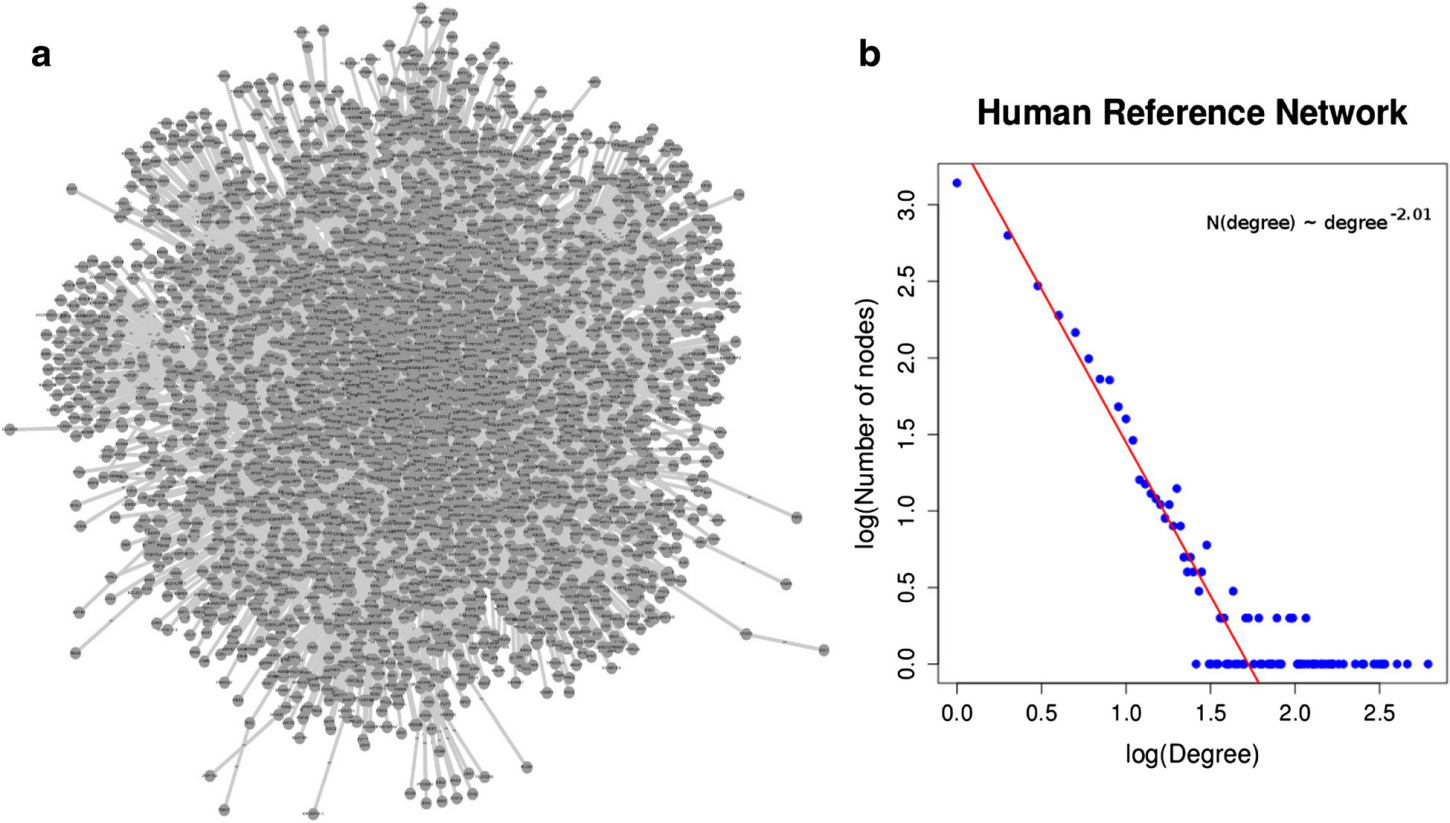
**a** A graph of a subset of the Human Reference Network containing regulator and gene-target interactions as compiled by TRED and TFactS only. **b** Node-degree rank distribution of this network shows an extensive linear relationship on a double logarithmic scale which is a hallmark of “small world”, scale-free networks. The shape and exponent of this plot do not change significantly when the complete reference network is taken into account

For all the above reasons, the interactions that had unknown evidence or unknown target genes were excluded from our collection, aiming to keep the most reliable portion of the contained information. Aiming at creating a compendium of as reliable as possible regulator-gene target interactions, we only considered interactions supported by manual literature curation and small-scale experimental validation and disregarded the ones solely based on computational approaches (e.g. automated text-mining) or originating from high-throughput experiments. Compilation of these interactions, led to the creation of two separate flat files comprising the total number of regulatory interactions for human and mouse respectively. We were thus able to create an overall “reference” regulatory network for each of the two species. The human regulatory network contained 5154 nodes and 16351 interactions while the one corresponding to mouse constituted of 1515 nodes and 3096 interactions (being significantly smaller than the human one due to the fact that TRRUST contained no mouse interaction data). Analysis of the constructed networks showed them to fit well with the assumed scale-free organization of regulatory networks in agreement with theoretical predictions and experimental data [23, 24]. The human reference distribution of node-degree values follows a power-law with an exponent close to −2 (see Fig. 3). At the same time, each transcriptional regulator was coupled with the list of its gene targets found in the network. These gene sets formed RNEA’s regulatory grouping, used in the enrichment analysis step in order to prioritize transcriptional regulators in a given experiment.

Interactions between miRNA and protein coding genes were also compiled in a way that focused on experimentally validated datasets. Data were retrieved from Tarbase [25], currently the biggest repository containing experimentally validated miRNA-gene interactions. Tarbase also includes various types of information for each interaction, and computational predictions of microT [26], a computational approach for miRNA target prediction that is based on a combination of experimental data and sequence conservation. The extraction of interactions from Tarbase was performed for human and mouse in a way that excluded all interactions which were not experimentally validated, which resulted in two lists containing 1573 and 407 interactions respectively.

RNEA also provides standard enrichment tables for Gene Ontology and Pathway annotations, the latest versions of which were downloaded from the corresponding web resources. Data for GO were obtained from the Gene Ontology Consortium (http://geneontology.org/) and Biological Pathway annotations were retrieved from the Kyoto Encyclopedia of Genes and Genomes (KEGG) (http://www.genome.jp/kegg/).

### Implementation

RNEA performs analysis at two levels. After performing a typical enrichment analysis at the levels of GO, KEGG Pathways, miRNA and protein transcriptional regulators (TF), it couples the latter with a search in the corresponding reference regulatory network, in order to extract a subset of interactions and to reconstruct the most informative regulatory subnetwork.

At a first level, RNEA employs a standard over-representation analysis in order to calculate the enrichment of deregulated genes among certain categories. In this way, it requires defined fold-change and p-value thresholds, which may be provided by the user. As this is a typical example of a statistical experiment of drawing an object from a finite population which has two distinct states without replacement, the hypergeometric test is used to calculate the significance of the enrichment. RNEA differs from most tools in the sense that it automatically performs three different types of enrichments, aiming to find regulatory (TF, miRNA) or functional (KEGG pathways, GO terms) components whose members are over-represented among a) overall DE genes, b) strictly over-expressed or c) strictly under-expressed genes. It thus also processes information separately for genes whose expression is increased or decreased. Reporting is performed through ranked lists of enrichment, after conducting a suitable FDR correction for multiple testing. The output is organized in a set of prioritized lists of regulators and functional groups.

At a second level, RNEA builds on the list of differentially expressed transcriptional regulators to create a network of regulatory interactions. It does so by tracing a subnetwork on the reference network, using a bottom-up approach that aims to reconstruct a relevant hierarchy of regulation. This includes the following steps:

1. A profile is created for each transcriptional regulator containing all of its target genes. These first-level regulators are called “parent” regulators.
2. If any of the target genes is also a regulator, it is linked to both its “parent” regulator and its “daughter” targets, thus creating a series of second-level interactions.
3. This two level profile is then used in order to extract nodes and interactions from the reference network according to three simple rules:

- o A regulator is included in the subnetwork if it is differentially expressed.
- o First-level targets of the regulator are included in the subnetwork if they are differentially expressed.
- o First-level targets of the regulator are included in the subnetwork even if they are NOT differentially expressed as long as their “parent” regulator and a second-level “daughter” target are both differentially expressed

**Fig. 4:**
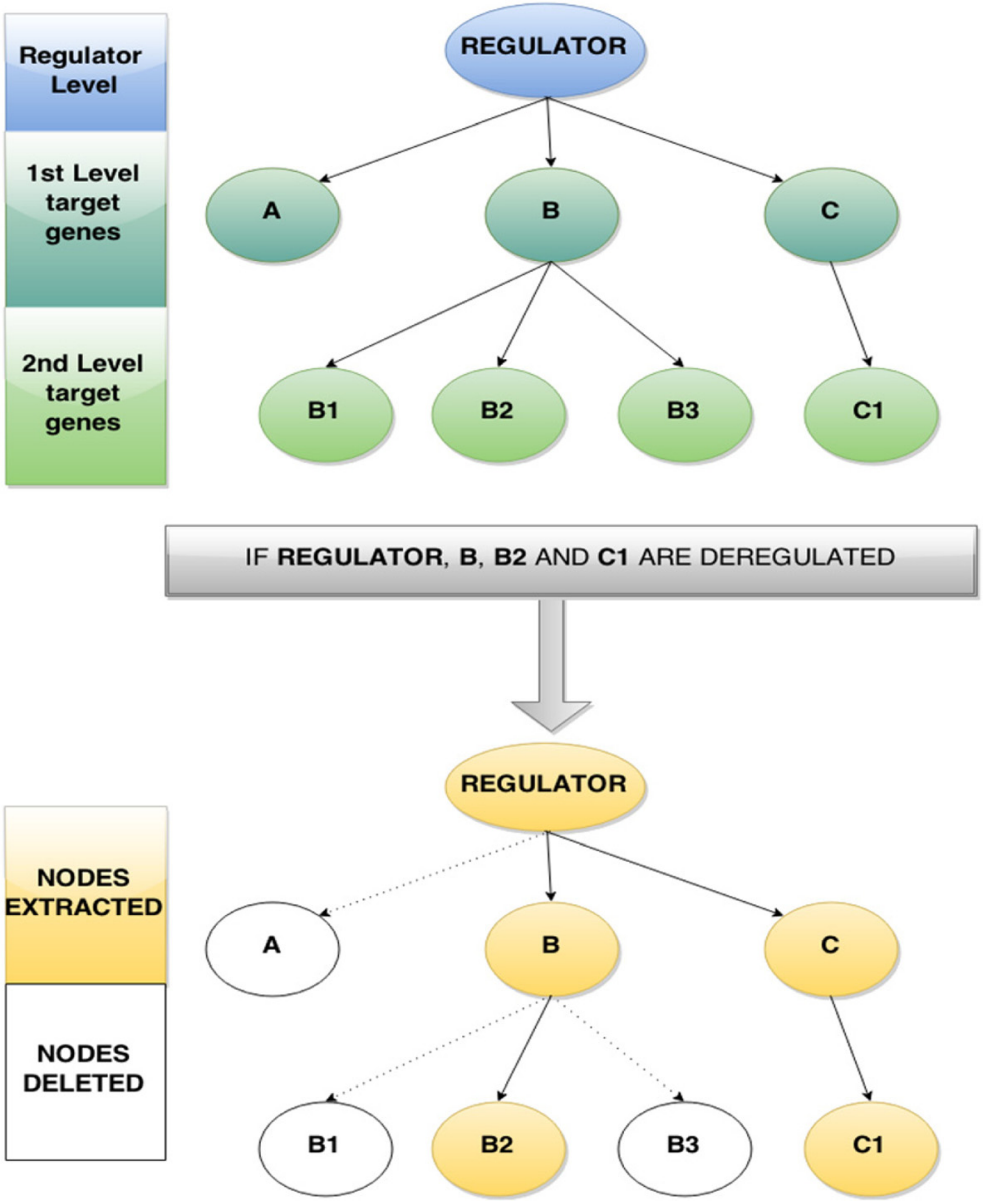
Workflow of the regulatory network inference. For each TF, a two-step profile of its targets is created. This profile includes the TF’s targets and its targets’ targets. If a TF is differentially expressed the regulatory subnetwork inferred consists of two types of interactions. First, the TF/target gene pairs when both TF and target gene are differentially expressed (e.g. Regulator-B interaction) and the TF/target-gene/target-of-target-gene when TF and target of target are differentially expressed, regardless of the expression status of the first-level target (e.g. Regulator-B-B2, Regulator-C-C1). See Methods for more details

In this way, a possibly “hidden” layer of regulation is included in the network based on the inference of combined differential expression that is assumed to be hierarchical (see Fig. 4 for details).

4. The size of the profile and the number of deregulated genes in the profile are used for the statistical calculation of the enrichment as described above.

Our final goal is to capture the hierarchical structure of gene expression regulation and impart more depth in the regulatory network extracted. We chose to constrain our analysis to two hierarchical levels for two reasons: One is that a significant proportion of interactions (~70 % for human) are 1st-level interactions that lead directly from a regulator to a leaf end node in the network. It is thus reasonable to expect that a two-level approach will encompass the overwhelming majority of interactions. The second reason is that, by attempting to track the small proportion of higher-level (>2) interactions, we would inevitably incorporate a great number of cyclic-interactions in the parsing of the network. This would bring about a significant slowing down of the whole process. Restricting our analysis to two levels we thus achieve the most efficient ratio of retrieved information over processing time. RNEA progressively builds a flatfile, which contains a subset of the initial reference “super-network”. This may be directly visualized through open-source network visualization and analysis platforms. Images presented in this paper were produced with Cytoscape [22]. One of the advantages of RNEA compared to other similar approaches is that it may incorporate protein and miRNA regulators in the same network. This is achieved via the incorporation of information from both transcriptional regulator and miRNA interactions.

### Running RNEA

RNEA is implemented as an R package which may be downloaded from https://sites.google.com/a/fleming.gr/rnea/home. It only requires one additional R package called “SortableHTMLTables” which is used in order to report the results in HTML format. RNEA currently accepts HUGO and RefSeq gene names as gene identifiers. It is also advised that users report differential expression as log_2_(fold-change) values in accordance with standard software. Nevertheless, the user may analyze data sets with non-standard differential expression values as long as he suitably adjusts the corresponding parameters. Both fold-change and p-value thresholds are set by the user, since quite often criteria need to be relaxed or made stricter in order to result in a reasonable number of differentially expressed genes that is sufficient for a statistical functional analysis.

The selection of the type of the identifier is done by the user with the use of an argument (Identifier) which can either be “GeneName”(default) or “Refseq”. Default usage produces tables of enriched GO categories, KEGG Pathways, Transcription Factors and miRNA that may be directly visualized on a browser as html files. Network reconstruction may be conducted in two different ways:

1. A “global” network that includes TF, miRNA and functional categories connected with their respective gene members that are enriched in differentially expressed genes. The goal of this approach is to extract central regulatory or functional components with the use of Network properties. The combined regulatory/functional network is extracted with the use of the network = “global” argument.
2. Only functional or only regulatory networks can be extracted with the use of the respective setting “functional”, or “regulatory” of the network argument. Given that RNEA’s main aim is producing concise and summarized regulatory networks, “regulatory” is the default argument.

Other parameters include the species from which expression values have been obtained (“Human”, “Mouse”) and the output type (“html” or “csv”).

More details may be found here: https://sites.google.com/a/fleming.gr/rnea/manual.

## Results

Inferring important regulators from genome-wide expression experiments is a complex problem. There are only a couple of available tools which may help in the definition of master regulators mostly by finding over-represented binding motifs in deregulated genes. TFactS is the only tool using a similar enrichment approach to ours, but it has significant limitations in terms of the number of studied regulators. TRRUST [16] represents a recent attempt to provide a golden standard, against which regulatory networks may be tested. Nevertheless, the variability of expression programs is immense and the underlying complexity of gene regulation suggests that very different networks may be produced with even mild changes in cellular conditions. In this sense, already available networks can only serve as providing the “reference” interactions, among which each condition may choose combinatorially. RNEA’s aim is providing a framework for revealing such combinations of known interactions. Its distinguishing features are that a) it focuses on well-documented regulatory interactions b) it aims at capturing the hierarchical structure of the network by the two-level scanning of regulator profiles and c) that it incorporates miRNA and protein regulators in a common regulatory network (see Methods). From the application’s point of view, RNEA is rather straightforward and in principle can be run with only a small list of prerequisites.

RNEA is able to infer, in a single run, both regulatory and functional enrichments from raw differential expression data. To date there are only a couple of similar methodologies whose scope however differs from RNEA. SPIA [27] implements a perturbation analysis to infer the significance of a given pathway based on the differential expression of its genes, while taking into account the topology of the pathway network. In this sense, it aims at a better and more accurate assessment of pathway deregulation based on the reported interactions and its final output is a list of deregulated pathways. PARADIGM [28] employs a similar approach through the additional incorporation of multiple omics and genetic data. Finally GGEA [29] is similar to our approach in terms that it combines gene expression and regulatory interactions aiming at an initial refinement of differentially expressed genes list, which it then uses for a gene set enrichment analysis. Compared to the above, our method’s distinguishing characteristic is that it projects DE gene lists on a reference map of regulatory interactions to infer a subnetwork of relevance to the particular gene expression profile. Providing the subnetwork reconstruction as primary output is thus RNEA’s particular feature, but the lack of similar approaches makes its cross-validation quite difficult. In order to assess its predictive power we have here applied it in cases of well-defined systems, for which there is documented knowledge of the underlying regulatory program. This is an approach also undertaken by the aforementioned methods [27, 29]. In this context, we chose to validate our methodology in two publicly available datasets originating from the two species for which RNEA provides information, a genome wide expression profile of cancerous versus normal human colon tissue samples and a gene profile of mouse RAW264.7 macrophage cells, stimulated with LPS.

In the following section, we briefly present the results of the analysis of the 2 test cases conducted with RNEA. As our method focuses primarily on transcriptional regulation we were more interested in assessing the robustness of the regulatory networks. In this sense, we attempted to validate the primary nodes of the deduced networks from the existing literature.

### Human test case

Colorectal cancer is the third most common type of cancer and the second most common cause of cancer death in the human population [30]. Despite a considerable amount of evidence delineating biological pathways related to the disease, the characterization of regulatory networks in cancer remains an open problem due to the great number of disease subtypes and the overall variability of the phenotypes.

In an attempt to predict a regulatory subnetwork for colorectal cancer, we obtained genome-wide expression data from 104 patients and 46 healthy individuals, that were normalized with robust multi-array average (RMA) and presented in a form suitable for RNEA analysis (log2(FC) of gene expression alongside the corresponding *p*-values) from [31] (GEO Accession: GSE21510). Patients with distant metastases were selected in order to assess the metastatic potential conferred to the disease by candidate gene markers. Using standard fold-change and p-value thresholds we obtained 148 DE genes. The functional analysis conducted with RNEA resulted in a number of deregulated GO terms and KEGG pathways that may be accessed in Additional file 1. Below we focus on the regulatory analysis.

**Fig. 5:**
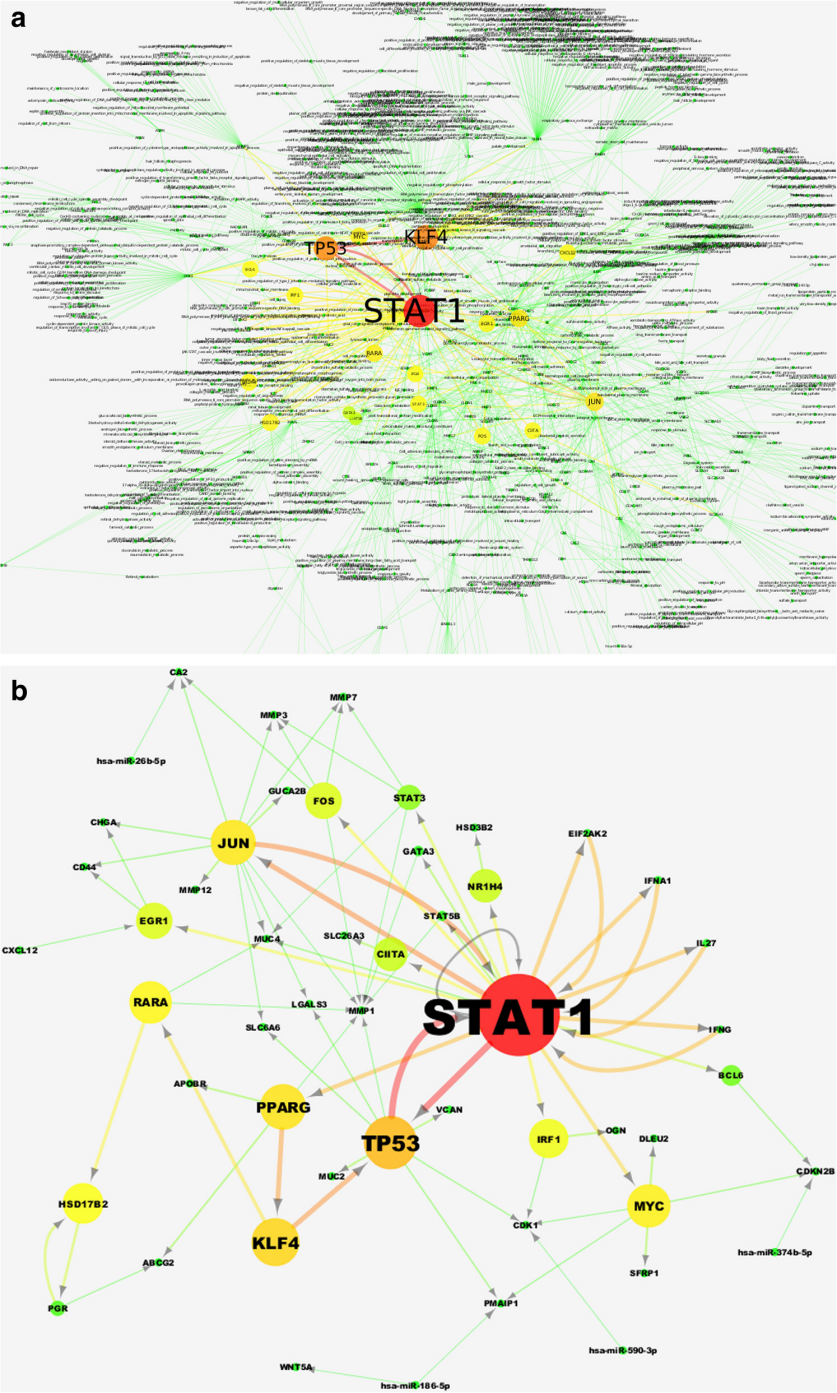
**a** Global (regulatory/functional) network for the human test case colorectal cancer. It includes the regulatory network extracted (as described in Fig. 1) and the functional categories (GOs and Kegg pathways) which are enriched (*P*-value Cutoff = 0.05) in DE genes. Node color and size and node label font size are visualized based on Betweenness centrality, which is an indicator of the centrality of a node in a network. **b** Regulatory network for the human test case of colorectal cancer. Node color and size and node label font size are visualized based on betweenness centrality, which is a measure of the centrality of a node in a network

The resulting global network, (including transcriptional regulators, miRNA and GO categories) is depicted in Fig. 5a. A major module of regulators that is central to the network is formed by STAT1, KLF4 and TP53, (node size and color is dependent on the betweenness centrality of each node). This figure is representative of the detail that most existing tools of functional analysis may confer, the degree of which makes the interpretation of the results rather complex difficult. In Fig. 5b, we present the subnetwork obtained from regulatory interactions alone. In this, we see a positive feedback loop between TP53 and STAT1 being in close connection with KLF4. STAT1 has been shown to stimulate inflammation in tumor cells and to trigger anti-proliferative and pro-apoptotic response [32] a role that is compatible with its interaction with TP53 and its well-known anti-oncogenic activity [33]. STAT3, another member of our network has also been heavily involved in cancers where STAT1 is upregulated. KLF4 is also particularly interesting as it is known to be an epithelial-specific transcription factor that is mainly active in the gastrointestinal tract [34]. The fact that it holds a central position in our network may come as a strong indication of RNEA’s ability to infer tissue-specificity from gene expression profiles. At another level, KLF4 upregulation has been shown to correlate with the degree of differentiation of normal cells to cancerous ones [34] and has been considered a marker of poor survival in CRC patients [35] which makes it even more important in the examined setting, where the majority of the cases involved distant metastases.

At the periphery of our network, apart from a set of rather expected regulators whose relation to cancer is well known (FOS, JUN, EGR1 and MYC) we find a set of secondary metabolism related genes including HSD17B2, a gene that is involved in lipid biosynthesis that has been shown to have a prognostic role in colorectal cancer [36]. At the same time all of the reported miRNA in the network have been reported to have a role in colorectal cancers. In particular, hsa-miR-26b-5p has been shown to exhibit a tumor suppressive role [37], hsa-miR-590-3p has been also known to have increased expression in colorectal cancers [38] while hsa-miR-374a-5p has been reported to be beneficial for the prognosis [39]. An important aspect of RNEA may be seen here. RNEA by default performs a dual enrichment analysis separately for over- and under-expressed gene targets. As expected, all miRNA-genes with significant enrichments in this setting are enriched primarily towards under-expressed targets. We consider this feature of great importance since a number of existing methods merely report enrichment on the basis of differential expression, regardless of the direction of regulation (activation or suppression). In certain cases however (miRNA regulation being a very clear one) this direction should be taken into account.

### Mouse test case

We next analyzed a test case for the mouse genome. We chose a well-described process of an external stimulus that is expected to show an inflammatory response, which we indeed observe through our functional analyses. We examined an experiment that aimed to analyze the differences in gene expression of the inflammatory response in RAW264.7 murine macrophage cells under stimulation by LPS [40] (GEO Accession: GSE63889). Using relatively stringent criteria on differential expression fold-change and p-value (absolute log_2_FC > =1, *p*-value < =0.05) we ended up with 121 differentially expressed genes, which were shifted towards over-expression with a ratio close to 2:1. This is to be expected given that LPS stimulation of immune cells is known to bring about an acute generalized response through the activation of a number of pathways. Functional analysis at the level of GO and KEGG pathways showed these pathways to match the expectations. In this way, inflammatory pathways including the TNF-NFkB signaling axis, cytokine and TLR signaling and a range of various infection-responsive pathways were strongly enriched in upregulated genes (see Additional file 2 for the complete lists).

**Fig. 6:**
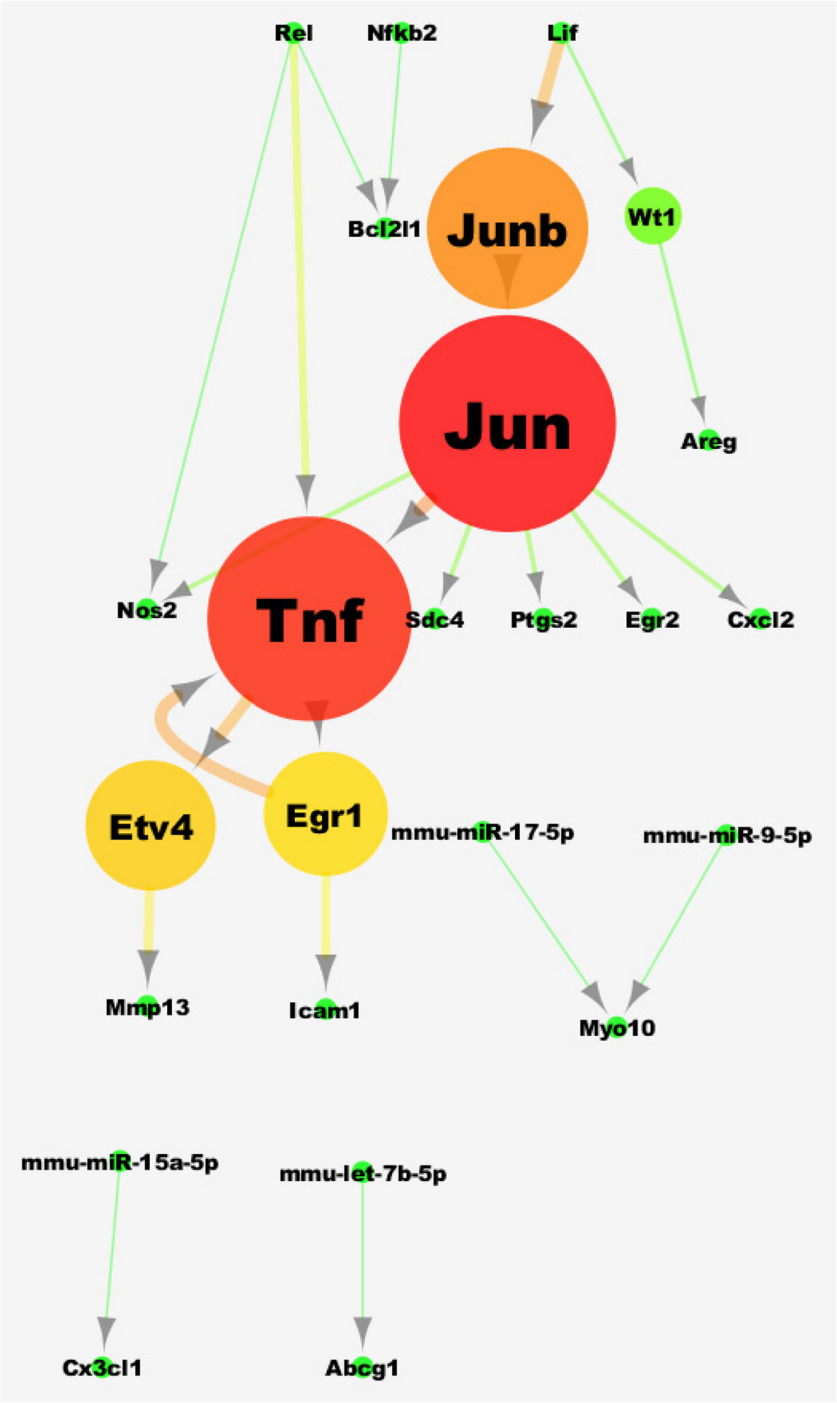
Regulatory network for the mouse test case of LPS-induced macrophages. The layout of the network is hierarchical. Node color and size and node label font size are visualized based on betweenness centrality, which is an indicator of the centrality of a node in a network

The resulting regulatory subnetwork for this experiment may be seen in Fig. 6. As in Fig. 5b, it only contains transcriptional regulators and miRNAs. The network is smaller than the one obtained in the human case for two reasons. Firstly because the mouse reference network, from which interactions are selected, is smaller than the human one (almost 3.5 times smaller). Secondly, it is reasonable to expect that the stimulation of macrophages by LPS brings about a much more concentrated response than the overall changes taking place in a complex disease such as cancer. The central role of Tnf is obvious in this network, as is a strong feed-forward loop between Tnf and Egr1. Such an interaction has been reported [41] for the same sort of LPS activation we are analyzing here. After Tnf, another important node (of high degree) in the network corresponds to Jun which has been known to mediate the effect of Nfkb in the activation of the inflammatory response [42]. More peripheral nodes in the network include Nfkb itself, Rel and Lif all of which are related to the cytokine-related response. miRNA genes with enriched targets in this network include mmu-miR-17-5p and mmu-miR-9-5p. Both of these miR species have been shown to be implicated in the mediation of inflammatory signaling although their role appears to be contradictory [43, 44].

## Discussion

Reconstructing the hierarchy of gene regulation is an open question of great importance in modern molecular biology, one that has not been effectively answered by the advent of next-generation sequencing technologies and genome-wide interrogation of gene expression. The main reasons for this, besides the inherent complexity of this dynamic process have been a) the scarcity of high-quality data (or -in contrast- the considerable amount of noisy information) connecting transcription factors and their gene targets and b) the lack of user-friendly methods summarizing the output of genome-wide expression experiments in a way that will allow for a fast and meaningful, first-level inspection of the data. The main goal of the presented work lies on these two axes: On one hand it aims to produce a compendium of transcription factor–target genes interactions that would be as reliable as possible, on the other, to implement these relationships, alongside other meaningful, functional information in a simple approach that would allow biologists to perform a quick overview of a gene expression experiment, prioritizing their results and putting the spotlight on highly significant regulatory interactions.

A major limitation of genome-scale experiments has, since very early, been a lack of summarization in their analysis. The production of enormous lists of genes, enriched processes and functions often results in complicating, rather than assisting in, their interpretation. Key aspects in the process of extracting knowledge from a large-scale experiment are related to a) efficient summarization, i.e. compiling the useful and significant information from a functional point of view and b) prioritization, the ranking, that is, of the relevant pieces of information in a way that will help focus on the most important facets of the results. RNEA addresses the problem of extensive lists by producing regulatory networks that combine prioritization and summarization of the observed enriched relationships. In this way, it reveals the genes that are important in the process of transcriptional regulation in the particular experimental setting. This may be done either through the assessment of the statistical enrichment of TF targets’ profiles or by evaluating network characteristics of the extracted regulatory subnetwork. Our work’s distinguishing feature is the output of an “active regulatory subnetwork”, which constitutes the most probable network of transcription factors being active in the studied condition, based on the gene expression values of their target genes. This also represents the major advantage of our pipeline, whose main goals are to detect and report regulatory information in the shape of regulatory networks that are of modest sizes, therefore allowing the user to easily interpret results and plan follow-up experiments.

When compared to other similar approaches (such as TFactS or Enrichr) our method’s main differences are related with the way it infers the regulatory hierarchy. RNEA does this by employing a two-level hierarchical approach, by adding at each TF profile the targets of its targets, if available. In this way TFs which are “higher” in the known hierarchy will have more targets in their profile and if an enrichment is found, it will add significance to the TF lying higher in the hierarchy. In addition, by checking for enrichment in both up-regulated and down-regulated genes in two different calculations, RNEA enables the investigation of possible dual roles for given TF, while at the same time to safeguard against contradictory annotation evidence that often assign inconsistent roles for a TF based on the literature.

Currently RNEA only supports human and mouse datasets. Given their relevance from the biomedical perspective these two organisms (human for obvious reasons and mouse due to the fact of being the most widely-used mammalian model organism) represent more than 90 % of the public repositories of gene interaction data. Their share of mammalian genome-wide expression profiles in gene expression databases is probably even higher. It was therefore reasonable to aim at the construction of reference networks for these two at a first level. Nevertheless, the incorporation of information on other organisms within the RNEA framework is expected to be quite straight-forward, given that a sufficient number of experimentally verified regulatory interactions are reported.

As a final comment, we should point out that RNEA deduces gene regulatory interactions directly from their noisy, highly complex end-product which is the relative abundance of mRNA molecules. In this regard, it is expected that the predicted networks will also be partly noisy and contain a reasonable amount of false positives. We, nonetheless believe, that being able to visualize a relevant network of interactions in a single step from your differential expression experiment makes up for a positive trade-off.

## Conclusions

RNEA is a framework for functional analysis of gene expression experiments, with a primary focus on gene regulatory relationships. It is easy to apply on standard gene expression read-outs, readily producing ranked lists of various functional groupings. Its key idea, though, is the derivation of a network of regulatory interactions. By creating regulatory subnetworks, RNEA enables a better overview of the regulatory process through direct visualization. RNEA benefits from (and also depends on) the accuracy of the prior knowledge used and the originality of network reconstruction. Most of the existing functional analysis tools mainly rely on computational predictions (through PWM) for the calculation of transcription factor target enrichments, while very few also employ experimental data from ChIP that are, however, still limited.

The main advantage of RNEA is the originality of the network approach. To our knowledge this is one of the few functional analysis tools that aims at the extraction of a regulatory subnetwork. Most of the existing approaches in this regard have been implemented in a sort of ‘personalized’ way, aiming at the interpretation of specific experiments, instead of proposing a generalized approach. Further validation of RNEA can mostly take place with extended use by the community, while refinements in the original reference networks are bound to increase its potential.

### Availability and requirements

**Project name:** Regulatory Network Enrichment Analysis (RNEA)

**Project home page:**https://sites.google.com/a/fleming.gr/rnea/home

**Operating system(s):** Multiple platforms

**Programming language:** R

**Other requirements:** R package “SortableHTMLTables**”** (http://cran.r-project.org/web/packages/SortableHTMLTables/index.html)

**License:** GNU GPLv3

**Any restrictions to use by non-academics:** None

## Additional files

Additional file 1: Human Test case results. Additional file 1 is a folder containing the detailed results of the Human Test case in HTML format. Each file includes the respective calculated enrichments for TFs, miRNAs, KEGG pathways, KEGG pathway categories and GO terms. In order to view the results a standard web-browser is needed (Chrome and Mozilla Firefox have been tested). The HTML must be opened from inside the folder because additional files (images and javascripts) which are needed for the correct view of the results are included. (ZIP 90 kb) Additional file 2: Mouse Test case results. Additional file 2 is a folder containing the detailed results of the Mouse Test case in HTML format. Each file includes the respective calculated enrichments for TFs, miRNAs, KEGG pathways, KEGG pathway categories and GO terms. In order to view the results a standard web-browser is needed (Chrome and Mozilla Firefox have been tested). The HTML files must be opened from inside the folder because additional files (images and javascripts) which are needed for the correct view of the results are included. (ZIP 83 kb)
