# Supplementary material for: Inferring active regulatory networks from gene expression data using a combination of prior knowledge and enrichment analysis
Source: BMC Bioinformatics. 2016 Jun 6;17(Suppl 5):181. doi: 10.1186/s12859-016-1040-7 (PMC4905609; doi:10.1186/s12859-016-1040-7)
Supplement: Additional file 1: — Human Test case results. Additional file 1 is a folder containing the detailed results of the Human Test case in HTML format. Each file includes the respective calculated enrichments for TFs, miRNAs, KEGG pathways, KEGG pathway categories and GO terms. In order to view the results a standard web-browser is needed (Chrome and Mozilla Firefox have been tested). The HTML must be opened from inside the folder because additional files (images and javascripts) which are needed for the correct view of the results are included. (ZIP 90 kb) [file 12859_2016_1040_MOESM1_ESM.zip › AdditionalFiles1/GSE21510 GO_Enrichment.html]

GSE21510 GO\_Enrichment


| GO | DE\_qvalue | UP\_qvalue | DOWN\_qvalue |
| --- | --- | --- | --- |
| protein\_binding | 0.157816373434129 | 0.243107358290672 | 0.15575328546077 |
| nucleus | 0.902175162220963 | 0.99968071961152 | 0.0321681451196331 |
| integral\_to\_membrane | 0.00366572328449652 | 0.00125004296735786 | 0.413614896355459 |
| cytoplasm | 0.860430389692979 | 0.750329049354271 | 0.663523404965354 |
| plasma\_membrane | 0.000437057278156203 | 4.90905391688342e-05 | 0.331766265337078 |
| cytosol | 0.0752179362138937 | 0.1807629456722 | 0.0613571224936216 |
| metal\_ion\_binding | 0.856562118843062 | 0.862623707264844 | 0.528821159424952 |
| transcription\_\_DNA-dependent | 0.901737224964499 | 0.992351983983517 | 0.106926370453535 |
| DNA\_binding | 0.998341107356082 | 0.99968071961152 | 0.648323762755459 |
| extracellular\_region | 0.00266422514859511 | 0.022085088918651 | 0.0203948830732625 |
| ATP\_binding | 0.723108645576218 | 0.913334691387682 | 0.18927811119529 |
| small\_molecule\_metabolic\_process | 0.000440511814719415 | 0.0012376030176624 | 0.0359919482372287 |
| regulation\_of\_transcription\_\_DNA-dependent | 0.823482407702054 | 0.986095689892992 | 0.133137301785025 |
| zinc\_ion\_binding | 0.00318729002303362 | 0.0115014309062954 | 0.0512647557254706 |
| mitochondrion | 0.924892179527804 | 0.976503130096018 | 0.369186486140516 |
| membrane | 0.0238433345652865 | 0.107944752099081 | 0.0401471640742829 |
| integral\_to\_plasma\_membrane | 0.00543778856542623 | 0.0356500984892257 | 0.0266039259874058 |
| signal\_transduction | 0.575221791297948 | 0.507117842414435 | 0.483530008081256 |
| nucleoplasm | 0.684872643711459 | 0.940406911773 | 0.0517207666599693 |
| sequence-specific\_DNA\_binding\_transcription\_factor\_activity | 0.475987525870165 | 0.613742647122654 | 0.100480619538647 |
| nucleic\_acid\_binding | 0.886237405411159 | 0.924362224987531 | 0.415146549431468 |
| extracellular\_space | 0.020134293229218 | 0.107944752099081 | 0.0293951888063814 |
| intracellular | 0.659269240711799 | 0.880271916446503 | 0.156948343218054 |
| Golgi\_apparatus | 0.0411075167774621 | 0.033437545765977 | 0.285832936807016 |
| innate\_immune\_response | 0.57251507946392 | 0.42984295616594 | 0.549814825299376 |
| positive\_regulation\_of\_transcription\_from\_RNA\_polymerase\_II\_promoter | 0.0190907717183401 | 0.0671603898911067 | 0.0189143619162296 |
| calcium\_ion\_binding | 0.0619172521295732 | 0.228320252564269 | 0.0166979583284687 |
| gene\_expression | 0.689202161774174 | 0.807803231805149 | 0.248659577867032 |
| endoplasmic\_reticulum\_membrane | 0.0559853894877794 | 0.111278925651044 | 0.10288482809922 |
| endoplasmic\_reticulum | 0.0437288223239844 | 0.0442223918322977 | 0.224272574004957 |
| RNA\_binding | 0.815183281294929 | 0.944117915478061 | 0.22030445144582 |
| nucleolus | 0.639119660917689 | 0.546204293342025 | 0.22030445144582 |
| apoptotic\_process | 0.40149485465875 | 0.752542693780095 | 0.07878659371612 |
| protein\_homodimerization\_activity | 0.0119075517078797 | 0.0294946940892483 | 0.0255471728442487 |
| transmembrane\_transport | 0.00171877292184045 | 0.00941732172274037 | 0.0220365928654085 |
| sequence-specific\_DNA\_binding | 0.499610938387094 | 0.692407618431277 | 0.159332943203595 |
| negative\_regulation\_of\_transcription\_from\_RNA\_polymerase\_II\_promoter | 0.166740301802308 | 0.240099992598596 | 0.0572126810679413 |
| cellular\_protein\_metabolic\_process | 0.0377286338904717 | 0.0497569970718571 | 0.154647788052755 |
| proteolysis | 0.00681693473010034 | 0.112266052254303 | 0.00622134022169701 |
| positive\_regulation\_of\_transcription\_\_DNA-dependent | 0.141016724218929 | 0.215487252727971 | 0.049934907124938 |
| negative\_regulation\_of\_apoptotic\_process | 0.01324798743301 | 0.214654737873569 | 0.0052201455943604 |
| multicellular\_organismal\_development | 0.259274737313077 | 0.393064525222795 | 0.138443100892352 |
| immune\_response | 0.853095478331578 | 0.651346707139513 | 0.706528058577927 |
| perinuclear\_region\_of\_cytoplasm | 0.0635717459545091 | 0.040667367178467 | 0.347927931692278 |
| blood\_coagulation | 0.415435663410327 | 0.375677304244336 | 0.336312729073989 |
| Golgi\_membrane | 0.118462583271287 | 0.0867179047836939 | 0.334096754270999 |
| cell\_adhesion | 0.0218996165475733 | 0.18168052503448 | 0.0133875312609887 |
| negative\_regulation\_of\_transcription\_\_DNA-dependent | 0.213565949723128 | 0.35251911321252 | 0.119494035682986 |
| positive\_regulation\_of\_cell\_proliferation | 0.0841695679057894 | 0.0650932128506034 | 0.289537589805137 |
| protein\_transport | 0.305201018813247 | 0.13547489424663 | 0.632322389422061 |
| cell\_differentiation | 0.291672948146622 | 0.283462143707212 | 0.261872596102275 |
| mitotic\_cell\_cycle | 0.0259283570343021 | 0.529128764562216 | 0.00193925639806166 |
| identical\_protein\_binding | 0.00386931702879394 | 0.0186969747319295 | 0.0266536251084543 |
| protein\_heterodimerization\_activity | 0.270634548021767 | 0.266244546134243 | 0.249246877050966 |
| cell\_junction | 0.730463876950873 | 0.515242248776305 | 0.60950275387032 |
| negative\_regulation\_of\_cell\_proliferation | 0.0226403923145975 | 0.046564993789673 | 0.081812239556857 |
| protein\_serine/threonine\_kinase\_activity | 0.264465080963183 | 0.511690935982581 | 0.0814717170143954 |
| intracellular\_membrane-bounded\_organelle | 0.261773926578425 | 0.509139747263677 | 0.0805112613264025 |
| transport | 0.00285418701083971 | 0.00478412095789432 | 0.0768437444558309 |
| cell\_surface | 0.0190907717183401 | 0.104677372412662 | 0.0228107377550087 |
| nucleotide\_binding | 0.440674360348958 | 0.794858085933194 | 0.0736964718710755 |
| cytoskeleton | 0.440674360348958 | 0.240490542375663 | 0.586259109790313 |
| small\_GTPase\_mediated\_signal\_transduction | 0.696485483791902 | 0.794614816611989 | 0.226544977879771 |
| carbohydrate\_metabolic\_process | 0.000440511814719415 | 0.0041907646433162 | 0.0061836651171608 |
| centrosome | 0.694019578887505 | 0.479355262166923 | 0.583583936288505 |
| spermatogenesis | 0.687410117982756 | 0.788412648719048 | 0.22056510223172 |
| cell\_proliferation | 0.100453018828287 | 0.469116066053342 | 0.0203948830732625 |
| protein\_phosphorylation | 0.419128629517925 | 0.785074569576088 | 0.0688207702737849 |
| phospholipid\_binding | 0.682242958594349 | 0.785074569576088 | 0.21777748648411 |
| virus-host\_interaction | 0.039656032255673 | 0.0928697155646234 | 0.0203948830732625 |
| mitochondrial\_inner\_membrane | 0.2143788894228 | 0.22575289610606 | 0.21604060394111 |
| chromatin\_binding | 0.414171032502744 | 0.461517550118684 | 0.215275762315107 |
| transcription\_from\_RNA\_polymerase\_II\_promoter | 0.404943818924505 | 0.453848929452233 | 0.21082310914324 |
| axon\_guidance | 0.198452208952597 | 0.444902244893935 | 0.0632086651863861 |
| G-protein\_coupled\_receptor\_signaling\_pathway | 0.186218383565194 | 0.432544330754211 | 0.0593872298345937 |
| viral\_reproduction | 0.639119660917689 | 0.758233884918424 | 0.194934489529186 |
| actin\_binding | 0.365383898941678 | 0.422859776315049 | 0.191816677462773 |
| cell\_division | 0.167530519801367 | 0.747313836123152 | 0.0157470356191901 |
| Fc-epsilon\_receptor\_signaling\_pathway | 0.61264592531375 | 0.743106021104764 | 0.182650075953045 |
| protein\_kinase\_binding | 0.335191258142617 | 0.17813601684484 | 0.521709917365295 |
| receptor\_binding | 0.326256932418516 | 0.390744825631548 | 0.174070624764913 |
| intracellular\_signal\_transduction | 0.593937229382479 | 0.390744825631548 | 0.515967506675717 |
| nervous\_system\_development | 0.325212449625466 | 0.389772544371509 | 0.17344864097479 |
| transcription\_factor\_binding | 0.321864242774394 | 0.727247528319588 | 0.0485645165914354 |
| DNA\_repair | 0.146410364666784 | 0.727247528319588 | 0.0133511432485421 |
| protein\_kinase\_activity | 0.586585435422955 | 0.727247528319588 | 0.170733852631221 |
| response\_to\_drug | 0.00681693473010034 | 0.0605897900726602 | 0.0125655770024194 |
| nerve\_growth\_factor\_receptor\_signaling\_pathway | 0.575221791297948 | 0.719932773867105 | 0.166314692522733 |
| extracellular\_matrix\_organization | 7.75394770129854e-06 | 0.0562256293167479 | 9.40461796434864e-07 |
| translation | 0.553889128348871 | 0.705318127721401 | 0.156752442315663 |
| RNA\_metabolic\_process | 0.550173620708925 | 0.703111045350932 | 0.155318035357558 |
| positive\_regulation\_of\_apoptotic\_process | 0.117615957043739 | 0.347916989298042 | 0.0404454347595913 |
| regulation\_of\_transcription\_from\_RNA\_polymerase\_II\_promoter | 0.522540624505022 | 0.685151450295046 | 0.14464247870541 |
| ubiquitin-protein\_ligase\_activity | 0.514145353121429 | 0.329783169165352 | 0.464173856079435 |
| RNA\_splicing | 0.255145055739244 | 0.329783169165352 | 0.141931763363668 |
| transcription\_factor\_complex | 0.0377286338904717 | 0.328204049810593 | 0.00914754077829022 |
| cell-cell\_signaling | 0.510142423574721 | 0.676508047843168 | 0.140577185053981 |
| signal\_transducer\_activity | 0.245567748986321 | 0.126907149382215 | 0.13817088226687 |
| mitosis | 0.035221648787957 | 0.669206718853103 | 0.00177502171445304 |
| enzyme\_binding | 0.0931926621604729 | 0.121054719666 | 0.0331021265784926 |
| protein\_complex | 0.485964505236387 | 0.310911651494093 | 0.446281427057335 |
| neuronal\_cell\_body | 0.484096301500141 | 0.658417686317297 | 0.130815541498844 |
| structural\_molecule\_activity | 0.00983754985174113 | 0.0112785183322565 | 0.126584169106876 |
| mRNA\_metabolic\_process | 0.473282416496806 | 0.651261897068785 | 0.126584169106876 |
| cell\_cycle | 0.215955719372901 | 0.299022067395431 | 0.125212643356441 |
| receptor\_activity | 0.209800395613964 | 0.107944752099081 | 0.429232735283136 |
| apical\_plasma\_membrane | 5.99865296038343e-05 | 2.17768346973387e-05 | 0.120831858687755 |
| in\_utero\_embryonic\_development | 0.206407178707678 | 0.29010276325963 | 0.120035734336316 |
| cytokine-mediated\_signaling\_pathway | 0.0789628477982523 | 0.106731574168344 | 0.0294760801726734 |
| catalytic\_activity | 0.438521313042102 | 0.625548698415875 | 0.114595387758848 |
| proteinaceous\_extracellular\_matrix | 3.53256268791485e-10 | 0.000123617779018345 | 1.96316074386236e-06 |
| transporter\_activity | 0.0684177000787213 | 0.0294946940892483 | 0.409322978649872 |
| dendrite | 0.184502399811538 | 0.0947923081417909 | 0.109486573830615 |
| angiogenesis | 0.184502399811538 | 0.268483605795361 | 0.109486573830615 |
| visual\_perception | 0.162483371594971 | 0.59071463875213 | 0.023608304798247 |
| hydrolase\_activity | 0.0560096403177509 | 0.0823182947079 | 0.0982402135035233 |
| epidermal\_growth\_factor\_receptor\_signaling\_pathway | 0.388298592850217 | 0.587296654772713 | 0.0982402135035233 |
| post-translational\_protein\_modification | 0.0177210556651111 | 0.00625123042649873 | 0.383055683474486 |
| transcription\_initiation\_from\_RNA\_polymerase\_II\_promoter | 0.380973750127781 | 0.240851159557952 | 0.380196657681289 |
| mRNA\_processing | 0.380973750127781 | 0.240851159557952 | 0.380196657681289 |
| oxidoreductase\_activity | 0.0166315758116464 | 0.00600915163229803 | 0.379283778867147 |
| cellular\_nitrogen\_compound\_metabolic\_process | 0.379118903000893 | 0.23996444268208 | 0.379283778867147 |
| lysosome | 0.376895750135255 | 0.238540047755433 | 0.378363505606129 |
| magnesium\_ion\_binding | 0.150012280079154 | 0.575708341660641 | 0.021894283534668 |
| transcription\_corepressor\_activity | 0.372072669509975 | 0.574192842937323 | 0.0939521084425452 |
| actin\_cytoskeleton | 0.367226573226796 | 0.570588702347154 | 0.0924155050015092 |
| external\_side\_of\_plasma\_membrane | 0.00443487593179285 | 0.00540891407688083 | 0.0917077499754997 |
| brain\_development | 0.0455289812302614 | 0.0206866961762308 | 0.364257057585121 |
| ubiquitin-dependent\_protein\_catabolic\_process | 0.352220296983982 | 0.222802470498641 | 0.362927622981718 |
| regulation\_of\_apoptotic\_process | 0.347292179789209 | 0.219622105431603 | 0.0861656021108659 |
| nuclear\_mRNA\_splicing\_\_via\_spliceosome | 0.339700629407423 | 0.547646711212902 | 0.0842346500442612 |
| endoplasmic\_reticulum\_lumen | 0.0414377339352987 | 0.214654737873569 | 0.0187453497129373 |
| structural\_constituent\_of\_ribosome | 0.332390205503537 | 0.542064467134598 | 0.0820174122893952 |
| response\_to\_DNA\_damage\_stimulus | 0.332390205503537 | 0.542064467134598 | 0.0820174122893952 |
| growth\_factor\_activity | 0.039749496177993 | 0.0654050405800874 | 0.0820174122893952 |
| ion\_transport | 0.011564552029404 | 0.0175665270270088 | 0.0815294490803037 |
| protein\_dimerization\_activity | 0.120458043748813 | 0.538621635970472 | 0.0175790830156855 |
| electron\_carrier\_activity | 0.325344064114604 | 0.536880543507023 | 0.0804136969910517 |
| cytokine\_activity | 0.119199035566036 | 0.536880543507023 | 0.0173220259053119 |
| calmodulin\_binding | 0.325344064114604 | 0.536880543507023 | 0.0804136969910517 |
| transcription\_regulatory\_region\_DNA\_binding | 0.117815781588245 | 0.203342940754274 | 0.0798710937506276 |
| protein\_domain\_specific\_binding | 0.323603077819915 | 0.536141645819695 | 0.0798710937506276 |
| serine-type\_endopeptidase\_activity | 0.3215405241426 | 0.534371629414848 | 0.079192794075098 |
| nuclear\_membrane | 0.3215405241426 | 0.534371629414848 | 0.079192794075098 |
| protein\_C-terminus\_binding | 0.316774576228607 | 0.198838849118503 | 0.338042644435868 |
| protein\_autophosphorylation | 0.316774576228607 | 0.530791262368836 | 0.0778365606169034 |
| cell\_surface\_receptor\_signaling\_pathway | 0.316774576228607 | 0.530791262368836 | 0.0778365606169034 |
| Wnt\_receptor\_signaling\_pathway | 0.112800518890961 | 0.529485018349523 | 0.0166979583284687 |
| fibroblast\_growth\_factor\_receptor\_signaling\_pathway | 0.314990604920354 | 0.529485018349523 | 0.0771586712853036 |
| phospholipid\_metabolic\_process | 0.312588551840972 | 0.195820340550945 | 0.33518541155074 |
| iron\_ion\_binding | 0.111532267197956 | 0.195820340550945 | 0.076480963673346 |
| response\_to\_hypoxia | 0.00927498242536567 | 0.521601192189405 | 0.000438362094450586 |
| ubiquitin\_protein\_ligase\_binding | 0.302335027658247 | 0.189301330514883 | 0.329079142853801 |
| ribosome | 0.302335027658247 | 0.519721508055523 | 0.0738602385084637 |
| DNA\_replication | 0.105986788336397 | 0.519721508055523 | 0.0159534538915327 |
| G1/S\_transition\_of\_mitotic\_cell\_cycle | 0.102952439315454 | 0.515919786842394 | 0.0155127787819655 |
| carbohydrate\_binding | 0.0309637411555932 | 0.0547285455074714 | 0.0725933019971704 |
| axon | 0.102952439315454 | 0.0547285455074714 | 0.0725933019971704 |
| cellular\_lipid\_metabolic\_process | 0.294753580587443 | 0.514491330369857 | 0.0719163204700814 |
| xenobiotic\_metabolic\_process | 0.0291265263353709 | 0.0514487412316144 | 0.0698032878468208 |
| insulin\_receptor\_signaling\_pathway | 0.287139062390929 | 0.508634040400219 | 0.0698032878468208 |
| heart\_development | 0.0979500211676693 | 0.508634040400219 | 0.0146371235234648 |
| positive\_regulation\_of\_I-kappaB\_kinase/NF-kappaB\_cascade | 0.0966776660941545 | 0.17813601684484 | 0.0691285506400272 |
| defense\_response\_to\_virus | 0.0953084936225536 | 0.0497569970718571 | 0.0686208445177162 |
| lysosomal\_membrane | 0.276490613551668 | 0.174143264607277 | 0.311448796757177 |
| early\_endosome | 0.276490613551668 | 0.500135367070767 | 0.0670255621418952 |
| homophilic\_cell\_adhesion | 0.271558331180134 | 0.170445594788505 | 0.308095577588892 |
| endosome | 0.271558331180134 | 0.170445594788505 | 0.308095577588892 |
| basolateral\_plasma\_membrane | 0.00681693473010034 | 0.0119854113391728 | 0.0658059816180355 |
| heme\_binding | 0.26438888275611 | 0.487891566197013 | 0.0635148369628647 |
| induction\_of\_apoptosis | 0.023958679737401 | 0.16463852953148 | 0.0020107532278998 |
| synapse | 0.259413786513386 | 0.162879746973027 | 0.299465144031896 |
| positive\_regulation\_of\_gene\_expression | 0.0815316149929657 | 0.4784318108755 | 0.0123967027091727 |
| heparin\_binding | 0.0789628477982523 | 0.0425178473113011 | 0.292290293243524 |
| membrane\_raft | 0.243929882308185 | 0.152718385111042 | 0.289077012160321 |
| cell\_cycle\_arrest | 0.0762824410561132 | 0.152718385111042 | 0.0578338649509827 |
| skeletal\_system\_development | 0.0751422703637985 | 0.040415042604097 | 0.287458840297906 |
| double-stranded\_DNA\_binding | 0.00523908898521749 | 0.040415042604097 | 0.00174679992350269 |
| response\_to\_stress | 0.238680810466188 | 0.149615623836728 | 0.285832936807016 |
| cell\_migration | 0.225230230630938 | 0.450603366688419 | 0.0537991809009562 |
| focal\_adhesion | 0.0672932837149476 | 0.140572382518652 | 0.053075815882478 |
| transforming\_growth\_factor\_beta\_receptor\_signaling\_pathway | 0.219997389922799 | 0.139353823835447 | 0.272938765741816 |
| G2/M\_transition\_of\_mitotic\_cell\_cycle | 0.0665070365555143 | 0.139353823835447 | 0.0523551840199572 |
| kinase\_activity | 0.217490637588646 | 0.137779023328968 | 0.271256552146704 |
| translational\_initiation | 0.215196401578376 | 0.440303217568394 | 0.0515197940850162 |
| nuclear-transcribed\_mRNA\_catabolic\_process\_\_nonsense-mediated\_decay | 0.21310928929251 | 0.437904602176212 | 0.050866406180273 |
| ion\_transmembrane\_transport | 0.0631483755929781 | 0.0339345148029485 | 0.26761495002033 |
| chemotaxis | 0.21310928929251 | 0.134628741478654 | 0.26761495002033 |
| negative\_regulation\_of\_cell\_growth | 0.0619172521295732 | 0.132883104817329 | 0.0501486613176525 |
| lamellipodium | 0.210803911481308 | 0.43548885305067 | 0.0501486613176525 |
| sensory\_perception\_of\_sound | 0.0609167054083868 | 0.433055865734542 | 0.00901138567267291 |
| response\_to\_lipopolysaccharide | 0.208494133877704 | 0.433055865734542 | 0.0498256710728262 |
| glycosaminoglycan\_metabolic\_process | 2.49264010418302e-06 | 0.00153239898499489 | 0.000188851112592336 |
| response\_to\_virus | 0.20345392670356 | 0.128494413500897 | 0.260499587213241 |
| protein\_complex\_assembly | 0.20345392670356 | 0.428978065113718 | 0.0483947922609477 |
| embryo\_development | 0.058641893617954 | 0.128494413500897 | 0.0483947922609477 |
| nucleosome\_assembly | 0.201135817907145 | 0.426488672709523 | 0.0477472652404504 |
| central\_nervous\_system\_development | 0.0566771883458152 | 0.423981575107172 | 0.00836759523875031 |
| anchored\_to\_membrane | 0.000894897978066401 | 0.000312055232923089 | 0.256775506945287 |
| transmembrane\_signaling\_receptor\_activity | 0.193379621044849 | 0.41891383144649 | 0.0458756556671999 |
| regulation\_of\_cell\_proliferation | 0.193379621044849 | 0.41891383144649 | 0.0458756556671999 |
| mitochondrial\_outer\_membrane | 0.0134578652044055 | 0.122193763703457 | 0.00810587640271628 |
| leukocyte\_migration | 0.0547306117050315 | 0.122193763703457 | 0.0458756556671999 |
| cytoplasmic\_membrane-bounded\_vesicle | 0.193379621044849 | 0.122193763703457 | 0.253024660933667 |
| toll-like\_receptor\_signaling\_pathway | 0.191248338947899 | 0.416763977127484 | 0.0452948251169083 |
| positive\_regulation\_of\_NF-kappaB\_transcription\_factor\_activity | 0.188728990732536 | 0.414182829273938 | 0.0445939679508548 |
| defense\_response\_to\_bacterium | 0.0525403650310766 | 0.0284260891669219 | 0.249246877050966 |
| cell\_cortex | 0.0525403650310766 | 0.0284260891669219 | 0.249246877050966 |
| RNA\_polymerase\_II\_distal\_enhancer\_sequence-specific\_DNA\_binding\_transcription\_factor\_activity | 0.186218383565194 | 0.411583406983162 | 0.0441221322681718 |
| regulation\_of\_cell\_shape | 0.186218383565194 | 0.118255774589337 | 0.248173895538101 |
| metalloendopeptidase\_activity | 0.00081080394622004 | 0.117144672452652 | 0.000129025860666948 |
| SRP-dependent\_cotranslational\_protein\_targeting\_to\_membrane | 0.181733521397999 | 0.405926985776004 | 0.0429768435424607 |
| neuron\_migration | 0.179215008993505 | 0.403274300338785 | 0.0422827077990515 |
| tight\_junction | 0.00266422514859511 | 0.00517405369077716 | 0.0415921061779157 |
| protein\_glycosylation | 0.168652680307794 | 0.107944752099081 | 0.234086245918324 |
| ATPase\_activity | 0.168652680307794 | 0.391695898931483 | 0.0401503009718101 |
| cell-cell\_junction | 0.0102240546154767 | 0.0236874966481957 | 0.0395200823209101 |
| serine-type\_endopeptidase\_inhibitor\_activity | 0.163998520473913 | 0.104677372412662 | 0.230167622204785 |
| hormone\_activity | 0.0422599198102919 | 0.104677372412662 | 0.0388374302903553 |
| elevation\_of\_cytosolic\_calcium\_ion\_concentration | 0.163998520473913 | 0.386961424331949 | 0.0388374302903553 |
| toll-like\_receptor\_4\_signaling\_pathway | 0.159179696083526 | 0.380989254584005 | 0.0380192002190379 |
| structural\_constituent\_of\_cytoskeleton | 0.159179696083526 | 0.102103576800528 | 0.226437657448812 |
| aging | 0.159179696083526 | 0.380989254584005 | 0.0380192002190379 |
| activation\_of\_MAPK\_activity | 0.0397914105430462 | 0.378544688890156 | 0.00634174405190903 |
| intracellular\_protein\_kinase\_cascade | 0.154528865463552 | 0.375699758967576 | 0.0366636379184205 |
| viral\_infectious\_cycle | 0.152047595881007 | 0.373208909564631 | 0.0359919482372287 |
| translational\_elongation | 0.152047595881007 | 0.373208909564631 | 0.0359919482372287 |
| spliceosomal\_complex | 0.152047595881007 | 0.0976727942167496 | 0.22056510223172 |
| midbody | 0.0381703613245562 | 0.373208909564631 | 0.00602610432319336 |
| anatomical\_structure\_morphogenesis | 0.152047595881007 | 0.0976727942167496 | 0.22056510223172 |
| antigen\_processing\_and\_presentation\_of\_exogenous\_peptide\_antigen\_via\_MHC\_class\_II | 0.150035777948505 | 0.0961076879939061 | 0.219505597704948 |
| positive\_regulation\_of\_ERK1\_and\_ERK2\_cascade | 0.0352553748417856 | 0.364486375745824 | 0.00561548156771527 |
| glycerophospholipid\_biosynthetic\_process | 0.0352553748417856 | 0.0928697155646234 | 0.0346509416073682 |
| cell-cell\_adhesion | 0.145237630483286 | 0.364486375745824 | 0.0346509416073682 |
| microtubule\_organizing\_center | 0.0346622958897834 | 0.361902698536834 | 0.00545648820814656 |
| nuclear\_chromatin | 0.0337159474110229 | 0.0191824349562281 | 0.0333224748875649 |
| muscle\_organ\_development | 0.140309370122694 | 0.0902657772514643 | 0.211777958675674 |
| ubiquitin\_thiolesterase\_activity | 0.137855444483612 | 0.0885871144019389 | 0.210026925365821 |
| neuron\_differentiation | 0.137855444483612 | 0.355580305634154 | 0.0327131261183519 |
| positive\_regulation\_of\_protein\_phosphorylation | 0.0323088140220208 | 0.0875410945104363 | 0.0324013957871399 |
| positive\_regulation\_of\_angiogenesis | 0.13554993051383 | 0.352570245933897 | 0.0324013957871399 |
| integrin\_binding | 0.13554993051383 | 0.352570245933897 | 0.0324013957871399 |
| Golgi\_lumen | 2.02066719434876e-07 | 5.43453598136936e-06 | 0.00513675668473379 |
| cysteine-type\_peptidase\_activity | 0.13554993051383 | 0.0875410945104363 | 0.20806153499462 |
| trans-Golgi\_network | 0.13338681702366 | 0.0861158547970474 | 0.206086959121601 |
| phototransduction\_\_visible\_light | 0.13338681702366 | 0.0861158547970474 | 0.206086959121601 |
| translational\_termination | 0.131085540779004 | 0.347190124904892 | 0.0316090194717199 |
| viral\_transcription | 0.126096071136728 | 0.340665267673678 | 0.0302546392526199 |
| RNA\_processing | 0.126096071136728 | 0.340665267673678 | 0.0302546392526199 |
| MyD88-dependent\_toll-like\_receptor\_signaling\_pathway | 0.126096071136728 | 0.340665267673678 | 0.0302546392526199 |
| calcium\_ion\_transport | 0.126096071136728 | 0.0820877116216548 | 0.199912872447233 |
| spindle\_pole | 0.0285853584226894 | 0.338234094191426 | 0.00449637480950757 |
| PDZ\_domain\_binding | 0.124074055420517 | 0.0804280579603841 | 0.197902925271935 |
| DNA\_recombination | 0.124074055420517 | 0.338234094191426 | 0.0296995905010603 |
| negative\_regulation\_of\_canonical\_Wnt\_receptor\_signaling\_pathway | 0.00569880250432188 | 0.078777885905305 | 0.0043536035482474 |
| lipid\_catabolic\_process | 0.121791510087835 | 0.078777885905305 | 0.195883604207493 |
| cellular\_response\_to\_hypoxia | 0.0279000531572217 | 0.078777885905305 | 0.0294760801726734 |
| cell-matrix\_adhesion | 0.0279000531572217 | 0.078777885905305 | 0.0294760801726734 |
| anaphase-promoting\_complex-dependent\_proteasomal\_ubiquitin-dependent\_protein\_catabolic\_process | 0.0279000531572217 | 0.33543010767384 | 0.0043536035482474 |
| toll-like\_receptor\_3\_signaling\_pathway | 0.119513967704522 | 0.332259549266143 | 0.0293212071893087 |
| extracellular\_matrix\_disassembly | 0.000317726813356996 | 0.332259549266143 | 1.96316074386236e-06 |
| activation\_of\_cysteine-type\_endopeptidase\_activity\_involved\_in\_apoptotic\_process | 0.119513967704522 | 0.332259549266143 | 0.0293212071893087 |
| sphingolipid\_metabolic\_process | 0.117615957043739 | 0.0768672750881787 | 0.191816677462773 |
| response\_to\_ethanol | 0.117615957043739 | 0.329783169165352 | 0.0287154346130511 |
| PML\_body | 0.117615957043739 | 0.329783169165352 | 0.0287154346130511 |
| MyD88-independent\_toll-like\_receptor\_signaling\_pathway | 0.117615957043739 | 0.329783169165352 | 0.0287154346130511 |
| kidney\_development | 0.117615957043739 | 0.0768672750881787 | 0.191816677462773 |
| cytokinesis | 0.117615957043739 | 0.329783169165352 | 0.0287154346130511 |
| apical\_part\_of\_cell | 0.0263539235840674 | 0.0148161564195688 | 0.191816677462773 |
| sodium\_ion\_transport | 0.0255394189476924 | 0.0146767641324501 | 0.190327681655684 |
| lung\_development | 0.115961164721536 | 0.328204049810593 | 0.0282032116155758 |
| TRIF-dependent\_toll-like\_receptor\_signaling\_pathway | 0.113561491827898 | 0.32528205918233 | 0.0275584569518714 |
| positive\_regulation\_of\_sequence-specific\_DNA\_binding\_transcription\_factor\_activity | 0.113561491827898 | 0.0735822593283713 | 0.18845006042972 |
| canonical\_Wnt\_receptor\_signaling\_pathway | 0.0249551118408792 | 0.0735822593283713 | 0.0275584569518714 |
| basement\_membrane | 0.113561491827898 | 0.0735822593283713 | 0.18845006042972 |
| regulation\_of\_ubiquitin-protein\_ligase\_activity\_involved\_in\_mitotic\_cell\_cycle | 0.0241240144443221 | 0.321998804180471 | 0.00377832852241083 |
| male\_gonad\_development | 0.00104751858502909 | 0.013787662255545 | 0.00377832852241083 |
| cell\_junction\_assembly | 0.111532267197956 | 0.0721732877551033 | 0.186559466916747 |
| ATP\_catabolic\_process | 0.111532267197956 | 0.321998804180471 | 0.0269624182699873 |
| RNA\_polymerase\_II\_core\_promoter\_proximal\_region\_sequence-specific\_DNA\_binding\_transcription\_factor\_activity\_involved\_in\_positive\_regulation\_of\_transcription | 0.109383766727661 | 0.0720818760422794 | 0.184473704429487 |
| cellular\_calcium\_ion\_homeostasis | 0.109383766727661 | 0.0720818760422794 | 0.184473704429487 |
| toll-like\_receptor\_9\_signaling\_pathway | 0.107122157728389 | 0.315364056557002 | 0.0265118887830299 |
| toll-like\_receptor\_2\_signaling\_pathway | 0.107122157728389 | 0.315364056557002 | 0.0265118887830299 |
| Ras\_protein\_signal\_transduction | 0.107122157728389 | 0.315364056557002 | 0.0265118887830299 |
| negative\_regulation\_of\_cell\_migration | 0.0228774354117492 | 0.070549345997889 | 0.0265118887830299 |
| metallopeptidase\_activity | 0.0228774354117492 | 0.0137238830109637 | 0.182650075953045 |
| actin\_filament\_binding | 0.107122157728389 | 0.315364056557002 | 0.0265118887830299 |
| mitochondrial\_membrane | 0.022614719612846 | 0.013346340827094 | 0.180629926503872 |
| collagen\_catabolic\_process | 0.000199028211441313 | 0.31298630747656 | 1.2155468818029e-06 |
| toll-like\_receptor\_TLR6:TLR2\_signaling\_pathway | 0.102952439315454 | 0.309924719942446 | 0.0254030635409161 |
| toll-like\_receptor\_TLR1:TLR2\_signaling\_pathway | 0.102952439315454 | 0.309924719942446 | 0.0254030635409161 |
| protein\_stabilization | 0.102952439315454 | 0.0671907013046569 | 0.178511011199242 |
| positive\_regulation\_of\_ubiquitin-protein\_ligase\_activity\_involved\_in\_mitotic\_cell\_cycle | 0.102952439315454 | 0.309924719942446 | 0.0254030635409161 |
| lysosomal\_lumen | 0.00434155524692406 | 0.0129048466918583 | 0.0254030635409161 |
| cellular\_response\_to\_lipopolysaccharide | 0.102952439315454 | 0.309924719942446 | 0.0254030635409161 |
| pattern\_specification\_process | 0.101045746494658 | 0.307476588314657 | 0.0249366414982227 |
| nucleotide-excision\_repair | 0.101045746494658 | 0.307476588314657 | 0.0249366414982227 |
| regulation\_of\_cell\_growth | 0.0204529094717856 | 0.0119854113391728 | 0.174070624764913 |
| positive\_regulation\_of\_cell\_growth | 0.0989125860090769 | 0.0656271758185004 | 0.174070624764913 |
| extracellular\_matrix | 0.0204529094717856 | 0.0119854113391728 | 0.174070624764913 |
| response\_to\_toxin | 0.0201310323544729 | 0.0642126640193429 | 0.0236772931756473 |
| response\_to\_nutrient | 0.0966776660941545 | 0.300564168333355 | 0.0236772931756473 |
| positive\_regulation\_of\_peptidyl-tyrosine\_phosphorylation | 0.0201310323544729 | 0.0642126640193429 | 0.0236772931756473 |
| neural\_tube\_closure | 0.0201310323544729 | 0.0642126640193429 | 0.0236772931756473 |
| defense\_response | 0.0966776660941545 | 0.300564168333355 | 0.0236772931756473 |
| cartilage\_development | 0.0201310323544729 | 0.300564168333355 | 0.00301502274570466 |
| secretory\_granule | 0.0194625314285973 | 0.0113782989130067 | 0.170444867396209 |
| negative\_regulation\_of\_gene\_expression | 0.0946618957274452 | 0.0628025824914435 | 0.170444867396209 |
| extracellular\_vesicular\_exosome | 0.0946618957274452 | 0.298011265562405 | 0.023608304798247 |
| cholesterol\_metabolic\_process | 0.0946618957274452 | 0.0628025824914435 | 0.170444867396209 |
| cellular\_iron\_ion\_homeostasis | 0.0946618957274452 | 0.0628025824914435 | 0.170444867396209 |
| extracellular\_matrix\_structural\_constituent | 0.00363332341711948 | 0.0110782436810008 | 0.0234442582418545 |
| type\_I\_interferon-mediated\_signaling\_pathway | 0.0903341475036955 | 0.0605897900726602 | 0.0228107377550087 |
| toll-like\_receptor\_5\_signaling\_pathway | 0.0903341475036955 | 0.290627627850994 | 0.0228107377550087 |
| toll-like\_receptor\_10\_signaling\_pathway | 0.0903341475036955 | 0.290627627850994 | 0.0228107377550087 |
| regulation\_of\_sequence-specific\_DNA\_binding\_transcription\_factor\_activity | 0.0903341475036955 | 0.290627627850994 | 0.0228107377550087 |
| oxidoreductase\_activity\_\_acting\_on\_single\_donors\_with\_incorporation\_of\_molecular\_oxygen\_\_incorporation\_of\_two\_atoms\_of\_oxygen | 0.0903341475036955 | 0.0605897900726602 | 0.166418036859805 |
| negative\_regulation\_of\_ubiquitin-protein\_ligase\_activity\_involved\_in\_mitotic\_cell\_cycle | 0.0903341475036955 | 0.290627627850994 | 0.0228107377550087 |
| MAPK\_cascade | 0.0903341475036955 | 0.290627627850994 | 0.0228107377550087 |
| interferon-gamma-mediated\_signaling\_pathway | 0.0181557792073582 | 0.0605897900726602 | 0.00274381585953723 |
| integral\_to\_endoplasmic\_reticulum\_membrane | 0.0903341475036955 | 0.0605897900726602 | 0.166418036859805 |
| condensed\_chromosome\_kinetochore | 0.00344152249892456 | 0.290627627850994 | 0.000288091180134057 |
| chromatin | 0.0903341475036955 | 0.0605897900726602 | 0.166418036859805 |
| cell-cell\_junction\_organization | 0.0181557792073582 | 0.0605897900726602 | 0.0228107377550087 |
| wound\_healing | 0.0888289088358695 | 0.288886412810424 | 0.0224655694460054 |
| positive\_regulation\_of\_MAPK\_cascade | 0.0888289088358695 | 0.059578205681 | 0.164714503214897 |
| peptidyl-serine\_phosphorylation | 0.0888289088358695 | 0.288886412810424 | 0.0224655694460054 |
| palate\_development | 0.0177517370442636 | 0.288886412810424 | 0.00263310146310659 |
| chromosome\_segregation | 0.0888289088358695 | 0.288886412810424 | 0.0224655694460054 |
| single-stranded\_DNA\_binding | 0.0868207563415589 | 0.286182632971734 | 0.0219575680556821 |
| sarcolemma | 0.0868207563415589 | 0.0579758771130325 | 0.162498900885701 |
| response\_to\_estradiol\_stimulus | 0.0846241783568861 | 0.282832041936179 | 0.021372498013801 |
| negative\_regulation\_of\_cysteine-type\_endopeptidase\_activity\_involved\_in\_apoptotic\_process | 0.0165195849882656 | 0.056387967789279 | 0.021372498013801 |
| odontogenesis\_of\_dentin-containing\_tooth | 0.0824401903521348 | 0.0556537559364115 | 0.158036597203891 |
| glucose\_transport | 0.0824401903521348 | 0.279150471723561 | 0.0207923021381329 |
| DNA\_binding\_\_bending | 0.0824401903521348 | 0.279150471723561 | 0.0207923021381329 |
| regulation\_of\_blood\_pressure | 0.0152291126406607 | 0.0542579222497554 | 0.0203948830732625 |
| negative\_regulation\_of\_NF-kappaB\_transcription\_factor\_activity | 0.0804525634912122 | 0.0542579222497554 | 0.156105168411169 |
| microtubule\_cytoskeleton\_organization | 0.0804525634912122 | 0.275739056565802 | 0.0203948830732625 |
| cellular\_response\_to\_mechanical\_stimulus | 0.0762305531360116 | 0.268240939418724 | 0.0198201767482153 |
| positive\_regulation\_of\_osteoblast\_differentiation | 0.0741807158293513 | 0.0497569970718571 | 0.149440535859605 |
| drug\_binding | 0.0741807158293513 | 0.0497569970718571 | 0.149440535859605 |
| cytoplasmic\_mRNA\_processing\_body | 0.0741807158293513 | 0.2647380861253 | 0.0192043643172084 |
| steroid\_metabolic\_process | 0.0129763891789149 | 0.00769755349131371 | 0.147294828670372 |
| ruffle\_membrane | 0.0721418149047977 | 0.0496092325493203 | 0.147294828670372 |
| retinoid\_metabolic\_process | 0.0721418149047977 | 0.0496092325493203 | 0.147294828670372 |
| recycling\_endosome | 0.0721418149047977 | 0.260920396812515 | 0.0186686006056541 |
| O-glycan\_processing | 0.000440511814719415 | 0.000158788091475497 | 0.147294828670372 |
| lipid\_transport | 0.0721418149047977 | 0.0496092325493203 | 0.147294828670372 |
| intrinsic\_apoptotic\_signaling\_pathway | 0.0721418149047977 | 0.260920396812515 | 0.0186686006056541 |
| inner\_ear\_morphogenesis | 0.0721418149047977 | 0.260920396812515 | 0.0186686006056541 |
| cysteine-type\_endopeptidase\_activity | 0.0129763891789149 | 0.00769755349131371 | 0.147294828670372 |
| cholesterol\_homeostasis | 0.0721418149047977 | 0.0496092325493203 | 0.147294828670372 |
| activation\_of\_phospholipase\_C\_activity | 0.0721418149047977 | 0.260920396812515 | 0.0186686006056541 |
| positive\_regulation\_of\_neuron\_projection\_development | 0.0125624080579598 | 0.257912018832443 | 0.00187893853374998 |
| keratinocyte\_differentiation | 0.0125624080579598 | 0.257912018832443 | 0.00187893853374998 |
| digestion | 0.00232324639784093 | 0.00761642944803155 | 0.0181722564865417 |
| chromosome\_\_centromeric\_region | 0.0705234874696912 | 0.257912018832443 | 0.0181722564865417 |
| cellular\_response\_to\_insulin\_stimulus | 0.0705234874696912 | 0.257912018832443 | 0.0181722564865417 |
| stress-activated\_MAPK\_cascade | 0.0684177000787213 | 0.254859225448025 | 0.0176433561504662 |
| response\_to\_estrogen\_stimulus | 0.0684177000787213 | 0.0466909560750767 | 0.143545960953345 |
| regulation\_of\_cyclin-dependent\_protein\_kinase\_activity | 0.0684177000787213 | 0.0466909560750767 | 0.143545960953345 |
| positive\_regulation\_of\_epithelial\_cell\_proliferation | 0.0684177000787213 | 0.0466909560750767 | 0.143545960953345 |
| positive\_regulation\_of\_canonical\_Wnt\_receptor\_signaling\_pathway | 0.00217402875472453 | 0.0466909560750767 | 0.00179686876915908 |
| osteoblast\_differentiation | 0.0684177000787213 | 0.0466909560750767 | 0.143545960953345 |
| negative\_regulation\_of\_inflammatory\_response | 0.0684177000787213 | 0.0466909560750767 | 0.143545960953345 |
| calcium\_channel\_activity | 0.0119790743059199 | 0.00725112621784316 | 0.143545960953345 |
| telomere\_maintenance | 0.0667988007908954 | 0.250938605227607 | 0.0171184852722171 |
| response\_to\_stimulus | 0.00203147225223715 | 0.00697533598853363 | 0.0171184852722171 |
| regulation\_of\_cell\_migration | 0.0117265851408507 | 0.250938605227607 | 0.00179293589730835 |
| positive\_regulation\_of\_JNK\_cascade | 0.0667988007908954 | 0.250938605227607 | 0.0171184852722171 |
| positive\_regulation\_of\_endothelial\_cell\_proliferation | 0.0667988007908954 | 0.250938605227607 | 0.0171184852722171 |
| negative\_regulation\_of\_angiogenesis | 0.0117265851408507 | 0.00697533598853363 | 0.0171184852722171 |
| fat\_cell\_differentiation | 0.0667988007908954 | 0.0456607557731409 | 0.141931763363668 |
| endoplasmic\_reticulum-Golgi\_intermediate\_compartment | 0.0117265851408507 | 0.00697533598853363 | 0.141931763363668 |
| cytoskeletal\_protein\_binding | 0.0667988007908954 | 0.0456607557731409 | 0.141931763363668 |
| cellular\_response\_to\_retinoic\_acid | 0.0667988007908954 | 0.250938605227607 | 0.0171184852722171 |
| positive\_regulation\_of\_peptidyl-serine\_phosphorylation | 0.0112006384819317 | 0.248073115714973 | 0.0017238010297691 |
| peroxisomal\_membrane | 0.0112006384819317 | 0.0442223918322977 | 0.0166979583284687 |
| cytosolic\_large\_ribosomal\_subunit | 0.0650992681055029 | 0.248073115714973 | 0.0166979583284687 |
| cell\_morphogenesis | 0.0650992681055029 | 0.248073115714973 | 0.0166979583284687 |
| cell\_growth | 0.0650992681055029 | 0.0442223918322977 | 0.140145532607723 |
| response\_to\_cytokine\_stimulus | 0.00196317351177434 | 0.000920638434609218 | 0.0166979583284687 |
| protein\_binding\_\_bridging | 0.0631721831854453 | 0.244618571482334 | 0.0166979583284687 |
| negative\_regulation\_of\_phosphatase\_activity | 0.0631721831854453 | 0.244618571482334 | 0.0166979583284687 |
| heart\_looping | 0.0631721831854453 | 0.244618571482334 | 0.0166979583284687 |
| kinetochore | 0.0614032256552675 | 0.241388523403632 | 0.0166477284060776 |
| ion\_channel\_activity | 0.0614032256552675 | 0.0423963450263797 | 0.135502658220476 |
| stem\_cell\_maintenance | 0.0594220065109267 | 0.0408926196808138 | 0.133137301785025 |
| mitochondrial\_intermembrane\_space | 0.0594220065109267 | 0.0408926196808138 | 0.133137301785025 |
| double-strand\_break\_repair\_via\_homologous\_recombination | 0.0594220065109267 | 0.238639985775108 | 0.0160420461002586 |
| transcription\_cofactor\_activity | 0.0575265293837464 | 0.0397079856419612 | 0.130942323984681 |
| scavenger\_receptor\_activity | 0.0575265293837464 | 0.234784833851981 | 0.0155127787819655 |
| negative\_regulation\_of\_neuron\_differentiation | 0.0575265293837464 | 0.234784833851981 | 0.0155127787819655 |
| chondroitin\_sulfate\_metabolic\_process | 0.0575265293837464 | 0.234784833851981 | 0.0155127787819655 |
| chloride\_channel\_complex | 0.0575265293837464 | 0.0397079856419612 | 0.130942323984681 |
| chemokine\_activity | 0.0575265293837464 | 0.0397079856419612 | 0.130942323984681 |
| activation\_of\_MAPKK\_activity | 0.0575265293837464 | 0.234784833851981 | 0.0155127787819655 |
| transcription-coupled\_nucleotide-excision\_repair | 0.0559853894877794 | 0.231409741004614 | 0.0150544953831872 |
| sensory\_perception\_of\_pain | 0.0559853894877794 | 0.231409741004614 | 0.0150544953831872 |
| response\_to\_retinoic\_acid | 0.00902936020332099 | 0.00537402012864733 | 0.128908555540131 |
| glycolysis | 0.0559853894877794 | 0.0383799432407799 | 0.128908555540131 |
| embryonic\_digit\_morphogenesis | 0.0559853894877794 | 0.231409741004614 | 0.0150544953831872 |
| cell\_fate\_commitment | 0.0559853894877794 | 0.231409741004614 | 0.0150544953831872 |
| termination\_of\_RNA\_polymerase\_II\_transcription | 0.0543070024145507 | 0.228241277058419 | 0.0145980181408409 |
| Rho\_protein\_signal\_transduction | 0.0543070024145507 | 0.228241277058419 | 0.0145980181408409 |
| positive\_regulation\_of\_inflammatory\_response | 0.00868557002931465 | 0.228241277058419 | 0.00137916014007048 |
| chloride\_channel\_activity | 0.00140576853311462 | 0.000711046315092279 | 0.126584169106876 |
| cellular\_response\_to\_tumor\_necrosis\_factor | 0.0543070024145507 | 0.0369215183775715 | 0.126584169106876 |
| negative\_regulation\_of\_epithelial\_cell\_proliferation | 0.00140576853311462 | 0.00478412095789432 | 0.0140483355019588 |
| collagen\_binding | 0.0525008747561708 | 0.224763012597237 | 0.0140483355019588 |
| transferase\_activity\_\_transferring\_acyl\_groups | 0.0504524341210587 | 0.035499744572273 | 0.12201558872008 |
| positive\_regulation\_of\_MAP\_kinase\_activity | 0.0504524341210587 | 0.220732214581565 | 0.0135059703289925 |
| cellular\_amino\_acid\_metabolic\_process | 0.0504524341210587 | 0.220732214581565 | 0.0135059703289925 |
| positive\_regulation\_of\_neuron\_apoptotic\_process | 0.0485542495074005 | 0.216909383464194 | 0.0130300447775725 |
| glycosphingolipid\_metabolic\_process | 0.0485542495074005 | 0.0340505031511583 | 0.119766939260435 |
| extrinsic\_to\_membrane | 0.00733186198175003 | 0.00443962245032569 | 0.119766939260435 |
| excretion | 0.000210194600115693 | 4.90905391688342e-05 | 0.119766939260435 |
| response\_to\_UV | 0.0466178720020338 | 0.213283477060344 | 0.0125655770024194 |
| response\_to\_organic\_cyclic\_compound | 0.0012160872803861 | 0.0041907646433162 | 0.0125655770024194 |
| positive\_regulation\_of\_fibroblast\_proliferation | 0.0466178720020338 | 0.213283477060344 | 0.0125655770024194 |
| organ\_regeneration | 0.0466178720020338 | 0.213283477060344 | 0.0125655770024194 |
| negative\_regulation\_of\_cell\_cycle | 0.0466178720020338 | 0.213283477060344 | 0.0125655770024194 |
| mRNA\_3-end\_processing | 0.0466178720020338 | 0.213283477060344 | 0.0125655770024194 |
| cellular\_response\_to\_growth\_factor\_stimulus | 0.00686876480904816 | 0.0041907646433162 | 0.117622660896563 |
| cellular\_protein\_localization | 0.00686876480904816 | 0.213283477060344 | 0.00117512921584362 |
| branching\_involved\_in\_ureteric\_bud\_morphogenesis | 0.0466178720020338 | 0.213283477060344 | 0.0125655770024194 |
| triglyceride\_biosynthetic\_process | 0.0449904436366656 | 0.210327588441197 | 0.0125655770024194 |
| regulation\_of\_cell\_adhesion | 0.0449904436366656 | 0.0312848610504021 | 0.115083792883116 |
| positive\_regulation\_of\_smooth\_muscle\_cell\_proliferation | 0.00670396597431235 | 0.0041376725283185 | 0.0125655770024194 |
| negative\_regulation\_of\_Wnt\_receptor\_signaling\_pathway | 0.0449904436366656 | 0.0312848610504021 | 0.115083792883116 |
| blood\_circulation | 0.00670396597431235 | 0.0041376725283185 | 0.0125655770024194 |
| response\_to\_peptide\_hormone\_stimulus | 0.0432046535335189 | 0.0299642354068707 | 0.0121664106203322 |
| ovarian\_follicle\_development | 0.0432046535335189 | 0.205870076017113 | 0.0121664106203322 |
| endocrine\_pancreas\_development | 0.0432046535335189 | 0.0299642354068707 | 0.112773892888365 |
| ureteric\_bud\_development | 0.0414377339352987 | 0.0294946940892483 | 0.110205731090442 |
| somitogenesis | 0.0414377339352987 | 0.201614207437133 | 0.0116334535044566 |
| peripheral\_nervous\_system\_development | 0.0414377339352987 | 0.201614207437133 | 0.0116334535044566 |
| inner\_ear\_development | 0.0414377339352987 | 0.201614207437133 | 0.0116334535044566 |
| embryonic\_skeletal\_system\_morphogenesis | 0.0414377339352987 | 0.201614207437133 | 0.0116334535044566 |
| collagen\_fibril\_organization | 0.0414377339352987 | 0.201614207437133 | 0.0116334535044566 |
| response\_to\_cAMP | 0.0398959659207918 | 0.0284260891669219 | 0.0111920060081932 |
| positive\_regulation\_of\_protein\_catabolic\_process | 0.0398959659207918 | 0.198235616105857 | 0.0111920060081932 |
| glutathione\_metabolic\_process | 0.0398959659207918 | 0.0284260891669219 | 0.107856873605096 |
| erythrocyte\_differentiation | 0.0398959659207918 | 0.198235616105857 | 0.0111920060081932 |
| epithelial\_cell\_differentiation | 0.00569634568180426 | 0.00354439039819279 | 0.107856873605096 |
| de\_novo\_posttranslational\_protein\_folding | 0.0398959659207918 | 0.0284260891669219 | 0.107856873605096 |
| brush\_border\_membrane | 0.000894897978066401 | 0.000433363745506403 | 0.107856873605096 |
| amino\_acid\_transport | 0.0398959659207918 | 0.198235616105857 | 0.0111920060081932 |
| water\_transport | 0.0384113383509313 | 0.0272958035951108 | 0.105714202195353 |
| sulfotransferase\_activity | 0.00529739058532867 | 0.0272958035951108 | 0.0107266408881049 |
| RNA\_polymerase\_II\_core\_promoter\_proximal\_region\_sequence-specific\_DNA\_binding | 0.0384113383509313 | 0.0272958035951108 | 0.0107266408881049 |
| positive\_regulation\_of\_interleukin-6\_production | 0.00529739058532867 | 0.0272958035951108 | 0.0107266408881049 |
| positive\_regulation\_of\_DNA\_replication | 0.0384113383509313 | 0.194560616145742 | 0.0107266408881049 |
| neuron\_development | 0.0384113383509313 | 0.194560616145742 | 0.0107266408881049 |
| mitotic\_nuclear\_envelope\_disassembly | 0.0384113383509313 | 0.194560616145742 | 0.0107266408881049 |
| glycosaminoglycan\_biosynthetic\_process | 0.00529739058532867 | 0.0272958035951108 | 0.0107266408881049 |
| defense\_response\_to\_Gram-positive\_bacterium | 0.00529739058532867 | 0.0272958035951108 | 0.0107266408881049 |
| aspartic-type\_endopeptidase\_activity | 0.0384113383509313 | 0.0272958035951108 | 0.105714202195353 |
| acute-phase\_response | 0.0384113383509313 | 0.0272958035951108 | 0.105714202195353 |
| placenta\_development | 0.00523218016722666 | 0.026345204032721 | 0.0103715237619487 |
| bile\_acid\_metabolic\_process | 0.0369806272201755 | 0.190373141265223 | 0.0103715237619487 |
| trans-Golgi\_network\_membrane | 0.035221648787957 | 0.185915670626796 | 0.00986156911481812 |
| somatic\_stem\_cell\_maintenance | 0.0048322021289966 | 0.025006952803094 | 0.00986156911481812 |
| protein\_kinase\_C-activating\_G-protein\_coupled\_receptor\_signaling\_pathway | 0.035221648787957 | 0.185915670626796 | 0.00986156911481812 |
| negative\_regulation\_of\_BMP\_signaling\_pathway | 0.0048322021289966 | 0.025006952803094 | 0.00986156911481812 |
| myelination | 0.035221648787957 | 0.025006952803094 | 0.10067802807216 |
| metabolic\_process | 0.035221648787957 | 0.025006952803094 | 0.10067802807216 |
| hair\_follicle\_development | 0.035221648787957 | 0.185915670626796 | 0.00986156911481812 |
| frizzled\_binding | 0.0048322021289966 | 0.025006952803094 | 0.00986156911481812 |
| cellular\_response\_to\_amino\_acid\_stimulus | 0.035221648787957 | 0.185915670626796 | 0.00986156911481812 |
| respiratory\_gaseous\_exchange | 0.00450563597812728 | 0.181844514201223 | 0.000732242206204171 |
| protein\_localization\_in\_plasma\_membrane | 0.0337159474110229 | 0.0239061669291247 | 0.0982402135035233 |
| positive\_regulation\_of\_interferon-gamma\_production | 0.0337159474110229 | 0.181844514201223 | 0.00948403056686321 |
| positive\_regulation\_of\_cysteine-type\_endopeptidase\_activity\_involved\_in\_apoptotic\_process | 0.00450563597812728 | 0.181844514201223 | 0.000732242206204171 |
| patterning\_of\_blood\_vessels | 0.000764739465746739 | 0.0239061669291247 | 0.000732242206204171 |
| negative\_regulation\_of\_osteoblast\_differentiation | 0.0337159474110229 | 0.0239061669291247 | 0.0982402135035233 |
| microvillus | 0.0337159474110229 | 0.0239061669291247 | 0.0982402135035233 |
| lateral\_plasma\_membrane | 0.0337159474110229 | 0.181844514201223 | 0.00948403056686321 |
| hydrolase\_activity\_\_hydrolyzing\_O-glycosyl\_compounds | 0.0337159474110229 | 0.0239061669291247 | 0.0982402135035233 |
| clathrin-coated\_vesicle | 0.0337159474110229 | 0.0239061669291247 | 0.0982402135035233 |
| ATPase\_activity\_\_coupled\_to\_transmembrane\_movement\_of\_substances | 0.0337159474110229 | 0.0239061669291247 | 0.0982402135035233 |
| aminopeptidase\_activity | 0.0337159474110229 | 0.0239061669291247 | 0.0982402135035233 |
| triglyceride\_metabolic\_process | 0.0323088140220208 | 0.17813601684484 | 0.00901138567267291 |
| transport\_vesicle\_membrane | 0.00442058905540836 | 0.0025968350470555 | 0.0964117790565486 |
| reciprocal\_meiotic\_recombination | 0.0323088140220208 | 0.0230214492436921 | 0.0964117790565486 |
| positive\_regulation\_of\_tumor\_necrosis\_factor\_production | 0.0323088140220208 | 0.0230214492436921 | 0.0964117790565486 |
| positive\_regulation\_of\_endothelial\_cell\_migration | 0.0323088140220208 | 0.17813601684484 | 0.00901138567267291 |
| negative\_regulation\_of\_viral\_genome\_replication | 0.0323088140220208 | 0.0230214492436921 | 0.0964117790565486 |
| keratan\_sulfate\_metabolic\_process | 6.22798360502092e-05 | 1.45363734728352e-05 | 0.0964117790565486 |
| germ\_cell\_development | 0.0323088140220208 | 0.0230214492436921 | 0.0964117790565486 |
| cellular\_response\_to\_cAMP | 0.00442058905540836 | 0.0025968350470555 | 0.0964117790565486 |
| cellular\_response\_to\_calcium\_ion | 0.00442058905540836 | 0.0230214492436921 | 0.00901138567267291 |
| activation\_of\_JUN\_kinase\_activity | 0.0323088140220208 | 0.17813601684484 | 0.00901138567267291 |
| tight\_junction\_assembly | 0.0308649094643973 | 0.022085088918651 | 0.0943301798354212 |
| rough\_endoplasmic\_reticulum | 0.0308649094643973 | 0.022085088918651 | 0.0943301798354212 |
| RNA\_polymerase\_II\_core\_promoter\_proximal\_region\_sequence-specific\_DNA\_binding\_transcription\_factor\_activity\_involved\_in\_negative\_regulation\_of\_transcription | 0.0308649094643973 | 0.174143264607277 | 0.00859244601900673 |
| protein\_deubiquitination | 0.0308649094643973 | 0.022085088918651 | 0.0943301798354212 |
| positive\_regulation\_of\_mesenchymal\_cell\_proliferation | 0.00408302592769542 | 0.022085088918651 | 0.000641287994875923 |
| neuron\_projection\_morphogenesis | 0.0308649094643973 | 0.022085088918651 | 0.0943301798354212 |
| negative\_regulation\_of\_type\_I\_interferon\_production | 0.00408302592769542 | 0.00254603755891334 | 0.0943301798354212 |
| negative\_regulation\_of\_fat\_cell\_differentiation | 0.0308649094643973 | 0.174143264607277 | 0.00859244601900673 |
| mitotic\_cell\_cycle\_spindle\_assembly\_checkpoint | 0.0308649094643973 | 0.174143264607277 | 0.00859244601900673 |
| growth | 0.0308649094643973 | 0.174143264607277 | 0.00859244601900673 |
| fertilization | 0.0308649094643973 | 0.022085088918651 | 0.0943301798354212 |
| dendrite\_development | 0.0308649094643973 | 0.022085088918651 | 0.0943301798354212 |
| cyclin-dependent\_protein\_kinase\_activity | 0.0308649094643973 | 0.174143264607277 | 0.00859244601900673 |
| chloride\_transport | 0.000610726829028144 | 0.00034352126573316 | 0.0943301798354212 |
| cellular\_response\_to\_transforming\_growth\_factor\_beta\_stimulus | 0.00408302592769542 | 0.022085088918651 | 0.00859244601900673 |
| RNA\_polymerase\_II\_transcription\_factor\_binding | 0.029513262997273 | 0.022085088918651 | 0.0924155050015092 |
| phosphatidylinositol\_phospholipase\_C\_activity | 0.029513262997273 | 0.170445594788505 | 0.00822262466457099 |
| negative\_regulation\_of\_cell\_adhesion | 0.029513262997273 | 0.170445594788505 | 0.00822262466457099 |
| epithelial\_to\_mesenchymal\_transition | 0.029513262997273 | 0.170445594788505 | 0.00822262466457099 |
| endopeptidase\_activity | 0.029513262997273 | 0.170445594788505 | 0.00822262466457099 |
| DNA\_strand\_elongation\_involved\_in\_DNA\_replication | 0.029513262997273 | 0.170445594788505 | 0.00822262466457099 |
| cation-transporting\_ATPase\_activity | 0.029513262997273 | 0.170445594788505 | 0.00822262466457099 |
| bicarbonate\_transport | 3.80689023326619e-08 | 3.57753723980458e-09 | 0.0924155050015092 |
| spindle\_microtubule | 0.0280868728705725 | 0.166753690444145 | 0.0078546738578739 |
| response\_to\_cold | 0.0280868728705725 | 0.0210754897097915 | 0.0899459653694866 |
| cellular\_response\_to\_interleukin-1 | 0.0280868728705725 | 0.0210754897097915 | 0.0899459653694866 |
| cellular\_response\_to\_hydrogen\_peroxide | 0.0280868728705725 | 0.166753690444145 | 0.0078546738578739 |
| brown\_fat\_cell\_differentiation | 0.0280868728705725 | 0.0210754897097915 | 0.0899459653694866 |
| reactive\_oxygen\_species\_metabolic\_process | 0.0266340767827767 | 0.162330968508269 | 0.00773538987579499 |
| positive\_regulation\_of\_stress\_fiber\_assembly | 0.0266340767827767 | 0.0199243740366313 | 0.0873482603877234 |
| one-carbon\_metabolic\_process | 3.31587425242005e-05 | 7.77926277850103e-06 | 0.0873482603877234 |
| odontogenesis | 0.0266340767827767 | 0.162330968508269 | 0.00773538987579499 |
| activation\_of\_adenylate\_cyclase\_activity | 0.0266340767827767 | 0.162330968508269 | 0.00773538987579499 |
| Wnt-protein\_binding | 0.0251610389437747 | 0.0186969747319295 | 0.08472572723775 |
| T-tubule | 0.0251610389437747 | 0.157647761621443 | 0.00731603414798099 |
| steroid\_biosynthetic\_process | 0.00318729002303362 | 0.00202695040078217 | 0.08472572723775 |
| sarcoplasmic\_reticulum | 0.0251610389437747 | 0.0186969747319295 | 0.08472572723775 |
| positive\_regulation\_of\_fat\_cell\_differentiation | 0.0251610389437747 | 0.0186969747319295 | 0.08472572723775 |
| lipopolysaccharide-mediated\_signaling\_pathway | 0.0251610389437747 | 0.0186969747319295 | 0.00731603414798099 |
| keratan\_sulfate\_biosynthetic\_process | 2.87678442125726e-05 | 6.75408469696703e-06 | 0.08472572723775 |
| hair\_follicle\_morphogenesis | 0.0251610389437747 | 0.157647761621443 | 0.00731603414798099 |
| gonad\_development | 0.0251610389437747 | 0.0186969747319295 | 0.08472572723775 |
| apoptotic\_DNA\_fragmentation | 0.00318729002303362 | 0.0186969747319295 | 0.00731603414798099 |
| voltage-gated\_ion\_channel\_activity | 0.0238433345652865 | 0.0177235809241748 | 0.0824528804039839 |
| ventricular\_cardiac\_muscle\_tissue\_morphogenesis | 0.0238433345652865 | 0.152718385111042 | 0.00688632217957504 |
| spleen\_development | 0.0238433345652865 | 0.0177235809241748 | 0.0824528804039839 |
| RNA\_polymerase\_II\_core\_promoter\_sequence-specific\_DNA\_binding | 0.0238433345652865 | 0.0177235809241748 | 0.00688632217957504 |
| regulation\_of\_type\_I\_interferon-mediated\_signaling\_pathway | 0.0238433345652865 | 0.0177235809241748 | 0.00688632217957504 |
| positive\_regulation\_of\_tyrosine\_phosphorylation\_of\_Stat3\_protein | 0.0238433345652865 | 0.0177235809241748 | 0.0824528804039839 |
| negative\_regulation\_of\_I-kappaB\_kinase/NF-kappaB\_cascade | 0.0238433345652865 | 0.0177235809241748 | 0.00688632217957504 |
| lamellipodium\_assembly | 0.0238433345652865 | 0.152718385111042 | 0.00688632217957504 |
| embryonic\_skeletal\_system\_development | 0.0238433345652865 | 0.152718385111042 | 0.00688632217957504 |
| developmental\_growth | 0.0238433345652865 | 0.152718385111042 | 0.00688632217957504 |
| tumor\_necrosis\_factor\_receptor\_binding | 0.022614719612846 | 0.0167584126512601 | 0.00652399359252917 |
| tumor\_necrosis\_factor-mediated\_signaling\_pathway | 0.022614719612846 | 0.0167584126512601 | 0.00652399359252917 |
| telomere\_maintenance\_via\_recombination | 0.022614719612846 | 0.148658971383702 | 0.00652399359252917 |
| sensory\_perception\_of\_taste | 0.00266422514859511 | 0.00163097401049154 | 0.0804136969910517 |
| response\_to\_organic\_substance | 0.022614719612846 | 0.148658971383702 | 0.00652399359252917 |
| response\_to\_axon\_injury | 0.022614719612846 | 0.148658971383702 | 0.00652399359252917 |
| response\_to\_activity | 0.022614719612846 | 0.148658971383702 | 0.00652399359252917 |
| positive\_regulation\_of\_pathway-restricted\_SMAD\_protein\_phosphorylation | 0.022614719612846 | 0.148658971383702 | 0.00652399359252917 |
| positive\_regulation\_of\_mitotic\_cell\_cycle | 0.00266422514859511 | 0.0167584126512601 | 0.00652399359252917 |
| positive\_regulation\_of\_intrinsic\_apoptotic\_signaling\_pathway | 0.022614719612846 | 0.148658971383702 | 0.00652399359252917 |
| phospholipid\_biosynthetic\_process | 0.022614719612846 | 0.148658971383702 | 0.00652399359252917 |
| negative\_regulation\_of\_smooth\_muscle\_cell\_proliferation | 0.00266422514859511 | 0.00163097401049154 | 0.0804136969910517 |
| negative\_regulation\_of\_ERK1\_and\_ERK2\_cascade | 0.022614719612846 | 0.0167584126512601 | 0.0804136969910517 |
| lens\_development\_in\_camera-type\_eye | 0.022614719612846 | 0.148658971383702 | 0.00652399359252917 |
| JAK-STAT\_cascade | 0.022614719612846 | 0.0167584126512601 | 0.00652399359252917 |
| extrinsic\_apoptotic\_signaling\_pathway | 0.00266422514859511 | 0.0167584126512601 | 0.00652399359252917 |
| estrogen\_receptor\_binding | 0.022614719612846 | 0.148658971383702 | 0.00652399359252917 |
| cytokine\_receptor\_activity | 0.022614719612846 | 0.0167584126512601 | 0.0804136969910517 |
| cellular\_response\_to\_starvation | 0.022614719612846 | 0.0167584126512601 | 0.0804136969910517 |
| cell\_aging | 0.00266422514859511 | 0.0167584126512601 | 0.00652399359252917 |
| calcium-dependent\_cell-cell\_adhesion | 0.00266422514859511 | 0.0167584126512601 | 0.00652399359252917 |
| response\_to\_zinc\_ion | 0.0215186043127415 | 0.0159294382422371 | 0.0785028300204395 |
| release\_of\_cytochrome\_c\_from\_mitochondria | 0.0215186043127415 | 0.144867620368305 | 0.0063441236640472 |
| peptide\_hormone\_binding | 0.0215186043127415 | 0.144867620368305 | 0.0063441236640472 |
| negative\_regulation\_of\_endothelial\_cell\_proliferation | 0.0215186043127415 | 0.0159294382422371 | 0.0063441236640472 |
| mitotic\_cell\_cycle\_checkpoint | 0.00257376048896922 | 0.0159294382422371 | 0.0063441236640472 |
| E-box\_binding | 0.0215186043127415 | 0.144867620368305 | 0.0063441236640472 |
| chondroitin\_sulfate\_biosynthetic\_process | 0.0215186043127415 | 0.144867620368305 | 0.0063441236640472 |
| positive\_regulation\_of\_Wnt\_receptor\_signaling\_pathway | 0.020134293229218 | 0.0148161564195688 | 0.0759884121844859 |
| positive\_regulation\_of\_protein\_insertion\_into\_mitochondrial\_membrane\_involved\_in\_apoptotic\_signaling\_pathway | 0.020134293229218 | 0.140252475346672 | 0.00602610432319336 |
| phosphatidylcholine\_biosynthetic\_process | 0.020134293229218 | 0.0148161564195688 | 0.0759884121844859 |
| oxidoreductase\_activity\_\_acting\_on\_paired\_donors\_\_with\_incorporation\_or\_reduction\_of\_molecular\_oxygen\_\_2-oxoglutarate\_as\_one\_donor\_\_and\_incorporation\_of\_one\_atom\_each\_of\_oxygen\_into\_both\_donors | 0.020134293229218 | 0.0148161564195688 | 0.0759884121844859 |
| negative\_regulation\_of\_insulin\_secretion | 0.020134293229218 | 0.0148161564195688 | 0.0759884121844859 |
| negative\_regulation\_of\_fibroblast\_proliferation | 0.000366589064722387 | 0.0148161564195688 | 0.000388417844262922 |
| negative\_regulation\_of\_blood\_pressure | 0.020134293229218 | 0.0148161564195688 | 0.0759884121844859 |
| mRNA\_polyadenylation | 0.020134293229218 | 0.140252475346672 | 0.00602610432319336 |
| metallocarboxypeptidase\_activity | 0.020134293229218 | 0.0148161564195688 | 0.0759884121844859 |
| JAK-STAT\_cascade\_involved\_in\_growth\_hormone\_signaling\_pathway | 0.020134293229218 | 0.0148161564195688 | 0.00602610432319336 |
| hippo\_signaling\_cascade | 0.020134293229218 | 0.140252475346672 | 0.00602610432319336 |
| glutathione\_transferase\_activity | 0.020134293229218 | 0.0148161564195688 | 0.0759884121844859 |
| brush\_border | 0.020134293229218 | 0.0148161564195688 | 0.0759884121844859 |
| vasculature\_development | 0.0190907717183401 | 0.0140768753051021 | 0.0736964718710755 |
| regulation\_of\_angiogenesis | 0.0190907717183401 | 0.0140768753051021 | 0.0736964718710755 |
| negative\_regulation\_of\_protein\_catabolic\_process | 0.0190907717183401 | 0.13572692472703 | 0.00562209785600918 |
| chemokine-mediated\_signaling\_pathway | 0.0190907717183401 | 0.0140768753051021 | 0.0736964718710755 |
| CenH3-containing\_nucleosome\_assembly\_at\_centromere | 0.0190907717183401 | 0.13572692472703 | 0.00562209785600918 |
| telomere\_maintenance\_via\_semi-conservative\_replication | 0.0177517370442636 | 0.130938880233837 | 0.0052201455943604 |
| response\_to\_morphine | 0.0177517370442636 | 0.130938880233837 | 0.0052201455943604 |
| regulation\_of\_cell\_differentiation | 0.0177517370442636 | 0.0137238830109637 | 0.0711687853011158 |
| positive\_regulation\_of\_transforming\_growth\_factor\_beta\_receptor\_signaling\_pathway | 0.0177517370442636 | 0.0137238830109637 | 0.0711687853011158 |
| positive\_regulation\_of\_release\_of\_cytochrome\_c\_from\_mitochondria | 0.0177517370442636 | 0.130938880233837 | 0.0052201455943604 |
| positive\_regulation\_of\_interferon-beta\_production | 0.0177517370442636 | 0.0137238830109637 | 0.0711687853011158 |
| negative\_regulation\_of\_protein\_kinase\_B\_signaling\_cascade | 0.0177517370442636 | 0.0137238830109637 | 0.0711687853011158 |
| enteric\_nervous\_system\_development | 0.0177517370442636 | 0.130938880233837 | 0.0052201455943604 |
| defense\_response\_to\_Gram-negative\_bacterium | 0.00196317351177434 | 0.0137238830109637 | 0.0052201455943604 |
| cochlea\_morphogenesis | 0.0177517370442636 | 0.130938880233837 | 0.0052201455943604 |
| cellular\_response\_to\_interferon-gamma | 0.0177517370442636 | 0.130938880233837 | 0.0052201455943604 |
| calcium-independent\_cell-cell\_adhesion | 8.14126824332053e-06 | 8.62634673092128e-05 | 0.0052201455943604 |
| bile\_acid\_biosynthetic\_process | 0.0177517370442636 | 0.130938880233837 | 0.0052201455943604 |
| WW\_domain\_binding | 0.00189036127992788 | 0.00115782417201472 | 0.0686770600342676 |
| T\_cell\_homeostasis | 0.0165891624515192 | 0.126882722978549 | 0.00493233606366398 |
| smooth\_muscle\_contraction | 0.0165891624515192 | 0.126882722978549 | 0.00493233606366398 |
| negative\_regulation\_of\_phosphorylation | 0.00189036127992788 | 0.0129048466918583 | 0.00493233606366398 |
| mitotic\_spindle | 0.0165891624515192 | 0.126882722978549 | 0.00493233606366398 |
| L-ascorbic\_acid\_binding | 0.0165891624515192 | 0.0129048466918583 | 0.0686770600342676 |
| extracellular\_matrix\_binding | 0.00189036127992788 | 0.126882722978549 | 0.000263009486641159 |
| cartilage\_condensation | 0.0165891624515192 | 0.126882722978549 | 0.00493233606366398 |
| Wnt-activated\_receptor\_activity | 0.0152291126406607 | 0.0119287954071599 | 0.0658059816180355 |
| very-low-density\_lipoprotein\_particle | 0.0152291126406607 | 0.0119287954071599 | 0.0658059816180355 |
| regulation\_of\_transcription\_involved\_in\_G1/S\_phase\_of\_mitotic\_cell\_cycle | 0.0152291126406607 | 0.122193763703457 | 0.00455101461065887 |
| protein\_N-linked\_glycosylation | 0.0152291126406607 | 0.0119287954071599 | 0.0658059816180355 |
| positive\_regulation\_of\_interleukin-8\_production | 0.0152291126406607 | 0.0119287954071599 | 0.0658059816180355 |
| positive\_regulation\_of\_erythrocyte\_differentiation | 0.0152291126406607 | 0.122193763703457 | 0.00455101461065887 |
| positive\_regulation\_of\_cardiac\_muscle\_cell\_proliferation | 0.0152291126406607 | 0.122193763703457 | 0.00455101461065887 |
| oogenesis | 0.0152291126406607 | 0.122193763703457 | 0.00455101461065887 |
| negative\_regulation\_of\_proteolysis | 0.0152291126406607 | 0.0119287954071599 | 0.0658059816180355 |
| motor\_axon\_guidance | 0.0152291126406607 | 0.0119287954071599 | 0.0658059816180355 |
| hyaluronic\_acid\_binding | 0.000189381121121242 | 0.122193763703457 | 6.98100628934373e-06 |
| hyaluronan\_metabolic\_process | 0.00165808043407912 | 0.122193763703457 | 0.000257057415509242 |
| glycosaminoglycan\_binding | 0.0152291126406607 | 0.122193763703457 | 0.00455101461065887 |
| T\_cell\_proliferation | 0.0140520743302416 | 0.0110782436810008 | 0.0632086651863861 |
| sodium\_channel\_regulator\_activity | 0.0140520743302416 | 0.117896600483996 | 0.00422540377108029 |
| nucleotide-excision\_repair\_\_DNA\_gap\_filling | 0.0140520743302416 | 0.117896600483996 | 0.00422540377108029 |
| negative\_regulation\_of\_osteoclast\_differentiation | 0.0140520743302416 | 0.0110782436810008 | 0.0632086651863861 |
| mitotic\_sister\_chromatid\_segregation | 0.0140520743302416 | 0.117896600483996 | 0.00422540377108029 |
| galactosyltransferase\_activity | 0.0140520743302416 | 0.0110782436810008 | 0.0632086651863861 |
| focal\_adhesion\_assembly | 0.0140520743302416 | 0.117896600483996 | 0.00422540377108029 |
| cellular\_response\_to\_fibroblast\_growth\_factor\_stimulus | 0.00142293806128788 | 0.0110782436810008 | 0.00422540377108029 |
| anchored\_to\_plasma\_membrane | 0.0140520743302416 | 0.0110782436810008 | 0.0632086651863861 |
| retinol\_metabolic\_process | 0.0128957159403053 | 0.0101360658026672 | 0.0604013342804693 |
| response\_to\_X-ray | 0.0128957159403053 | 0.112680541566626 | 0.00385895458243647 |
| response\_to\_cadmium\_ion | 0.0128957159403053 | 0.112680541566626 | 0.00385895458243647 |
| positive\_regulation\_of\_protein\_serine/threonine\_kinase\_activity | 0.0128957159403053 | 0.112680541566626 | 0.00385895458243647 |
| positive\_regulation\_of\_neuroblast\_proliferation | 0.0128957159403053 | 0.112680541566626 | 0.00385895458243647 |
| neurotransmitter:sodium\_symporter\_activity | 0.0128957159403053 | 0.112680541566626 | 0.00385895458243647 |
| carboxypeptidase\_activity | 0.0128957159403053 | 0.0101360658026672 | 0.0604013342804693 |
| anchored\_to\_external\_side\_of\_plasma\_membrane | 0.0128957159403053 | 0.0101360658026672 | 0.0604013342804693 |
| sodium\_channel\_activity | 0.00124798632317943 | 0.000711046315092279 | 0.0574719190186974 |
| positive\_regulation\_of\_smoothened\_signaling\_pathway | 0.0117624259796155 | 0.00915573664022089 | 0.0574719190186974 |
| positive\_regulation\_of\_Rac\_GTPase\_activity | 0.0117624259796155 | 0.00915573664022089 | 0.0574719190186974 |
| positive\_regulation\_of\_interleukin-1\_beta\_secretion | 0.0117624259796155 | 0.107944752099081 | 0.00352999860725928 |
| positive\_regulation\_of\_cell-matrix\_adhesion | 0.0117624259796155 | 0.00915573664022089 | 0.0574719190186974 |
| negative\_regulation\_of\_growth | 0.0117624259796155 | 0.00915573664022089 | 0.0574719190186974 |
| negative\_regulation\_of\_G1/S\_transition\_of\_mitotic\_cell\_cycle | 0.0117624259796155 | 0.00915573664022089 | 0.0574719190186974 |
| long-chain\_fatty-acyl-CoA\_biosynthetic\_process | 0.0117624259796155 | 0.107944752099081 | 0.00352999860725928 |
| long-chain\_fatty\_acid\_metabolic\_process | 0.0117624259796155 | 0.107944752099081 | 0.00352999860725928 |
| intracellular\_transport | 0.0117624259796155 | 0.00915573664022089 | 0.0574719190186974 |
| cortical\_actin\_cytoskeleton\_organization | 0.0117624259796155 | 0.00915573664022089 | 0.0574719190186974 |
| core\_promoter\_proximal\_region\_sequence-specific\_DNA\_binding | 0.0117624259796155 | 0.00915573664022089 | 0.0574719190186974 |
| cation\_transmembrane\_transporter\_activity | 0.0117624259796155 | 0.00915573664022089 | 0.0574719190186974 |
| bile\_acid\_and\_bile\_salt\_transport | 0.0117624259796155 | 0.00915573664022089 | 0.0574719190186974 |
| activation\_of\_protein\_kinase\_B\_activity | 0.0117624259796155 | 0.107944752099081 | 0.00352999860725928 |
| 3\_5-cyclic-nucleotide\_phosphodiesterase\_activity | 0.0117624259796155 | 0.00915573664022089 | 0.0574719190186974 |
| zinc\_ion\_transport | 0.00104751858502909 | 0.00840906265318876 | 0.00316941950425519 |
| vasoconstriction | 0.0108337199536976 | 0.103215085788631 | 0.00316941950425519 |
| response\_to\_exogenous\_dsRNA | 0.0108337199536976 | 0.00840906265318876 | 0.00316941950425519 |
| regulation\_of\_interferon-gamma-mediated\_signaling\_pathway | 0.0108337199536976 | 0.00840906265318876 | 0.00316941950425519 |
| positive\_regulation\_of\_release\_of\_sequestered\_calcium\_ion\_into\_cytosol | 0.0108337199536976 | 0.103215085788631 | 0.00316941950425519 |
| positive\_regulation\_of\_osteoclast\_differentiation | 0.0108337199536976 | 0.00840906265318876 | 0.0550507811129059 |
| positive\_regulation\_of\_neutrophil\_chemotaxis | 0.0108337199536976 | 0.103215085788631 | 0.00316941950425519 |
| positive\_regulation\_of\_defense\_response\_to\_virus\_by\_host | 0.0108337199536976 | 0.00840906265318876 | 0.0550507811129059 |
| positive\_regulation\_of\_chemokine\_production | 0.0108337199536976 | 0.00840906265318876 | 0.0550507811129059 |
| neural\_crest\_cell\_development | 0.0108337199536976 | 0.103215085788631 | 0.00316941950425519 |
| negative\_regulation\_of\_ossification | 0.0108337199536976 | 0.00840906265318876 | 0.0550507811129059 |
| monocyte\_chemotaxis | 0.0108337199536976 | 0.00840906265318876 | 0.0550507811129059 |
| digestive\_tract\_morphogenesis | 0.0108337199536976 | 0.00840906265318876 | 0.0550507811129059 |
| detection\_of\_mechanical\_stimulus\_involved\_in\_sensory\_perception\_of\_sound | 0.0108337199536976 | 0.103215085788631 | 0.00316941950425519 |
| cyclin\_binding | 0.00104751858502909 | 0.00840906265318876 | 0.00316941950425519 |
| cellular\_response\_to\_extracellular\_stimulus | 0.0108337199536976 | 0.00840906265318876 | 0.0550507811129059 |
| cellular\_response\_to\_BMP\_stimulus | 0.0108337199536976 | 0.00840906265318876 | 0.0550507811129059 |
| synaptonemal\_complex\_assembly | 0.0098340431435319 | 0.00769755349131371 | 0.0523551840199572 |
| sulfate\_transport | 0.0098340431435319 | 0.00769755349131371 | 0.0523551840199572 |
| sterol\_metabolic\_process | 0.0098340431435319 | 0.0977779729657771 | 0.00289295028711324 |
| signal\_transduction\_by\_p53\_class\_mediator\_resulting\_in\_induction\_of\_apoptosis | 0.0098340431435319 | 0.0977779729657771 | 0.00289295028711324 |
| selenium\_binding | 0.0098340431435319 | 0.00769755349131371 | 0.0523551840199572 |
| secretory\_granule\_membrane | 0.0098340431435319 | 0.00769755349131371 | 0.0523551840199572 |
| retinol\_dehydrogenase\_activity | 0.0098340431435319 | 0.00769755349131371 | 0.0523551840199572 |
| receptor\_agonist\_activity | 0.0098340431435319 | 0.0977779729657771 | 0.00289295028711324 |
| phospholipid-translocating\_ATPase\_activity | 0.0098340431435319 | 0.0977779729657771 | 0.00289295028711324 |
| neuroblast\_proliferation | 0.0098340431435319 | 0.0977779729657771 | 0.00289295028711324 |
| induction\_of\_positive\_chemotaxis | 0.0098340431435319 | 0.00769755349131371 | 0.0523551840199572 |
| Hsp70\_protein\_binding | 0.0098340431435319 | 0.0977779729657771 | 0.00289295028711324 |
| hemopoietic\_progenitor\_cell\_differentiation | 0.000894897978066401 | 0.00769755349131371 | 0.00289295028711324 |
| female\_gonad\_development | 0.0098340431435319 | 0.00769755349131371 | 0.0523551840199572 |
| chromosome\_condensation | 0.0098340431435319 | 0.0977779729657771 | 0.00289295028711324 |
| ATP\_metabolic\_process | 0.0098340431435319 | 0.00769755349131371 | 0.0523551840199572 |
| androgen\_metabolic\_process | 0.0098340431435319 | 0.00769755349131371 | 0.0523551840199572 |
| suckling\_behavior | 0.00886878064553648 | 0.0928697155646234 | 0.00261996057589775 |
| response\_to\_copper\_ion | 0.00886878064553648 | 0.0928697155646234 | 0.00261996057589775 |
| positive\_regulation\_of\_peptidyl-threonine\_phosphorylation | 0.00886878064553648 | 0.0928697155646234 | 0.00261996057589775 |
| phospholipid\_translocation | 0.00886878064553648 | 0.0928697155646234 | 0.00261996057589775 |
| negative\_regulation\_of\_epithelial\_to\_mesenchymal\_transition | 0.00886878064553648 | 0.00704514540740792 | 0.049934907124938 |
| N-acetylglucosamine\_metabolic\_process | 0.00886878064553648 | 0.00704514540740792 | 0.049934907124938 |
| morphogenesis\_of\_an\_epithelium | 0.00886878064553648 | 0.00704514540740792 | 0.049934907124938 |
| metal\_ion\_transmembrane\_transporter\_activity | 0.00886878064553648 | 0.0928697155646234 | 0.00261996057589775 |
| cellular\_response\_to\_glucose\_starvation | 0.00886878064553648 | 0.0928697155646234 | 0.00261996057589775 |
| carbonate\_dehydratase\_activity | 6.92047476863909e-07 | 1.58892409830466e-07 | 0.049934907124938 |
| acyl-CoA\_metabolic\_process | 0.00886878064553648 | 0.0928697155646234 | 0.00261996057589775 |
| sulfur\_compound\_metabolic\_process | 0.0078074625037485 | 0.00622097804893065 | 0.0470422113290908 |
| regulation\_of\_GTPase\_activity | 0.0078074625037485 | 0.0877788878799382 | 0.00231957636422725 |
| positive\_regulation\_of\_protein\_import\_into\_nucleus | 0.0078074625037485 | 0.00622097804893065 | 0.0470422113290908 |
| positive\_regulation\_of\_focal\_adhesion\_assembly | 0.0078074625037485 | 0.00622097804893065 | 0.0470422113290908 |
| planar\_cell\_polarity\_pathway\_involved\_in\_neural\_tube\_closure | 0.000770688480275593 | 0.00622097804893065 | 0.00231957636422725 |
| negative\_regulation\_of\_androgen\_receptor\_signaling\_pathway | 0.0078074625037485 | 0.00622097804893065 | 0.0470422113290908 |
| low-density\_lipoprotein\_particle | 0.0078074625037485 | 0.00622097804893065 | 0.0470422113290908 |
| inorganic\_anion\_exchanger\_activity | 0.0078074625037485 | 0.00622097804893065 | 0.0470422113290908 |
| hyaluronan\_catabolic\_process | 0.000770688480275593 | 0.0877788878799382 | 0.000118948548338394 |
| elevation\_of\_cytosolic\_calcium\_ion\_concentration\_involved\_in\_phospholipase\_C-activating\_G-protein\_coupled\_signaling\_pathway | 0.0078074625037485 | 0.0877788878799382 | 0.00231957636422725 |
| dopaminergic\_neuron\_differentiation | 0.0078074625037485 | 0.0877788878799382 | 0.00231957636422725 |
| chondroitin\_sulfate\_catabolic\_process | 0.0078074625037485 | 0.0877788878799382 | 0.00231957636422725 |
| response\_to\_steroid\_hormone\_stimulus | 0.000610726829028144 | 0.00544862454179297 | 0.0020107532278998 |
| regulation\_of\_mitochondrial\_membrane\_permeability | 0.00681693473010034 | 0.0823772454864315 | 0.0020107532278998 |
| positive\_regulation\_of\_protein\_complex\_assembly | 0.00681693473010034 | 0.0823772454864315 | 0.0020107532278998 |
| positive\_regulation\_of\_kidney\_development | 0.00681693473010034 | 0.0823772454864315 | 0.0020107532278998 |
| positive\_regulation\_of\_DNA\_damage\_response\_\_signal\_transduction\_by\_p53\_class\_mediator | 0.00681693473010034 | 0.0823772454864315 | 0.0020107532278998 |
| positive\_regulation\_of\_bone\_resorption | 0.00681693473010034 | 0.00544862454179297 | 0.0441221322681718 |
| neural\_tube\_formation | 0.00681693473010034 | 0.0823772454864315 | 0.0020107532278998 |
| negative\_regulation\_of\_multicellular\_organism\_growth | 0.00681693473010034 | 0.00544862454179297 | 0.0441221322681718 |
| negative\_regulation\_of\_JUN\_kinase\_activity | 0.00681693473010034 | 0.00544862454179297 | 0.0441221322681718 |
| negative\_regulation\_of\_DNA\_damage\_response\_\_signal\_transduction\_by\_p53\_class\_mediator | 0.00681693473010034 | 0.0823772454864315 | 0.0020107532278998 |
| mRNA\_cleavage | 0.00681693473010034 | 0.0823772454864315 | 0.0020107532278998 |
| keratan\_sulfate\_catabolic\_process | 0.000610726829028144 | 0.000364743732317198 | 0.0441221322681718 |
| hemopoietic\_stem\_cell\_proliferation | 0.00681693473010034 | 0.0823772454864315 | 0.0020107532278998 |
| cyclin-dependent\_protein\_kinase\_inhibitor\_activity | 0.00681693473010034 | 0.00544862454179297 | 0.0441221322681718 |
| chylomicron | 0.00681693473010034 | 0.00544862454179297 | 0.0441221322681718 |
| cellular\_response\_to\_zinc\_ion | 0.00681693473010034 | 0.00544862454179297 | 0.0441221322681718 |
| axolemma | 0.00681693473010034 | 0.00544862454179297 | 0.0441221322681718 |
| angiotensin\_maturation | 0.00681693473010034 | 0.00544862454179297 | 0.0441221322681718 |
| ventricular\_cardiac\_muscle\_cell\_development | 0.00596918002586721 | 0.0773552946811223 | 0.00179293589730835 |
| vagina\_development | 0.00596918002586721 | 0.0773552946811223 | 0.00179293589730835 |
| Schmidt-Lanterman\_incisure | 0.00596918002586721 | 0.00478412095789432 | 0.0412179321285236 |
| RNA\_polymerase\_II\_transcription\_factor\_binding\_transcription\_factor\_activity\_involved\_in\_positive\_regulation\_of\_transcription | 0.00596918002586721 | 0.00478412095789432 | 0.0412179321285236 |
| regulation\_of\_embryonic\_development | 0.00596918002586721 | 0.0773552946811223 | 0.00179293589730835 |
| positive\_regulation\_of\_ossification | 0.00596918002586721 | 0.0773552946811223 | 0.00179293589730835 |
| positive\_regulation\_of\_interferon-alpha\_production | 0.00596918002586721 | 0.00478412095789432 | 0.0412179321285236 |
| positive\_regulation\_of\_cartilage\_development | 0.00596918002586721 | 0.0773552946811223 | 0.00179293589730835 |
| negative\_regulation\_of\_phosphatidylinositol\_3-kinase\_cascade | 0.00596918002586721 | 0.00478412095789432 | 0.0412179321285236 |
| negative\_regulation\_of\_peptidyl-tyrosine\_phosphorylation | 0.00596918002586721 | 0.00478412095789432 | 0.0412179321285236 |
| membrane\_hyperpolarization | 0.00596918002586721 | 0.00478412095789432 | 0.0412179321285236 |
| ethanol\_oxidation | 0.00596918002586721 | 0.00478412095789432 | 0.0412179321285236 |
| dorsal/ventral\_axis\_specification | 0.00596918002586721 | 0.00478412095789432 | 0.0412179321285236 |
| dermatan\_sulfate\_biosynthetic\_process | 0.00596918002586721 | 0.0773552946811223 | 0.00179293589730835 |
| cytoskeletal\_anchoring\_at\_plasma\_membrane | 0.00596918002586721 | 0.00478412095789432 | 0.0412179321285236 |
| chondrocyte\_development | 0.00596918002586721 | 0.0773552946811223 | 0.00179293589730835 |
| cholesterol\_catabolic\_process | 0.00596918002586721 | 0.0773552946811223 | 0.00179293589730835 |
| cellular\_response\_to\_cholesterol | 0.00596918002586721 | 0.0773552946811223 | 0.00179293589730835 |
| cellular\_aldehyde\_metabolic\_process | 0.00596918002586721 | 0.00478412095789432 | 0.0412179321285236 |
| cell\_recognition | 0.00596918002586721 | 0.0773552946811223 | 0.00179293589730835 |
| androgen\_biosynthetic\_process | 0.00596918002586721 | 0.00478412095789432 | 0.0412179321285236 |
| anagen | 0.00596918002586721 | 0.00478412095789432 | 0.0412179321285236 |
| water\_channel\_activity | 0.00523908898521749 | 0.0041907646433162 | 0.0384678742323426 |
| uterus\_development | 0.00523908898521749 | 0.0721732877551033 | 0.00157700175858376 |
| urate\_metabolic\_process | 0.00523908898521749 | 0.0041907646433162 | 0.0384678742323426 |
| type\_B\_pancreatic\_cell\_development | 0.00523908898521749 | 0.0721732877551033 | 0.00157700175858376 |
| response\_to\_pH | 0.00523908898521749 | 0.0041907646433162 | 0.0384678742323426 |
| positive\_regulation\_of\_protein\_import\_into\_nucleus\_\_translocation | 0.000440511814719415 | 0.0041907646433162 | 0.00157700175858376 |
| positive\_regulation\_of\_monocyte\_chemotaxis | 0.00523908898521749 | 0.0041907646433162 | 0.0384678742323426 |
| positive\_regulation\_of\_macrophage\_activation | 0.00523908898521749 | 0.0721732877551033 | 0.00157700175858376 |
| paranode\_region\_of\_axon | 0.000440511814719415 | 0.000305570458974976 | 0.0384678742323426 |
| organic\_cation\_transport | 0.00523908898521749 | 0.0721732877551033 | 0.00157700175858376 |
| organ\_development | 0.00523908898521749 | 0.0041907646433162 | 0.0384678742323426 |
| negative\_regulation\_of\_transcription\_by\_competitive\_promoter\_binding | 0.00523908898521749 | 0.0721732877551033 | 0.00157700175858376 |
| negative\_regulation\_of\_organ\_growth | 0.00523908898521749 | 0.0721732877551033 | 0.00157700175858376 |
| negative\_regulation\_of\_fibroblast\_growth\_factor\_receptor\_signaling\_pathway | 0.00523908898521749 | 0.0721732877551033 | 0.00157700175858376 |
| negative\_regulation\_of\_cAMP\_biosynthetic\_process | 0.00523908898521749 | 0.0721732877551033 | 0.00157700175858376 |
| long-chain\_fatty\_acid-CoA\_ligase\_activity | 0.00523908898521749 | 0.0721732877551033 | 0.00157700175858376 |
| ion\_transmembrane\_transporter\_activity | 0.00523908898521749 | 0.0721732877551033 | 0.00157700175858376 |
| head\_development | 0.00523908898521749 | 0.0721732877551033 | 0.00157700175858376 |
| germ\_cell\_migration | 0.00523908898521749 | 0.0041907646433162 | 0.0384678742323426 |
| eyelid\_development\_in\_camera-type\_eye | 0.00523908898521749 | 0.0721732877551033 | 0.00157700175858376 |
| establishment\_of\_planar\_polarity | 0.00523908898521749 | 0.0721732877551033 | 0.00157700175858376 |
| drug\_transmembrane\_transport | 0.000440511814719415 | 0.0041907646433162 | 0.00157700175858376 |
| creatine\_metabolic\_process | 0.00523908898521749 | 0.0041907646433162 | 0.0384678742323426 |
| body\_fluid\_secretion | 0.00523908898521749 | 0.0041907646433162 | 0.0384678742323426 |
| activin\_receptor\_signaling\_pathway | 0.00523908898521749 | 0.0721732877551033 | 0.00157700175858376 |
| activation\_of\_innate\_immune\_response | 0.00523908898521749 | 0.0041907646433162 | 0.0384678742323426 |
| wound\_healing\_\_spreading\_of\_epidermal\_cells | 0.000437057278156203 | 0.0670363708078528 | 4.84803433656845e-05 |
| ubiquitin\_conjugating\_enzyme\_binding | 0.000437057278156203 | 0.0670363708078528 | 4.84803433656845e-05 |
| secondary\_active\_sulfate\_transmembrane\_transporter\_activity | 0.00450563597812728 | 0.00362339220323195 | 0.0354626691182368 |
| proteoglycan\_metabolic\_process | 0.00450563597812728 | 0.0670363708078528 | 0.00137916014007048 |
| protein\_serine/threonine\_kinase\_activator\_activity | 0.00450563597812728 | 0.0670363708078528 | 0.00137916014007048 |
| primitive\_streak\_formation | 0.00450563597812728 | 0.0670363708078528 | 0.00137916014007048 |
| positive\_regulation\_of\_triglyceride\_biosynthetic\_process | 0.00450563597812728 | 0.0670363708078528 | 0.00137916014007048 |
| positive\_regulation\_of\_transcription\_factor\_import\_into\_nucleus | 0.00450563597812728 | 0.00362339220323195 | 0.0354626691182368 |
| positive\_regulation\_of\_protein\_oligomerization | 0.00450563597812728 | 0.0670363708078528 | 0.00137916014007048 |
| positive\_regulation\_of\_leukocyte\_chemotaxis | 0.00450563597812728 | 0.00362339220323195 | 0.0354626691182368 |
| positive\_regulation\_of\_chemokine\_biosynthetic\_process | 0.00450563597812728 | 0.0670363708078528 | 0.00137916014007048 |
| negative\_regulation\_of\_immune\_response | 0.00450563597812728 | 0.00362339220323195 | 0.0354626691182368 |
| negative\_regulation\_of\_axon\_extension\_involved\_in\_axon\_guidance | 0.00450563597812728 | 0.0670363708078528 | 0.00137916014007048 |
| negative\_chemotaxis | 0.00450563597812728 | 0.0670363708078528 | 0.00137916014007048 |
| midgut\_development | 0.00450563597812728 | 0.0670363708078528 | 0.00137916014007048 |
| ligand-gated\_sodium\_channel\_activity | 0.000437057278156203 | 0.000220895856766127 | 0.0354626691182368 |
| hemopoietic\_stem\_cell\_differentiation | 0.00450563597812728 | 0.00362339220323195 | 0.0354626691182368 |
| glial\_cell\_migration | 0.00450563597812728 | 0.0670363708078528 | 0.00137916014007048 |
| fatty\_acid\_transport | 0.00450563597812728 | 0.0670363708078528 | 0.00137916014007048 |
| face\_development | 0.00450563597812728 | 0.0670363708078528 | 0.00137916014007048 |
| estradiol\_17-beta-dehydrogenase\_activity | 0.00450563597812728 | 0.00362339220323195 | 0.0354626691182368 |
| DNA\_synthesis\_involved\_in\_DNA\_repair | 0.00450563597812728 | 0.0670363708078528 | 0.00137916014007048 |
| cGMP\_biosynthetic\_process | 0.00450563597812728 | 0.00362339220323195 | 0.0354626691182368 |
| cellular\_response\_to\_estrogen\_stimulus | 0.00450563597812728 | 0.00362339220323195 | 0.0354626691182368 |
| cellular\_response\_to\_estradiol\_stimulus | 0.00450563597812728 | 0.00362339220323195 | 0.0354626691182368 |
| anterior/posterior\_axis\_specification\_\_embryo | 0.00450563597812728 | 0.0670363708078528 | 0.00137916014007048 |
| anion\_transport | 0.00450563597812728 | 0.00362339220323195 | 0.0354626691182368 |
| aldo-keto\_reductase\_\_NADP\_\_activity | 0.00450563597812728 | 0.00362339220323195 | 0.0354626691182368 |
| 3\_5-cyclic-GMP\_phosphodiesterase\_activity | 0.00450563597812728 | 0.00362339220323195 | 0.0354626691182368 |
| sperm\_capacitation | 0.00386931702879394 | 0.00301666809172169 | 0.0324013957871399 |
| RNA\_polymerase\_II\_core\_promoter\_sequence-specific\_DNA\_binding\_transcription\_factor\_activity | 0.00386931702879394 | 0.00301666809172169 | 0.00117512921584362 |
| RNA\_polymerase\_II\_carboxy-terminal\_domain\_kinase\_activity | 0.00386931702879394 | 0.0621408600823473 | 0.00117512921584362 |
| resolution\_of\_meiotic\_recombination\_intermediates | 0.00386931702879394 | 0.00301666809172169 | 0.0324013957871399 |
| regulation\_of\_exit\_from\_mitosis | 0.00386931702879394 | 0.0621408600823473 | 0.00117512921584362 |
| regulation\_of\_epithelial\_cell\_proliferation | 0.00386931702879394 | 0.0621408600823473 | 0.00117512921584362 |
| protein\_autoprocessing | 0.00386931702879394 | 0.00301666809172169 | 0.0324013957871399 |
| positive\_regulation\_of\_type\_I\_interferon-mediated\_signaling\_pathway | 0.000317726813356996 | 0.00301666809172169 | 0.00117512921584362 |
| positive\_regulation\_of\_T\_cell\_chemotaxis | 0.00386931702879394 | 0.0621408600823473 | 0.00117512921584362 |
| positive\_regulation\_of\_protein\_kinase\_C\_signaling\_cascade | 0.00386931702879394 | 0.0621408600823473 | 0.00117512921584362 |
| negative\_regulation\_of\_osteoblast\_proliferation | 0.00386931702879394 | 0.00301666809172169 | 0.0324013957871399 |
| negative\_regulation\_of\_macrophage\_differentiation | 0.00386931702879394 | 0.0621408600823473 | 0.00117512921584362 |
| negative\_regulation\_of\_kinase\_activity | 0.00386931702879394 | 0.0621408600823473 | 0.00117512921584362 |
| megakaryocyte\_differentiation | 0.00386931702879394 | 0.00301666809172169 | 0.0324013957871399 |
| juxtaparanode\_region\_of\_axon | 0.00386931702879394 | 0.00301666809172169 | 0.0324013957871399 |
| interleukin-6-mediated\_signaling\_pathway | 0.00386931702879394 | 0.00301666809172169 | 0.0324013957871399 |
| heme\_transporter\_activity | 0.00386931702879394 | 0.00301666809172169 | 0.0324013957871399 |
| glucocorticoid\_biosynthetic\_process | 0.00386931702879394 | 0.00301666809172169 | 0.0324013957871399 |
| doxorubicin\_metabolic\_process | 0.00386931702879394 | 0.00301666809172169 | 0.0324013957871399 |
| daunorubicin\_metabolic\_process | 0.00386931702879394 | 0.00301666809172169 | 0.0324013957871399 |
| cellular\_response\_to\_interferon-beta | 0.00386931702879394 | 0.00301666809172169 | 0.00117512921584362 |
| cellular\_response\_to\_follicle-stimulating\_hormone\_stimulus | 0.00386931702879394 | 0.0621408600823473 | 0.00117512921584362 |
| CARD\_domain\_binding | 0.00386931702879394 | 0.00301666809172169 | 0.0324013957871399 |
| cAMP-dependent\_protein\_kinase\_inhibitor\_activity | 0.00386931702879394 | 0.00301666809172169 | 0.0324013957871399 |
| bone\_trabecula\_formation | 0.00386931702879394 | 0.00301666809172169 | 0.0324013957871399 |
| anion:anion\_antiporter\_activity | 0.00386931702879394 | 0.00301666809172169 | 0.0324013957871399 |
| alpha-catenin\_binding | 0.00386931702879394 | 0.0621408600823473 | 0.00117512921584362 |
| alcohol\_dehydrogenase\_\_NAD\_\_activity | 0.00386931702879394 | 0.00301666809172169 | 0.0324013957871399 |
| zymogen\_granule\_membrane | 0.00318729002303362 | 0.00254603755891334 | 0.0294760801726734 |
| Wnt\_receptor\_signaling\_pathway\_involved\_in\_somitogenesis | 0.00318729002303362 | 0.00254603755891334 | 0.0294760801726734 |
| semaphorin\_receptor\_binding | 0.00318729002303362 | 0.00254603755891334 | 0.0294760801726734 |
| retinal\_dehydrogenase\_activity | 0.00318729002303362 | 0.00254603755891334 | 0.0294760801726734 |
| response\_to\_gravity | 0.00318729002303362 | 0.0562256293167479 | 0.000960830945347445 |
| response\_to\_amine\_stimulus | 0.00318729002303362 | 0.0562256293167479 | 0.000960830945347445 |
| regulation\_of\_T\_cell\_differentiation | 0.00318729002303362 | 0.00254603755891334 | 0.0294760801726734 |
| regulation\_of\_intracellular\_pH | 0.00318729002303362 | 0.00254603755891334 | 0.0294760801726734 |
| regulation\_of\_branching\_involved\_in\_prostate\_gland\_morphogenesis | 0.00318729002303362 | 0.00254603755891334 | 0.0294760801726734 |
| protein\_localization\_to\_kinetochore | 0.00318729002303362 | 0.0562256293167479 | 0.000960830945347445 |
| prostate\_epithelial\_cord\_arborization\_involved\_in\_prostate\_glandular\_acinus\_morphogenesis | 0.00318729002303362 | 0.00254603755891334 | 0.0294760801726734 |
| progesterone\_metabolic\_process | 0.00318729002303362 | 0.00254603755891334 | 0.0294760801726734 |
| positive\_regulation\_of\_epithelial\_cell\_differentiation | 0.00318729002303362 | 0.00254603755891334 | 0.0294760801726734 |
| organic\_cation\_transmembrane\_transporter\_activity | 0.00318729002303362 | 0.0562256293167479 | 0.000960830945347445 |
| negative\_regulation\_of\_skeletal\_muscle\_tissue\_development | 0.00318729002303362 | 0.00254603755891334 | 0.0294760801726734 |
| negative\_regulation\_of\_cell\_migration\_involved\_in\_sprouting\_angiogenesis | 0.00318729002303362 | 0.00254603755891334 | 0.0294760801726734 |
| mitotic\_cell\_cycle\_G2/M\_transition\_DNA\_damage\_checkpoint | 0.00318729002303362 | 0.0562256293167479 | 0.000960830945347445 |
| hyalurononglucosaminidase\_activity | 0.00318729002303362 | 0.0562256293167479 | 0.000960830945347445 |
| glomerular\_filtration | 0.00318729002303362 | 0.0562256293167479 | 0.000960830945347445 |
| genitalia\_development | 0.00318729002303362 | 0.0562256293167479 | 0.000960830945347445 |
| epidermis\_morphogenesis | 0.00318729002303362 | 0.00254603755891334 | 0.0294760801726734 |
| collagen\_metabolic\_process | 0.00318729002303362 | 0.00254603755891334 | 0.0294760801726734 |
| chloride\_transmembrane\_transporter\_activity | 0.00318729002303362 | 0.00254603755891334 | 0.0294760801726734 |
| cellular\_response\_to\_prostaglandin\_E\_stimulus | 0.00318729002303362 | 0.00254603755891334 | 0.0294760801726734 |
| ameboidal\_cell\_migration | 0.000209771679698566 | 0.00254603755891334 | 0.000960830945347445 |
| 3-beta-hydroxy-delta5-steroid\_dehydrogenase\_activity | 0.00318729002303362 | 0.00254603755891334 | 0.0294760801726734 |
| Wnt\_receptor\_signaling\_pathway\_\_calcium\_modulating\_pathway | 0.00257376048896922 | 0.0497569970718571 | 0.000732242206204171 |
| spongiotrophoblast\_layer\_development | 0.00257376048896922 | 0.0497569970718571 | 0.000732242206204171 |
| sperm\_midpiece | 0.00257376048896922 | 0.00205031857247974 | 0.0266039259874058 |
| smooth\_endoplasmic\_reticulum\_membrane | 0.00257376048896922 | 0.00205031857247974 | 0.0266039259874058 |
| secretion | 0.00257376048896922 | 0.00205031857247974 | 0.0266039259874058 |
| regulation\_of\_cell\_cycle\_process | 0.00257376048896922 | 0.00205031857247974 | 0.0266039259874058 |
| regulation\_of\_branching\_involved\_in\_mammary\_gland\_duct\_morphogenesis | 0.00257376048896922 | 0.0497569970718571 | 0.000732242206204171 |
| regulation\_of\_actin\_polymerization\_or\_depolymerization | 0.00257376048896922 | 0.00205031857247974 | 0.0266039259874058 |
| quaternary\_ammonium\_group\_transmembrane\_transporter\_activity | 0.00257376048896922 | 0.0497569970718571 | 0.000732242206204171 |
| positive\_regulation\_of\_thymocyte\_apoptotic\_process | 0.00257376048896922 | 0.0497569970718571 | 0.000732242206204171 |
| positive\_regulation\_of\_skeletal\_muscle\_tissue\_development | 0.00257376048896922 | 0.00205031857247974 | 0.0266039259874058 |
| positive\_regulation\_of\_protein\_metabolic\_process | 0.00257376048896922 | 0.00205031857247974 | 0.0266039259874058 |
| positive\_regulation\_of\_macrophage\_cytokine\_production | 0.00257376048896922 | 0.0497569970718571 | 0.000732242206204171 |
| positive\_regulation\_of\_interleukin-8\_secretion | 0.00257376048896922 | 0.0497569970718571 | 0.000732242206204171 |
| positive\_regulation\_of\_glucose\_metabolic\_process | 0.00257376048896922 | 0.0497569970718571 | 0.000732242206204171 |
| positive\_regulation\_of\_cell-cell\_adhesion\_mediated\_by\_cadherin | 0.00257376048896922 | 0.0497569970718571 | 0.000732242206204171 |
| paranodal\_junction\_assembly | 0.00257376048896922 | 0.00205031857247974 | 0.0266039259874058 |
| neutrophil\_mediated\_immunity | 0.00257376048896922 | 0.00205031857247974 | 0.0266039259874058 |
| negative\_regulation\_of\_interleukin-8\_production | 0.00257376048896922 | 0.00205031857247974 | 0.0266039259874058 |
| negative\_regulation\_of\_heterotypic\_cell-cell\_adhesion | 0.00257376048896922 | 0.00205031857247974 | 0.0266039259874058 |
| maternal\_process\_involved\_in\_parturition | 0.00257376048896922 | 0.0497569970718571 | 0.000732242206204171 |
| heme\_transport | 0.00257376048896922 | 0.00205031857247974 | 0.0266039259874058 |
| hair\_cell\_differentiation | 0.00257376048896922 | 0.00205031857247974 | 0.0266039259874058 |
| gene\_silencing\_by\_miRNA | 0.00257376048896922 | 0.0497569970718571 | 0.000732242206204171 |
| epidermal\_cell\_differentiation | 0.000127169076978153 | 6.01817115571882e-05 | 0.0266039259874058 |
| DNA\_replication\_factor\_C\_complex | 0.00257376048896922 | 0.0497569970718571 | 0.000732242206204171 |
| centrosome\_cycle | 0.00257376048896922 | 0.0497569970718571 | 0.000732242206204171 |
| central\_element | 0.00257376048896922 | 0.00205031857247974 | 0.0266039259874058 |
| cellular\_response\_to\_exogenous\_dsRNA | 0.00257376048896922 | 0.00205031857247974 | 0.0266039259874058 |
| cellular\_chloride\_ion\_homeostasis | 0.00257376048896922 | 0.00205031857247974 | 0.0266039259874058 |
| branching\_involved\_in\_prostate\_gland\_morphogenesis | 0.00257376048896922 | 0.0497569970718571 | 0.000732242206204171 |
| bicarbonate\_transmembrane\_transporter\_activity | 0.00257376048896922 | 0.00205031857247974 | 0.0266039259874058 |
| artery\_smooth\_muscle\_contraction | 0.00257376048896922 | 0.0497569970718571 | 0.000732242206204171 |
| urinary\_bladder\_development | 0.00196317351177434 | 0.04402309151888 | 0.000567013375212593 |
| toxin\_transporter\_activity | 0.00196317351177434 | 0.04402309151888 | 0.000567013375212593 |
| testosterone\_dehydrogenase\_\_NAD\_\_\_activity | 6.51120985497806e-05 | 3.35414670835235e-05 | 0.023608304798247 |
| regulation\_of\_anion\_transport | 0.00196317351177434 | 0.00157694971059026 | 0.023608304798247 |
| protein\_sulfation | 0.00196317351177434 | 0.00157694971059026 | 0.023608304798247 |
| positive\_regulation\_of\_odontogenesis | 0.00196317351177434 | 0.04402309151888 | 0.000567013375212593 |
| positive\_regulation\_of\_fibroblast\_apoptotic\_process | 0.00196317351177434 | 0.00157694971059026 | 0.023608304798247 |
| positive\_regulation\_of\_epithelial\_cell\_proliferation\_involved\_in\_wound\_healing | 0.00196317351177434 | 0.04402309151888 | 0.000567013375212593 |
| positive\_regulation\_of\_calcineurin-NFAT\_signaling\_cascade | 0.00196317351177434 | 0.00157694971059026 | 0.023608304798247 |
| polysaccharide\_digestion | 0.00196317351177434 | 0.00157694971059026 | 0.023608304798247 |
| phosphatidylinositol\_3-kinase\_regulator\_activity | 0.00196317351177434 | 0.00157694971059026 | 0.023608304798247 |
| neural\_crest\_cell\_fate\_commitment | 0.00196317351177434 | 0.00157694971059026 | 0.023608304798247 |
| negative\_regulation\_of\_synapse\_assembly | 0.00196317351177434 | 0.04402309151888 | 0.000567013375212593 |
| negative\_regulation\_of\_response\_to\_cytokine\_stimulus | 0.00196317351177434 | 0.00157694971059026 | 0.023608304798247 |
| negative\_regulation\_of\_leukocyte\_apoptotic\_process | 0.00196317351177434 | 0.00157694971059026 | 0.023608304798247 |
| negative\_regulation\_of\_interferon-gamma\_biosynthetic\_process | 0.00196317351177434 | 0.04402309151888 | 0.000567013375212593 |
| negative\_regulation\_of\_follicle-stimulating\_hormone\_secretion | 0.00196317351177434 | 0.04402309151888 | 0.000567013375212593 |
| negative\_regulation\_of\_B\_cell\_differentiation | 6.51120985497806e-05 | 0.00157694971059026 | 0.000567013375212593 |
| N-acetylglucosamine\_6-O-sulfotransferase\_activity | 0.00196317351177434 | 0.00157694971059026 | 0.023608304798247 |
| mineralocorticoid\_biosynthetic\_process | 0.00196317351177434 | 0.00157694971059026 | 0.023608304798247 |
| metanephric\_mesenchymal\_cell\_differentiation | 0.00196317351177434 | 0.00157694971059026 | 0.000567013375212593 |
| mammary\_gland\_branching\_involved\_in\_thelarche | 0.00196317351177434 | 0.04402309151888 | 0.000567013375212593 |
| IgE\_binding | 0.00196317351177434 | 0.00157694971059026 | 0.023608304798247 |
| hemoglobin\_biosynthetic\_process | 0.00196317351177434 | 0.04402309151888 | 0.000567013375212593 |
| dopamine\_transport | 0.00196317351177434 | 0.04402309151888 | 0.000567013375212593 |
| ciliary\_neurotrophic\_factor-mediated\_signaling\_pathway | 0.00196317351177434 | 0.00157694971059026 | 0.023608304798247 |
| chiasma\_assembly | 0.00196317351177434 | 0.00157694971059026 | 0.023608304798247 |
| cellular\_sodium\_ion\_homeostasis | 0.00196317351177434 | 0.04402309151888 | 0.000567013375212593 |
| cellular\_response\_to\_heparin | 0.00196317351177434 | 0.00157694971059026 | 0.023608304798247 |
| cell\_differentiation\_involved\_in\_embryonic\_placenta\_development | 0.00196317351177434 | 0.04402309151888 | 0.000567013375212593 |
| carbon\_dioxide\_transport | 0.00196317351177434 | 0.00157694971059026 | 0.023608304798247 |
| calcium\_sensitive\_guanylate\_cyclase\_activator\_activity | 0.00196317351177434 | 0.00157694971059026 | 0.023608304798247 |
| beta-galactosidase\_activity | 0.00196317351177434 | 0.00157694971059026 | 0.023608304798247 |
| actomyosin\_contractile\_ring | 0.00196317351177434 | 0.04402309151888 | 0.000567013375212593 |
| 9-cis-retinoic\_acid\_biosynthetic\_process | 0.00196317351177434 | 0.00157694971059026 | 0.023608304798247 |
| virus-infected\_cell\_apoptotic\_process | 0.00140576853311462 | 0.0369215183775715 | 0.000393687917878258 |
| uridine-diphosphatase\_activity | 0.00140576853311462 | 0.00115782417201472 | 0.0203948830732625 |
| spongiotrophoblast\_differentiation | 0.00140576853311462 | 0.0369215183775715 | 0.000393687917878258 |
| sodium\_channel\_complex | 2.89034215802998e-05 | 1.45363734728352e-05 | 0.0203948830732625 |
| response\_to\_organophosphorus | 0.00140576853311462 | 0.0369215183775715 | 0.000393687917878258 |
| regulation\_of\_appetite | 0.00140576853311462 | 0.0369215183775715 | 0.000393687917878258 |
| protein\_localization\_to\_paranode\_region\_of\_axon | 0.00140576853311462 | 0.00115782417201472 | 0.0203948830732625 |
| post-embryonic\_camera-type\_eye\_development | 0.00140576853311462 | 0.00115782417201472 | 0.0203948830732625 |
| positive\_regulation\_of\_mitotic\_cell\_cycle\_spindle\_assembly\_checkpoint | 0.00140576853311462 | 0.0369215183775715 | 0.000393687917878258 |
| positive\_regulation\_of\_meiosis | 0.00140576853311462 | 0.0369215183775715 | 0.000393687917878258 |
| positive\_regulation\_of\_heterotypic\_cell-cell\_adhesion | 0.00140576853311462 | 0.0369215183775715 | 0.000393687917878258 |
| positive\_regulation\_of\_gene\_silencing\_by\_miRNA | 0.00140576853311462 | 0.0369215183775715 | 0.000393687917878258 |
| positive\_regulation\_of\_follicle-stimulating\_hormone\_secretion | 0.00140576853311462 | 0.0369215183775715 | 0.000393687917878258 |
| positive\_regulation\_of\_extrinsic\_apoptotic\_signaling\_pathway\_in\_absence\_of\_ligand | 0.00140576853311462 | 0.0369215183775715 | 0.000393687917878258 |
| penile\_erection | 0.00140576853311462 | 0.0369215183775715 | 0.000393687917878258 |
| olfactory\_bulb\_interneuron\_development | 0.00140576853311462 | 0.0369215183775715 | 0.000393687917878258 |
| non-canonical\_Wnt\_receptor\_signaling\_pathway\_via\_JNK\_cascade | 0.00140576853311462 | 0.0369215183775715 | 0.000393687917878258 |
| negative\_regulation\_of\_Schwann\_cell\_proliferation | 0.00140576853311462 | 0.0369215183775715 | 0.000393687917878258 |
| negative\_regulation\_of\_prostatic\_bud\_formation | 0.00140576853311462 | 0.0369215183775715 | 0.000393687917878258 |
| negative\_regulation\_of\_interleukin-8\_biosynthetic\_process | 0.00140576853311462 | 0.00115782417201472 | 0.0203948830732625 |
| negative\_regulation\_of\_fibroblast\_apoptotic\_process | 0.00140576853311462 | 0.00115782417201472 | 0.0203948830732625 |
| negative\_regulation\_of\_cysteine-type\_endopeptidase\_activity | 0.00140576853311462 | 0.00115782417201472 | 0.0203948830732625 |
| Ndc80\_complex | 0.00140576853311462 | 0.0369215183775715 | 0.000393687917878258 |
| mesodermal\_cell\_fate\_determination | 0.00140576853311462 | 0.00115782417201472 | 0.0203948830732625 |
| menstrual\_cycle\_phase | 0.00140576853311462 | 0.00115782417201472 | 0.0203948830732625 |
| meiotic\_gene\_conversion | 0.00140576853311462 | 0.00115782417201472 | 0.0203948830732625 |
| intracellular\_pH\_elevation | 0.00140576853311462 | 0.00115782417201472 | 0.0203948830732625 |
| histamine\_secretion | 0.00140576853311462 | 0.0369215183775715 | 0.000393687917878258 |
| hindgut\_morphogenesis | 0.00140576853311462 | 0.0369215183775715 | 0.000393687917878258 |
| glycoside\_catabolic\_process | 0.00140576853311462 | 0.00115782417201472 | 0.0203948830732625 |
| fibroblast\_proliferation | 0.00140576853311462 | 0.0369215183775715 | 0.000393687917878258 |
| extracellular\_matrix\_constituent\_\_lubricant\_activity | 0.00140576853311462 | 0.00115782417201472 | 0.0203948830732625 |
| ErbB-2\_class\_receptor\_binding | 0.00140576853311462 | 0.00115782417201472 | 0.0203948830732625 |
| epithelial\_cell\_proliferation\_involved\_in\_mammary\_gland\_duct\_elongation | 0.00140576853311462 | 0.0369215183775715 | 0.000393687917878258 |
| development\_of\_primary\_female\_sexual\_characteristics | 0.00140576853311462 | 0.0369215183775715 | 0.000393687917878258 |
| creatine\_kinase\_activity | 0.00140576853311462 | 0.00115782417201472 | 0.0203948830732625 |
| cellular\_response\_to\_vitamin\_D | 0.00140576853311462 | 0.00115782417201472 | 0.0203948830732625 |
| canalicular\_bile\_acid\_transport | 0.00140576853311462 | 0.00115782417201472 | 0.0203948830732625 |
| alpha-glucosidase\_activity | 0.00140576853311462 | 0.00115782417201472 | 0.0203948830732625 |
| alcohol\_dehydrogenase\_activity\_\_zinc-dependent | 0.00140576853311462 | 0.00115782417201472 | 0.0203948830732625 |
| xenobiotic-transporting\_ATPase\_activity | 0.000894897978066401 | 0.000711046315092279 | 0.0166979583284687 |
| testosterone\_17-beta-dehydrogenase\_\_NAD\_\_\_activity | 0.000894897978066401 | 0.000711046315092279 | 0.0166979583284687 |
| tendon\_development | 0.000894897978066401 | 0.0294946940892483 | 0.000257057415509242 |
| steroid\_delta-isomerase\_activity | 0.000894897978066401 | 0.000711046315092279 | 0.0166979583284687 |
| sodium:bicarbonate\_symporter\_activity | 0.000894897978066401 | 0.000711046315092279 | 0.0166979583284687 |
| smooth\_muscle\_cell\_proliferation | 0.000894897978066401 | 0.0294946940892483 | 0.000257057415509242 |
| RIG-I\_signaling\_pathway | 0.000894897978066401 | 0.000711046315092279 | 0.0166979583284687 |
| retinoic\_acid\_biosynthetic\_process | 0.000894897978066401 | 0.000711046315092279 | 0.0166979583284687 |
| regulation\_of\_phosphatidylinositol\_3-kinase\_cascade | 0.000894897978066401 | 0.000711046315092279 | 0.0166979583284687 |
| regulation\_of\_peroxisome\_organization | 0.000894897978066401 | 0.000711046315092279 | 0.0166979583284687 |
| quaternary\_ammonium\_group\_transport | 0.000894897978066401 | 0.0294946940892483 | 0.000257057415509242 |
| protein\_localization\_to\_juxtaparanode\_region\_of\_axon | 0.000894897978066401 | 0.000711046315092279 | 0.0166979583284687 |
| protein-hormone\_receptor\_activity | 0.000894897978066401 | 0.0294946940892483 | 0.000257057415509242 |
| proteasomal\_protein\_catabolic\_process | 0.000894897978066401 | 0.0294946940892483 | 0.000257057415509242 |
| procollagen-proline\_3-dioxygenase\_activity | 0.000894897978066401 | 0.000711046315092279 | 0.0166979583284687 |
| primitive\_hemopoiesis | 0.000894897978066401 | 0.0294946940892483 | 0.000257057415509242 |
| post-embryonic\_hemopoiesis | 0.000894897978066401 | 0.000711046315092279 | 0.0166979583284687 |
| positive\_regulation\_of\_ovulation | 0.000894897978066401 | 0.0294946940892483 | 0.000257057415509242 |
| positive\_regulation\_of\_chemokine\_\_C-C\_motif\_\_ligand\_5\_production | 0.000894897978066401 | 0.000711046315092279 | 0.0166979583284687 |
| positive\_regulation\_of\_cellular\_pH\_reduction | 0.000894897978066401 | 0.000711046315092279 | 0.0166979583284687 |
| negative\_regulation\_of\_Wnt\_receptor\_signaling\_pathway\_involved\_in\_dorsal/ventral\_axis\_specification | 0.000894897978066401 | 0.000711046315092279 | 0.0166979583284687 |
| negative\_regulation\_of\_planar\_cell\_polarity\_pathway\_involved\_in\_axis\_elongation | 0.000894897978066401 | 0.000711046315092279 | 0.0166979583284687 |
| negative\_regulation\_of\_mitotic\_anaphase-promoting\_complex\_activity | 0.000894897978066401 | 0.0294946940892483 | 0.000257057415509242 |
| negative\_regulation\_of\_mesenchymal\_to\_epithelial\_transition\_involved\_in\_metanephros\_morphogenesis | 0.000894897978066401 | 0.000711046315092279 | 0.000257057415509242 |
| negative\_regulation\_of\_mesenchymal\_cell\_proliferation | 0.000894897978066401 | 0.0294946940892483 | 0.000257057415509242 |
| negative\_regulation\_of\_hippo\_signaling\_cascade | 0.000894897978066401 | 0.0294946940892483 | 0.000257057415509242 |
| negative\_regulation\_of\_collagen\_biosynthetic\_process | 0.000894897978066401 | 0.000711046315092279 | 0.0166979583284687 |
| negative\_regulation\_of\_cell\_proliferation\_involved\_in\_contact\_inhibition | 0.000894897978066401 | 0.0294946940892483 | 0.000257057415509242 |
| N-acetyllactosaminide\_beta-1\_6-N-acetylglucosaminyltransferase\_activity | 0.000894897978066401 | 0.000711046315092279 | 0.0166979583284687 |
| monocyte\_aggregation | 0.000894897978066401 | 0.0294946940892483 | 0.000257057415509242 |
| metanephric\_mesenchymal\_cell\_proliferation\_involved\_in\_metanephros\_development | 0.000894897978066401 | 0.000711046315092279 | 0.000257057415509242 |
| mesodermal\_cell\_differentiation | 0.000894897978066401 | 0.0294946940892483 | 0.000257057415509242 |
| mesenchymal-epithelial\_cell\_signaling | 0.000894897978066401 | 0.0294946940892483 | 0.000257057415509242 |
| intrinsic\_to\_Golgi\_membrane | 0.000894897978066401 | 0.000711046315092279 | 0.0166979583284687 |
| interleukin-6\_receptor\_complex | 0.000894897978066401 | 0.000711046315092279 | 0.0166979583284687 |
| indanol\_dehydrogenase\_activity | 0.000894897978066401 | 0.000711046315092279 | 0.0166979583284687 |
| glycosylceramide\_catabolic\_process | 0.000894897978066401 | 0.000711046315092279 | 0.0166979583284687 |
| epidermal\_lamellar\_body | 0.000894897978066401 | 0.000711046315092279 | 0.0166979583284687 |
| endocardium\_development | 0.000894897978066401 | 0.0294946940892483 | 0.000257057415509242 |
| dopamine\_transmembrane\_transporter\_activity | 0.000894897978066401 | 0.0294946940892483 | 0.000257057415509242 |
| cytoplasmic\_membrane-bounded\_vesicle\_lumen | 0.000894897978066401 | 0.000711046315092279 | 0.0166979583284687 |
| cellular\_response\_to\_X-ray | 0.000894897978066401 | 0.000711046315092279 | 0.0166979583284687 |
| cellular\_response\_to\_nutrient | 0.000894897978066401 | 0.000711046315092279 | 0.0166979583284687 |
| bile\_acid\_catabolic\_process | 0.000894897978066401 | 0.0294946940892483 | 0.000257057415509242 |
| beta-glucosidase\_activity | 0.000894897978066401 | 0.000711046315092279 | 0.0166979583284687 |
| wound\_healing\_involved\_in\_inflammatory\_response | 0.000440511814719415 | 0.022085088918651 | 0.000118948548338394 |
| type\_II\_activin\_receptor\_binding | 0.000440511814719415 | 0.022085088918651 | 0.000118948548338394 |
| taurine\_binding | 0.000440511814719415 | 0.022085088918651 | 0.000118948548338394 |
| response\_to\_dsRNA | 0.000440511814719415 | 0.022085088918651 | 0.000118948548338394 |
| renal\_tubule\_development | 0.000440511814719415 | 0.000364743732317198 | 0.000118948548338394 |
| regulation\_of\_cellular\_response\_to\_hypoxia | 0.000440511814719415 | 0.022085088918651 | 0.000118948548338394 |
| progesterone\_secretion | 0.000440511814719415 | 0.022085088918651 | 0.000118948548338394 |
| positive\_regulation\_of\_response\_to\_cytokine\_stimulus | 0.000440511814719415 | 0.022085088918651 | 0.000118948548338394 |
| positive\_regulation\_of\_phosphatase\_activity | 0.000440511814719415 | 0.000364743732317198 | 0.0125655770024194 |
| positive\_regulation\_of\_non-canonical\_Wnt\_receptor\_signaling\_pathway | 0.000440511814719415 | 0.000364743732317198 | 0.0125655770024194 |
| positive\_regulation\_of\_monocyte\_aggregation | 0.000440511814719415 | 0.022085088918651 | 0.000118948548338394 |
| positive\_regulation\_of\_hemoglobin\_biosynthetic\_process | 0.000440511814719415 | 0.000364743732317198 | 0.0125655770024194 |
| positive\_regulation\_of\_dipeptide\_transmembrane\_transport | 0.000440511814719415 | 0.000364743732317198 | 0.0125655770024194 |
| positive\_regulation\_of\_cytokine\_secretion\_involved\_in\_immune\_response | 0.000440511814719415 | 0.022085088918651 | 0.000118948548338394 |
| positive\_regulation\_of\_cGMP\_metabolic\_process | 0.000440511814719415 | 0.022085088918651 | 0.000118948548338394 |
| positive\_regulation\_of\_cellular\_biosynthetic\_process | 0.000440511814719415 | 0.022085088918651 | 0.000118948548338394 |
| plasma\_membrane\_part | 0.000440511814719415 | 0.000364743732317198 | 0.0125655770024194 |
| oxysterol\_7-alpha-hydroxylase\_activity | 0.000440511814719415 | 0.022085088918651 | 0.000118948548338394 |
| optic\_cup\_formation\_involved\_in\_camera-type\_eye\_development | 0.000440511814719415 | 0.022085088918651 | 0.000118948548338394 |
| negative\_regulation\_of\_muscle\_hyperplasia | 0.000440511814719415 | 0.000364743732317198 | 0.0125655770024194 |
| negative\_regulation\_of\_metanephric\_nephron\_tubule\_epithelial\_cell\_differentiation | 0.000440511814719415 | 0.000364743732317198 | 0.000118948548338394 |
| negative\_regulation\_of\_chemokine\_\_C-X-C\_motif\_\_ligand\_2\_production | 0.000440511814719415 | 0.000364743732317198 | 0.0125655770024194 |
| negative\_regulation\_of\_bone\_remodeling | 0.000440511814719415 | 0.000364743732317198 | 0.0125655770024194 |
| myelin\_maintenance | 0.000440511814719415 | 0.000364743732317198 | 0.0125655770024194 |
| male\_meiosis\_chromosome\_segregation | 0.000440511814719415 | 0.000364743732317198 | 0.0125655770024194 |
| maintenance\_of\_centrosome\_location | 0.000440511814719415 | 0.022085088918651 | 0.000118948548338394 |
| lateral\_sprouting\_involved\_in\_mammary\_gland\_duct\_morphogenesis | 0.000440511814719415 | 0.022085088918651 | 0.000118948548338394 |
| intracellular\_canaliculus | 0.000440511814719415 | 0.000364743732317198 | 0.0125655770024194 |
| inhibin\_A\_complex | 0.000440511814719415 | 0.022085088918651 | 0.000118948548338394 |
| hypophysis\_morphogenesis | 0.000440511814719415 | 0.022085088918651 | 0.000118948548338394 |
| histone\_kinase\_activity | 0.000440511814719415 | 0.022085088918651 | 0.000118948548338394 |
| hepatic\_immune\_response | 0.000440511814719415 | 0.000364743732317198 | 0.0125655770024194 |
| glycosylceramidase\_activity | 0.000440511814719415 | 0.000364743732317198 | 0.0125655770024194 |
| geranylgeranyl\_reductase\_activity | 0.000440511814719415 | 0.000364743732317198 | 0.0125655770024194 |
| frizzled-2\_binding | 0.000440511814719415 | 0.022085088918651 | 0.000118948548338394 |
| forebrain\_neuroblast\_division | 0.000440511814719415 | 0.022085088918651 | 0.000118948548338394 |
| farnesol\_catabolic\_process | 0.000440511814719415 | 0.000364743732317198 | 0.0125655770024194 |
| endothelin\_receptor\_activity | 0.000440511814719415 | 0.022085088918651 | 0.000118948548338394 |
| DNA\_clamp\_loader\_activity | 0.000440511814719415 | 0.022085088918651 | 0.000118948548338394 |
| cysteine-type\_endopeptidase\_regulator\_activity\_involved\_in\_apoptotic\_process | 0.000440511814719415 | 0.022085088918651 | 0.000118948548338394 |
| CXCR\_chemokine\_receptor\_binding | 0.000440511814719415 | 0.000364743732317198 | 0.0125655770024194 |
| convergent\_extension\_involved\_in\_organogenesis | 0.000440511814719415 | 0.022085088918651 | 0.000118948548338394 |
| collagen\_type\_XI | 0.000440511814719415 | 0.022085088918651 | 0.000118948548338394 |
| cervix\_development | 0.000440511814719415 | 0.022085088918651 | 0.000118948548338394 |
| angiotensin-mediated\_signaling\_pathway | 0.000440511814719415 | 0.000364743732317198 | 0.0125655770024194 |
| 17-alpha\_20-alpha-dihydroxypregn-4-en-3-one\_dehydrogenase\_activity | 0.000440511814719415 | 0.000364743732317198 | 0.0125655770024194 |
| taurine\_transport | 0 | 0.0137238830109637 | 0 |
| taurine:sodium\_symporter\_activity | 0 | 0.0137238830109637 | 0 |
| sucrose\_alpha-glucosidase\_activity | 0 | 0 | 0.00773538987579499 |
| stromal-epithelial\_cell\_signaling\_involved\_in\_prostate\_gland\_development | 0 | 0 | 0.00773538987579499 |
| steroid\_7-alpha-hydroxylase\_activity | 0 | 0.0137238830109637 | 0 |
| sequence-specific\_DNA\_binding\_transcription\_factor\_recruiting\_transcription\_factor\_activity | 0 | 0 | 0.00773538987579499 |
| septin\_ring\_assembly | 0 | 0.0137238830109637 | 0 |
| RNA\_polymerase\_II\_core\_promoter\_sequence-specific\_DNA\_binding\_transcription\_factor\_activity\_involved\_in\_preinitiation\_complex\_assembly | 0 | 0.0137238830109637 | 0 |
| regulation\_of\_Schwann\_cell\_differentiation | 0 | 0.0137238830109637 | 0 |
| regulation\_of\_follicle-stimulating\_hormone\_secretion | 0 | 0.0137238830109637 | 0 |
| receptor\_tyrosine\_kinase-like\_orphan\_receptor\_binding | 0 | 0.0137238830109637 | 0 |
| racemase\_and\_epimerase\_activity | 0 | 0 | 0.00773538987579499 |
| pronuclear\_fusion | 0 | 0.0137238830109637 | 0 |
| positive\_regulation\_of\_plasma\_membrane\_long-chain\_fatty\_acid\_transport | 0 | 0.0137238830109637 | 0 |
| positive\_regulation\_of\_IP-10\_production | 0 | 0 | 0.00773538987579499 |
| positive\_regulation\_of\_activation\_of\_Janus\_kinase\_activity | 0 | 0 | 0.00773538987579499 |
| planar\_cell\_polarity\_pathway\_involved\_in\_ventricular\_septum\_morphogenesis | 0 | 0.0137238830109637 | 0 |
| planar\_cell\_polarity\_pathway\_involved\_in\_pericardium\_morphogenesis | 0 | 0.0137238830109637 | 0 |
| planar\_cell\_polarity\_pathway\_involved\_in\_outflow\_tract\_morphogenesis | 0 | 0.0137238830109637 | 0 |
| planar\_cell\_polarity\_pathway\_involved\_in\_cardiac\_right\_atrium\_morphogenesis | 0 | 0.0137238830109637 | 0 |
| planar\_cell\_polarity\_pathway\_involved\_in\_cardiac\_muscle\_tissue\_morphogenesis | 0 | 0.0137238830109637 | 0 |
| peptidyl-proline\_hydroxylation | 0 | 0 | 0.00773538987579499 |
| outer\_mucus\_layer | 0 | 0 | 0.00773538987579499 |
| oligo-1\_6-glucosidase\_activity | 0 | 0 | 0.00773538987579499 |
| negative\_regulation\_of\_mitochondrial\_membrane\_potential | 0 | 0.0137238830109637 | 0 |
| negative\_regulation\_of\_canonical\_Wnt\_receptor\_signaling\_pathway\_involved\_in\_controlling\_type\_B\_pancreatic\_cell\_proliferation | 0 | 0 | 0.00773538987579499 |
| negative\_regulation\_of\_asymmetric\_cell\_division | 0 | 0.0137238830109637 | 0 |
| lipoprotein\_particle\_receptor\_activity | 0 | 0 | 0.00773538987579499 |
| interleukin-6\_binding | 0 | 0 | 0.00773538987579499 |
| inner\_mucus\_layer | 0 | 0 | 0.00773538987579499 |
| histamine\_uptake | 0 | 0.0137238830109637 | 0 |
| guanylate\_cyclase\_activator\_activity | 0 | 0 | 0.00773538987579499 |
| guanosine-diphosphatase\_activity | 0 | 0 | 0.00773538987579499 |
| cortical\_cytoskeleton\_organization | 0 | 0 | 0.00773538987579499 |
| convergent\_extension\_involved\_in\_somitogenesis | 0 | 0 | 0.00773538987579499 |
| ciliary\_neurotrophic\_factor\_binding | 0 | 0 | 0.00773538987579499 |
| antibacterial\_peptide\_secretion | 0 | 0.0137238830109637 | 0 |
| activin\_A\_complex | 0 | 0.0137238830109637 | 0 |
| 24-hydroxycholesterol\_7alpha-hydroxylase\_activity | 0 | 0.0137238830109637 | 0 |
