# Supplementary material for: Inferring active regulatory networks from gene expression data using a combination of prior knowledge and enrichment analysis
Source: BMC Bioinformatics. 2016 Jun 6;17(Suppl 5):181. doi: 10.1186/s12859-016-1040-7 (PMC4905609; doi:10.1186/s12859-016-1040-7)
Supplement: Additional file 1: — Human Test case results. Additional file 1 is a folder containing the detailed results of the Human Test case in HTML format. Each file includes the respective calculated enrichments for TFs, miRNAs, KEGG pathways, KEGG pathway categories and GO terms. In order to view the results a standard web-browser is needed (Chrome and Mozilla Firefox have been tested). The HTML must be opened from inside the folder because additional files (images and javascripts) which are needed for the correct view of the results are included. (ZIP 90 kb) [file 12859_2016_1040_MOESM1_ESM.zip › AdditionalFiles1/GSE21510 KEGG_Enrichment.html]

GSE21510 KEGG\_Enrichment


| KEGG\_pathway | DE\_qvalue | UP\_qvalue | DOWN\_qvalue |
| --- | --- | --- | --- |
| Olfactory\_transduction | 0.536503985661205 | 0.32081929436854 | 0.640828599797216 |
| PI3K-Akt\_signaling\_pathway | 0.433375206971586 | 0.478645473185408 | 0.234143530594419 |
| Pathways\_in\_cancer | 0.0661410169768712 | 0.237574143395063 | 0.0212709889584616 |
| MicroRNAs\_in\_cancer | 0.604693131994418 | 0.731383826399474 | 0.200241466715209 |
| Neuroactive\_ligand-receptor\_interaction | 0.567417194101941 | 0.709695723788925 | 0.194862368458474 |
| Cytokine-cytokine\_receptor\_interaction | 0.150798855515777 | 0.161438091427241 | 0.19114813090666 |
| HTLV-I\_infection | 0.0838354238785552 | 0.160741386738488 | 0.0783007492663846 |
| MAPK\_signaling\_pathway | 0.278962511670933 | 0.362658173367108 | 0.186403759075717 |
| Proteoglycans\_in\_cancer | 0.223023067707998 | 0.643850927067096 | 0.0616935941408651 |
| Viral\_carcinogenesis | 0.101669535960123 | 0.290959200497393 | 0.0550559836115515 |
| Focal\_adhesion | 0.428191796203563 | 0.614384220278486 | 0.154840218224832 |
| Epstein-Barr\_virus\_infection | 0.190629491900424 | 0.610466217811249 | 0.0529751550126741 |
| Chemokine\_signaling\_pathway | 0.171246644799898 | 0.0948710560110525 | 0.142597528813279 |
| Herpes\_simplex\_infection | 0.0858132504506432 | 0.093017943966144 | 0.0514258218269157 |
| Calcium\_signaling\_pathway | 0.163377735884079 | 0.256140990219579 | 0.138243252652511 |
| Tuberculosis | 0.160904699317157 | 0.0884065460919383 | 0.135155231048301 |
| Transcriptional\_misregulation\_in\_cancer | 0.363303701791792 | 0.571827007090286 | 0.135155231048301 |
| Influenza\_A | 0.160226891678962 | 0.0865766011159987 | 0.135155231048301 |
| Alzheimers\_disease | 0.342872015433478 | 0.236926266598232 | 0.350826161516019 |
| Purine\_metabolism | 0.150798855515777 | 0.0841923397494007 | 0.350518600414478 |
| Jak-STAT\_signaling\_pathway | 0.133203536843479 | 0.0786937839841054 | 0.119375272631817 |
| Hippo\_signaling\_pathway | 0.133203536843479 | 0.525627284088336 | 0.0423511756852458 |
| Hepatitis\_B | 0.128362926459122 | 0.072244937585426 | 0.111367972882882 |
| Cell\_adhesion\_molecules\_(CAMs) | 0.00580436326242461 | 0.0200362914596009 | 0.0373832132699543 |
| Wnt\_signaling\_pathway | 0.0234274920176243 | 0.068150853864425 | 0.0373832132699543 |
| Natural\_killer\_cell\_mediated\_cytotoxicity | 0.256762219589012 | 0.172196105777224 | 0.297045266938389 |
| Ribosome | 0.256397481186595 | 0.478645473185408 | 0.0974256427900116 |
| Tight\_junction | 0.0054752765855538 | 0.00411705421808018 | 0.0974256427900116 |
| Measles | 0.114752893124808 | 0.0658528636915222 | 0.0974256427900116 |
| Osteoclast\_differentiation | 0.114523623381958 | 0.0658528636915222 | 0.0974256427900116 |
| Hepatitis\_C | 0.00109271737692475 | 0.000633720472958783 | 0.0373832132699543 |
| Axon\_guidance | 0.0440978470382197 | 0.0156820863636108 | 0.286580923966677 |
| Cell\_cycle | 0.0440525910765499 | 0.153461982139991 | 0.0361328294439108 |
| Lysosome | 0.223023067707998 | 0.151268107275506 | 0.275699060519146 |
| Vascular\_smooth\_muscle\_contraction | 0.223023067707998 | 0.448946332081863 | 0.0880967720460038 |
| Toxoplasmosis | 0.218248813592703 | 0.148572647329735 | 0.0877427861001644 |
| Leukocyte\_transendothelial\_migration | 0.0032071225743538 | 0.00293165192149718 | 0.0877427861001644 |
| Glutamatergic\_synapse | 0.217550037078259 | 0.148572647329735 | 0.268351205119649 |
| Oocyte\_meiosis | 0.033462567366136 | 0.139932926364176 | 0.0325449431120199 |
| TNF\_signaling\_pathway | 0.199917311238421 | 0.421137342995969 | 0.0805982080906712 |
| Amoebiasis | 0.0864354303072592 | 0.135465796266456 | 0.0805982080906712 |
| Toll-like\_receptor\_signaling\_pathway | 0.190629491900424 | 0.130967277518923 | 0.0787928917887994 |
| T\_cell\_receptor\_signaling\_pathway | 0.190629491900424 | 0.130967277518923 | 0.248528196703566 |
| HIF-1\_signaling\_pathway | 0.190629491900424 | 0.130967277518923 | 0.248528196703566 |
| Pyrimidine\_metabolism | 0.190629491900424 | 0.130967277518923 | 0.248528196703566 |
| Melanogenesis | 0.18591892771926 | 0.397493259012168 | 0.0755836103318766 |
| Pancreatic\_secretion | 0.00122488835719268 | 0.000122032482278226 | 0.234143530594419 |
| NF-kappa\_B\_signaling\_pathway | 0.162433293866177 | 0.108092503429292 | 0.227520521082148 |
| mRNA\_surveillance\_pathway | 0.162433293866177 | 0.368983041152953 | 0.0638238189490555 |
| Rheumatoid\_arthritis | 0.0234274920176243 | 0.105545478632839 | 0.0212709889584616 |
| Gap\_junction | 0.160904699317157 | 0.36583642352093 | 0.0627974728516016 |
| Protein\_digestion\_and\_absorption | 0.160726885328018 | 0.36583642352093 | 0.0627974728516016 |
| Hematopoietic\_cell\_lineage | 0.0234274920176243 | 0.0288965212917869 | 0.0627974728516016 |
| Apoptosis | 0.160726885328018 | 0.105078838948004 | 0.222927953103232 |
| Small\_cell\_lung\_cancer | 0.160190310640412 | 0.102493765933026 | 0.22049999632582 |
| Progesterone-mediated\_oocyte\_maturation | 0.0609637954619455 | 0.362658173367108 | 0.0212709889584616 |
| ECM-receptor\_interaction | 0.0234274920176243 | 0.362658173367108 | 0.00746125667700299 |
| Chemical\_carcinogenesis | 0.0551489425521743 | 0.0261622501851873 | 0.213318155374281 |
| TGF-beta\_signaling\_pathway | 0.0529949379263532 | 0.093067958580216 | 0.0581031210795035 |
| Peroxisome | 0.147267084929086 | 0.351892257227003 | 0.0581031210795035 |
| Metabolism\_of\_xenobiotics\_by\_cytochrome\_P450 | 0.0529949379263532 | 0.0258858516190392 | 0.210677116826155 |
| Gastric\_acid\_secretion | 0.133767756310592 | 0.0871699393290296 | 0.200678165703789 |
| B\_cell\_receptor\_signaling\_pathway | 0.133203536843479 | 0.0865766011159987 | 0.200241466715209 |
| Drug\_metabolism\_-\_cytochrome\_P450 | 0.0449881451801699 | 0.0209050315614035 | 0.200241466715209 |
| Prolactin\_signaling\_pathway | 0.131227676744428 | 0.0853863324585542 | 0.0529751550126741 |
| Leishmaniasis | 0.131227676744428 | 0.0853863324585542 | 0.0529751550126741 |
| Bile\_secretion | 0.000590594583950853 | 4.47710532705364e-05 | 0.200241466715209 |
| RIG-I-like\_receptor\_signaling\_pathway | 0.0442084822390539 | 0.0202112809393074 | 0.200241466715209 |
| PPAR\_signaling\_pathway | 0.0442084822390539 | 0.321414553309878 | 0.0212709889584616 |
| Amphetamine\_addiction | 0.128362926459122 | 0.0841923397494007 | 0.200241466715209 |
| Adipocytokine\_signaling\_pathway | 0.128362926459122 | 0.32081929436854 | 0.0529751550126741 |
| Long-term\_potentiation | 0.128362926459122 | 0.0841923397494007 | 0.199949287072179 |
| Retinol\_metabolism | 0.0440525910765499 | 0.0196802131387929 | 0.199431915169579 |
| p53\_signaling\_pathway | 0.0440525910765499 | 0.319538571065384 | 0.0212709889584616 |
| Pancreatic\_cancer | 0.122461071660349 | 0.0801633713050357 | 0.0515320652176029 |
| Glycolysis\_/\_Gluconeogenesis | 0.122461071660349 | 0.0801633713050357 | 0.196162705457469 |
| Inflammatory\_bowel\_disease\_(IBD) | 0.122461071660349 | 0.0801633713050357 | 0.0515320652176029 |
| VEGF\_signaling\_pathway | 0.117880922953355 | 0.0786937839841054 | 0.192143836547403 |
| Cytosolic\_DNA-sensing\_pathway | 0.116324428033894 | 0.0786937839841054 | 0.19147232551866 |
| Shigellosis | 0.114752893124808 | 0.29395048227921 | 0.0498549119541712 |
| Arginine\_and\_proline\_metabolism | 0.106622180377683 | 0.0711384927487763 | 0.183341752050774 |
| Steroid\_hormone\_biosynthesis | 0.0314099141983973 | 0.0133402933108733 | 0.18248891525679 |
| Starch\_and\_sucrose\_metabolism | 0.0314099141983973 | 0.0133402933108733 | 0.18248891525679 |
| Pathogenic\_Escherichia\_coli\_infection | 0.101669535960123 | 0.274221967802205 | 0.0426202107176676 |
| Glycerolipid\_metabolism | 0.101669535960123 | 0.068150853864425 | 0.18248891525679 |
| Basal\_cell\_carcinoma | 0.101669535960123 | 0.274221967802205 | 0.0426202107176676 |
| Vibrio\_cholerae\_infection | 0.101669535960123 | 0.068150853864425 | 0.18248891525679 |
| Non-small\_cell\_lung\_cancer | 0.101669535960123 | 0.274221967802205 | 0.0426202107176676 |
| Taste\_transduction | 0.0301884663935713 | 0.0133402933108733 | 0.18200434546553 |
| Amyotrophic\_lateral\_sclerosis\_(ALS) | 0.101669535960123 | 0.068150853864425 | 0.18200434546553 |
| Ovarian\_steroidogenesis | 0.0301884663935713 | 0.0132776889722719 | 0.180060264116124 |
| Mineral\_absorption | 0.00580436326242461 | 0.00146351490284346 | 0.180060264116124 |
| Hedgehog\_signaling\_pathway | 0.0999449660606687 | 0.265068182154007 | 0.0423511756852458 |
| Glutathione\_metabolism | 0.0301884663935713 | 0.0132776889722719 | 0.180060264116124 |
| Nucleotide\_excision\_repair | 0.0878118663378974 | 0.252009371247716 | 0.0401521391809736 |
| Intestinal\_immune\_network\_for\_IgA\_production | 0.0878118663378974 | 0.0592038436042103 | 0.173410030800506 |
| Carbohydrate\_digestion\_and\_absorption | 0.0858132504506432 | 0.0560862266009731 | 0.168681436574295 |
| Fatty\_acid\_degradation | 0.0234274920176243 | 0.0553264881041659 | 0.0373832132699543 |
| ABC\_transporters | 0.0856567168210734 | 0.0553264881041659 | 0.167394450650631 |
| Pyruvate\_metabolism | 0.0783741474832335 | 0.051390485067848 | 0.158722337082114 |
| Tyrosine\_metabolism | 0.0729736319342774 | 0.0482106248353905 | 0.154840218224832 |
| Aldosterone-regulated\_sodium\_reabsorption | 0.0234274920176243 | 0.00715398042048816 | 0.154840218224832 |
| Bladder\_cancer | 0.0710505484767315 | 0.215088721667652 | 0.0366781501246766 |
| Fructose\_and\_mannose\_metabolism | 0.0661410169768712 | 0.0427178892563168 | 0.148460216906333 |
| DNA\_replication | 0.0661410169768712 | 0.20710184448536 | 0.0361328294439108 |
| Pentose\_and\_glucuronate\_interconversions | 0.063210857151217 | 0.0395755482699444 | 0.142650718845439 |
| Galactose\_metabolism | 0.0119036959963488 | 0.00411705421808018 | 0.135155231048301 |
| Nitrogen\_metabolism | 0.00011835588763755 | 1.29803239559268e-05 | 0.127577907885947 |
| Collecting\_duct\_acid\_secretion | 0.0463142233749972 | 0.0271312063060284 | 0.127577907885947 |
| Glycosphingolipid\_biosynthesis\_-\_lacto\_and\_neolacto\_series | 0.0449881451801699 | 0.0261622501851873 | 0.127243825064458 |
| Glycosaminoglycan\_biosynthesis\_-\_heparan\_sulfate\_/\_heparin | 0.0440525910765499 | 0.151268107275506 | 0.0212709889584616 |
| Proximal\_tubule\_bicarbonate\_reclamation | 0.000661819377922396 | 0.000113970857899287 | 0.117008546790148 |
| Mismatch\_repair | 0.0440525910765499 | 0.148572647329735 | 0.0212709889584616 |
| Renin-angiotensin\_system | 0.0301884663935713 | 0.0156820863636108 | 0.0974256427900116 |
| Primary\_bile\_acid\_biosynthesis | 0.0301884663935713 | 0.126184190908253 | 0.0212709889584616 |
| Glycosaminoglycan\_biosynthesis\_-\_keratan\_sulfate | 0.0300499497687544 | 0.0133402933108733 | 0.0880967720460038 |
| Cyanoamino\_acid\_metabolism | 0.0112144106571302 | 0.00464098368075833 | 0.0581031210795035 |
