# Supplementary material for: Inferring active regulatory networks from gene expression data using a combination of prior knowledge and enrichment analysis
Source: BMC Bioinformatics. 2016 Jun 6;17(Suppl 5):181. doi: 10.1186/s12859-016-1040-7 (PMC4905609; doi:10.1186/s12859-016-1040-7)
Supplement: Additional file 1: — Human Test case results. Additional file 1 is a folder containing the detailed results of the Human Test case in HTML format. Each file includes the respective calculated enrichments for TFs, miRNAs, KEGG pathways, KEGG pathway categories and GO terms. In order to view the results a standard web-browser is needed (Chrome and Mozilla Firefox have been tested). The HTML must be opened from inside the folder because additional files (images and javascripts) which are needed for the correct view of the results are included. (ZIP 90 kb) [file 12859_2016_1040_MOESM1_ESM.zip › AdditionalFiles1/GSE21510 miRNA_Enrichment.html]

GSE21510 miRNA\_Enrichment


| miRNA | DE\_qvalue | UP\_qvalue | DOWN\_qvalue |
| --- | --- | --- | --- |
| hsa-miR-19b-3p | 0.0979997886641242 | 0.288367688692278 | 0.0278966549675804 |
| hsa-miR-186-5p | 0.0207392615885291 | 0.279458894093039 | 0.0017642008871436 |
| hsa-miR-590-3p | 0.0519314607022577 | 0.272360853330287 | 0.0156303389992556 |
| hsa-miR-18a-5p | 0.04300898028382 | 0.0312851225957001 | 0.0954083477878016 |
| hsa-miR-26b-5p | 0.0333561357833763 | 0.0287017065112519 | 0.0872115521987371 |
| hsa-miR-374b-5p | 0.0184094733995804 | 0.0154623162916424 | 0.0550108892425343 |
| hsa-miR-140-3p | 0.0184094733995804 | 0.0975387234662267 | 0.0017642008871436 |
