# Supplementary material for: Inferring active regulatory networks from gene expression data using a combination of prior knowledge and enrichment analysis
Source: BMC Bioinformatics. 2016 Jun 6;17(Suppl 5):181. doi: 10.1186/s12859-016-1040-7 (PMC4905609; doi:10.1186/s12859-016-1040-7)
Supplement: Additional file 2: — Mouse Test case results. Additional file 2 is a folder containing the detailed results of the Mouse Test case in HTML format. Each file includes the respective calculated enrichments for TFs, miRNAs, KEGG pathways, KEGG pathway categories and GO terms. In order to view the results a standard web-browser is needed (Chrome and Mozilla Firefox have been tested). The HTML files must be opened from inside the folder because additional files (images and javascripts) which are needed for the correct view of the results are included. (ZIP 83 kb) [file 12859_2016_1040_MOESM2_ESM.zip › AdditionalFiles2/GSE63889 GO_Enrichment.html]

GSE63889 GO\_Enrichment


| GO | DE\_qvalue | UP\_qvalue | DOWN\_qvalue |
| --- | --- | --- | --- |
| protein\_binding | 0.00267007180139474 | 0.00595001832942766 | 0.0905180123235094 |
| integral\_to\_membrane | 0.827100836610627 | 0.87939227636959 | 0.277412824017079 |
| nucleus | 0.00200999030204701 | 0.00248938642379043 | 0.172101476620091 |
| cytoplasm | 0.000617243188560436 | 0.0013525845021205 | 0.0630344628670297 |
| membrane | 0.143317032733575 | 0.220224570876719 | 0.119990212287211 |
| plasma\_membrane | 0.0198607309151839 | 0.0380545297111451 | 0.0783092864502495 |
| metal\_ion\_binding | 0.531160056262239 | 0.48785047034921 | 0.429223536553318 |
| G\_protein\_coupled\_receptor\_protein\_signaling\_pathway | 0.977142376172775 | 0.947239670537419 | 0.751873989291282 |
| intracellular | 0.403894682785217 | 0.367551042527699 | 0.381956465286656 |
| receptor\_activity | 0.929778535811724 | 0.862352135326566 | 0.737592577403318 |
| zinc\_ion\_binding | 0.384173777460914 | 0.349559224516502 | 0.37510941921625 |
| regulation\_of\_transcription\_DNA\_dependent | 0.253249375654038 | 0.329118805177987 | 0.136871436400756 |
| nucleotide\_binding | 0.78615524664505 | 0.791879206030098 | 0.330162586844396 |
| DNA\_binding | 0.185592136113791 | 0.0988239073730835 | 0.664873454593148 |
| ATP\_binding | 0.226290767968259 | 0.219218645662303 | 0.268517779384702 |
| extracellular\_region | 0.000909547843209255 | 0.000597494840585014 | 0.260513155087283 |
| mitochondrion | 0.811603400340705 | 0.849128798429879 | 0.231561589761005 |
| cytosol | 0.00020260805991806 | 0.000130479915942299 | 0.205652175768121 |
| nucleic\_acid\_binding | 0.840541433170217 | 0.759492002917693 | 0.547040237676599 |
| binding | 0.186298711726726 | 0.208104285332556 | 0.16878089291044 |
| sequence\_specific\_DNA\_binding\_transcription\_factor\_activity | 0.0381910792454273 | 0.0194682647099927 | 0.447078935038358 |
| hydrolase\_activity | 0.143266729917233 | 0.087404841683401 | 0.43603957109465 |
| transferase\_activity | 0.5996714960364 | 0.497113538875062 | 0.429223536553318 |
| endoplasmic\_reticulum | 0.237207270824371 | 0.161640190829726 | 0.426588941543621 |
| Golgi\_apparatus | 0.530609787802629 | 0.653131379548784 | 0.0992158802974085 |
| signal\_transduction | 0.00098702062735531 | 0.00841370118968554 | 0.0085735738089241 |
| catalytic\_activity | 0.186248467068226 | 0.242584074298747 | 0.0981432770592688 |
| multicellular\_organismal\_development | 0.293019159850885 | 0.212530962522611 | 0.382519031610545 |
| extracellular\_space | 1.81891749176636e-07 | 1.47285047656146e-07 | 0.0887042771907696 |
| calcium\_ion\_binding | 0.469979549894223 | 0.372907889656621 | 0.378357055520895 |
| sequence\_specific\_DNA\_binding | 0.225597098569462 | 0.159326404705862 | 0.351895835071433 |
| cytoskeleton | 0.369606512369316 | 0.752952925690719 | 0.0166138796750628 |
| transport | 0.186618891025564 | 0.269354705912728 | 0.0693038514621035 |
| protein\_phosphorylation | 0.185592136113791 | 0.127559640800814 | 0.330806452265454 |
| transferase\_activity\_transferring\_phosphorus\_containing\_groups | 0.334617885679587 | 0.254687107279497 | 0.325090512190023 |
| metabolic\_process | 0.172231506482239 | 0.253183588670771 | 0.0660196246831409 |
| protein\_kinase\_activity | 0.313295590446419 | 0.236980413008881 | 0.316378127792984 |
| protein\_serine\_threonine\_kinase\_activity | 0.303707686748896 | 0.229518450101716 | 0.31266762486997 |
| transmembrane\_transport | 0.514607440632391 | 0.429209103597681 | 0.30944107082633 |
| proteolysis | 0.0518302702355523 | 0.0315599242689289 | 0.296813245133225 |
| RNA\_binding | 0.477331082415528 | 0.394253399718313 | 0.295587514918877 |
| G\_protein\_coupled\_receptor\_activity | 0.261161695715206 | 0.195513203993405 | 0.294882980304236 |
| oxidoreductase\_activity | 0.0415904995874439 | 0.070117481966354 | 0.0513209118849256 |
| protein\_transport | 0.699389151466223 | 0.630886269827279 | 0.281389138210394 |
| nucleoplasm | 0.435761407817927 | 0.356481393449764 | 0.280645968619567 |
| endoplasmic\_reticulum\_membrane | 0.0981061650220791 | 0.0648643252108628 | 0.27773739716033 |
| transcription\_DNA\_dependent | 0.221807272152813 | 0.163986997309777 | 0.277524758999365 |
| nucleolus | 0.406164369185681 | 0.329710166627003 | 0.270538656528537 |
| peptidase\_activity | 0.405171304979455 | 0.329118805177987 | 0.270311155479734 |
| protein\_homodimerization\_activity | 0.185592136113791 | 0.304886928271114 | 0.0442847341642326 |
| apoptosis | 1.9355791511522e-06 | 3.19332356895081e-06 | 0.0417334802306629 |
| cell\_differentiation | 0.0646930359021906 | 0.0423625916052674 | 0.249477367062842 |
| positive\_regulation\_of\_transcription\_from\_RNA\_polymerase\_II\_promoter | 0.0203505989532275 | 0.0121593971339812 | 0.244656435622648 |
| GTP\_binding | 0.321594251236431 | 0.255146539461098 | 0.23864265818431 |
| cell\_cycle | 0.577264714820384 | 0.50711235714365 | 0.234307117549463 |
| oxidation\_reduction\_process | 0.0496085277125474 | 0.031938034678823 | 0.232846759306173 |
| cell\_adhesion | 0.298891064955515 | 0.236210813745631 | 0.230489606394129 |
| cell\_junction | 0.562341965186632 | 0.493098770493238 | 0.229596125316324 |
| membrane\_fraction | 0.276975911410279 | 0.219172917272653 | 0.222212698206741 |
| identical\_protein\_binding | 0.01177647667156 | 0.00698772284607055 | 0.218918586040333 |
| perinuclear\_region\_of\_cytoplasm | 0.01070438912112 | 0.00633879876922211 | 0.214405167263003 |
| response\_to\_drug | 0.000235111283479796 | 0.000112657819014963 | 0.211059901742194 |
| intracellular\_protein\_transport | 0.491465221775944 | 0.42507975303327 | 0.204909369894436 |
| positive\_regulation\_of\_transcription\_DNA\_dependent | 0.0069406263228523 | 0.00403309322659843 | 0.19598197177319 |
| soluble\_fraction | 0.00166985020981721 | 0.000874389007152608 | 0.190043839580749 |
| intracellular\_signal\_transduction | 0.0694311108050118 | 0.0485276921229971 | 0.188412000192676 |
| positive\_regulation\_of\_cell\_proliferation | 0.000115305842427054 | 4.48223428413853e-05 | 0.186148477777618 |
| cell\_surface | 0.00131553477532275 | 0.000682703887127045 | 0.181991049449637 |
| ligase\_activity | 0.414947333134014 | 0.713486323483603 | 0.025052502684243 |
| nucleoside\_triphosphatase\_activity | 0.399609952912921 | 0.701460254809859 | 0.0238311179819602 |
| integral\_to\_plasma\_membrane | 0.0524457355253556 | 0.0363291305326193 | 0.172101476620091 |
| protein\_heterodimerization\_activity | 0.0518867698317181 | 0.121469712639439 | 0.023447172722607 |
| protein\_complex | 0.0502720845449279 | 0.0347117999869782 | 0.16997207852491 |
| negative\_regulation\_of\_transcription\_DNA\_dependent | 0.0480004639170499 | 0.033037516257983 | 0.167825363557628 |
| transporter\_activity | 0.0448466477750663 | 0.0307449057177595 | 0.164167651677038 |
| synapse | 0.367685809759906 | 0.309999853913887 | 0.164167651677038 |
| negative\_regulation\_of\_apoptosis | 0.000185236163022489 | 0.000110044201346957 | 0.163925380188338 |
| neuronal\_cell\_body | 0.132369553223988 | 0.0994811929414594 | 0.158928749774293 |
| microsome | 0.040054768390711 | 0.0276291592603532 | 0.158928749774293 |
| spermatogenesis | 0.348193071338358 | 0.292877924365542 | 0.158668691212916 |
| actin\_binding | 0.127460803489325 | 0.0961166143572767 | 0.156899912038145 |
| negative\_regulation\_of\_transcription\_from\_RNA\_polymerase\_II\_promoter | 0.340574906074696 | 0.286062996056302 | 0.156437755302325 |
| cytoplasmic\_vesicle | 0.338965182223877 | 0.284610346198367 | 0.155974010714525 |
| GTPase\_activity | 0.335330482062171 | 0.281358053174344 | 0.154848895177556 |
| signal\_transducer\_activity | 0.320267556098853 | 0.267629035037336 | 0.149741025646141 |
| negative\_regulation\_of\_cell\_proliferation | 0.00198312550428757 | 0.00109690953630837 | 0.149741025646141 |
| immune\_response | 2.05993937121785e-07 | 5.61085895576161e-08 | 0.147447271394474 |
| endosome | 0.104455028430676 | 0.254459856508078 | 0.0185932226478747 |
| iron\_ion\_binding | 0.103419131814445 | 0.0775825527639422 | 0.144282813702694 |
| ubiquitin\_protein\_ligase\_activity | 0.00670591768791471 | 0.01938028132716 | 0.0182857313349534 |
| receptor\_binding | 0.0989222908733807 | 0.0741754717985182 | 0.141772955314096 |
| dendrite | 0.276975911410279 | 0.230652838980763 | 0.136033650585665 |
| serine\_type\_endopeptidase\_activity | 0.275509813332448 | 0.229405068586079 | 0.135524200732744 |
| chromatin\_binding | 0.271703223469366 | 0.226069221675139 | 0.13433090267928 |
| transcription\_factor\_complex | 0.261677410024059 | 0.218384471444182 | 0.131078387144135 |
| extracellular\_matrix | 0.261677410024059 | 0.218384471444182 | 0.131078387144135 |
| response\_to\_hypoxia | 5.2213338493575e-06 | 1.9242129793512e-06 | 0.129345691240579 |
| electron\_carrier\_activity | 0.252556287642669 | 0.210131672708006 | 0.128129373572075 |
| in\_utero\_embryonic\_development | 0.0725621983098562 | 0.0543491581631706 | 0.125522856165643 |
| heme\_binding | 0.0698027118252853 | 0.0523167079678021 | 0.123597927706364 |
| lipid\_metabolic\_process | 0.0690862710285634 | 0.196471087760612 | 0.0157717379608665 |
| external\_side\_of\_plasma\_membrane | 2.90494097571637e-05 | 0.000101320689870711 | 0.015690849198784 |
| magnesium\_ion\_binding | 0.228281458206937 | 0.189777250945931 | 0.120267716340098 |
| intracellular\_membrane\_bounded\_organelle | 0.226492285332887 | 0.188221403406009 | 0.119873154845037 |
| centrosome | 0.226492285332887 | 0.188221403406009 | 0.119873154845037 |
| positive\_regulation\_of\_apoptosis | 0.00313470874158206 | 0.00194179226316956 | 0.11947611196973 |
| transcription\_factor\_binding | 0.221651236330385 | 0.183320644427482 | 0.118219812968053 |
| protein\_domain\_specific\_binding | 0.219848684300721 | 0.181762619137299 | 0.117664939629527 |
| protein\_kinase\_binding | 0.213901659365132 | 0.176644616928399 | 0.115691909293424 |
| protein\_ubiquitination | 0.0580266520982622 | 0.0430700221553976 | 0.115130482995723 |
| kinase\_activity | 0.212094384664198 | 0.175087133521854 | 0.115130482995723 |
| heart\_development | 0.210285183762605 | 0.173529127815774 | 0.114714400163939 |
| response\_to\_organic\_cyclic\_compound | 1.07417842369394e-07 | 3.16072326582753e-08 | 0.111290081633804 |
| proteinaceous\_extracellular\_matrix | 0.200221887675545 | 0.165113721965602 | 0.111290081633804 |
| brain\_development | 0.192255234366735 | 0.158895475659859 | 0.108703742304899 |
| regulation\_of\_apoptosis | 8.11566508768795e-05 | 4.00606338404926e-05 | 0.106681211501063 |
| inflammatory\_response | 4.54505905763631e-13 | 1.96099701291679e-12 | 0.0134580099155087 |
| enzyme\_binding | 0.00994297896639581 | 0.00675560989409346 | 0.106681211501063 |
| response\_to\_lipopolysaccharide | 8.59808314607286e-07 | 2.93142365213032e-07 | 0.10623376491972 |
| growth\_factor\_activity | 0.00186863935406084 | 0.00113262528608225 | 0.104920580129192 |
| cytokine\_activity | 7.11167632250094e-05 | 0.000230431754347797 | 0.013118571060022 |
| induction\_of\_apoptosis | 0.043845862388335 | 0.032301918985676 | 0.103602048489352 |
| lysosome | 0.17347193548945 | 0.141756749887812 | 0.102278146730544 |
| cell\_proliferation | 0.00830120251984664 | 0.00566956785972393 | 0.101680329812564 |
| postsynaptic\_membrane | 0.165820473688266 | 0.135123830949079 | 0.0996141391566429 |
| lipid\_binding | 0.165820473688266 | 0.135123830949079 | 0.0996141391566429 |
| ATPase\_activity | 0.160207893171225 | 0.457309551062938 | 0.0117234587913352 |
| helicase\_activity | 0.156411286558377 | 0.127529784000755 | 0.0964427304850815 |
| anti\_apoptosis | 3.7784147864721e-05 | 1.82470363559707e-05 | 0.094344293993874 |
| response\_to\_stress | 0.146699721614257 | 0.119526680950145 | 0.092980511863029 |
| protein\_complex\_binding | 0.0328183237138386 | 0.0243931529554277 | 0.092980511863029 |
| membrane\_raft | 0.00114256992490546 | 0.000686196776369375 | 0.092980511863029 |
| metalloendopeptidase\_activity | 0.144917498040351 | 0.118029179363806 | 0.0926009407615333 |
| guanyl\_nucleotide\_exchange\_factor\_activity | 0.144917498040351 | 0.118029179363806 | 0.0926009407615333 |
| basolateral\_plasma\_membrane | 0.0322575872640838 | 0.0240993937040872 | 0.0926009407615333 |
| DNA\_replication | 0.143317032733575 | 0.116682425820312 | 0.0922178537300219 |
| angiogenesis | 0.0316093841367726 | 0.0236619705101444 | 0.0922178537300219 |
| protein\_dephosphorylation | 0.00103136460248285 | 0.000621649463415858 | 0.0917092545821046 |
| aging | 0.0304633109255901 | 0.0226685218286848 | 0.0910761705656481 |
| phosphatase\_activity | 0.000990144898448322 | 0.000597494840585014 | 0.0905180123235094 |
| cell\_projection | 0.134095123038327 | 0.108683100317151 | 0.0891637479309674 |
| transmembrane\_receptor\_activity | 0.0261399584312461 | 0.102418545543117 | 0.0105168255485638 |
| regulation\_of\_cell\_proliferation | 0.00014187411301032 | 8.4289289600672e-05 | 0.0840569986091892 |
| dephosphorylation | 0.000746240147514607 | 0.000452041007091079 | 0.0840569986091892 |
| positive\_regulation\_of\_I\_kappaB\_kinase\_NF\_kappaB\_cascade | 1.26020371475841e-06 | 4.76548217097482e-07 | 0.0835149271948079 |
| defense\_response | 0.119292726619598 | 0.0967490916974641 | 0.0835149271948079 |
| response\_to\_DNA\_damage\_stimulus | 0.117562562318099 | 0.0954315217895169 | 0.0829694255280482 |
| neuron\_projection | 0.02272898931141 | 0.016903411481766 | 0.0823093867204537 |
| serine\_type\_endopeptidase\_inhibitor\_activity | 0.113968875325689 | 0.092441038963215 | 0.0816470524447962 |
| cysteine\_type\_peptidase\_activity | 0.112252665492367 | 0.0910136990040692 | 0.0809824130756894 |
| response\_to\_ethanol | 0.108694666316616 | 0.08817278960839 | 0.0795384046777033 |
| metallopeptidase\_activity | 0.108694666316616 | 0.08817278960839 | 0.0795384046777033 |
| response\_to\_estradiol\_stimulus | 1.01523438381041e-05 | 4.52673798906918e-06 | 0.0789745673673962 |
| response\_to\_oxidative\_stress | 0.105297799220184 | 0.0855730780064321 | 0.0783092864502495 |
| early\_endosome | 0.105297799220184 | 0.0855730780064321 | 0.0783092864502495 |
| endosome\_membrane | 0.0187264578476516 | 0.0139349400576228 | 0.07705059781068 |
| protein\_tyrosine\_phosphatase\_activity | 0.000519573611603086 | 0.000294035029785251 | 0.0755811422932868 |
| peptidase\_inhibitor\_activity | 0.098691611402561 | 0.0797695975030936 | 0.0755811422932868 |
| lung\_development | 0.098691611402561 | 0.0797695975030936 | 0.0755811422932868 |
| calmodulin\_binding | 0.00288864471324536 | 0.00193019118194682 | 0.0755811422932868 |
| positive\_regulation\_of\_gene\_expression | 0.0919224719247484 | 0.0741806748223734 | 0.0728233672697269 |
| response\_to\_peptide\_hormone\_stimulus | 0.0024541509783355 | 0.00164569645894773 | 0.0721278614937943 |
| response\_to\_glucocorticoid\_stimulus | 5.18426606226015e-06 | 2.34020075742478e-06 | 0.0721278614937943 |
| ATP\_dependent\_helicase\_activity | 0.0902773417120776 | 0.0729200620081347 | 0.0721278614937943 |
| visual\_perception | 0.0868944095911592 | 0.0701312410705323 | 0.070827195665596 |
| response\_to\_virus | 0.00235234226193825 | 0.00157453350585391 | 0.070827195665596 |
| protein\_autophosphorylation | 0.0868944095911592 | 0.0701312410705323 | 0.070827195665596 |
| postsynaptic\_density | 0.0868944095911592 | 0.0701312410705323 | 0.070827195665596 |
| positive\_regulation\_of\_cell\_migration | 5.54664097122625e-05 | 2.93615959353902e-05 | 0.070827195665596 |
| negative\_regulation\_of\_cell\_growth | 0.0868944095911592 | 0.0701312410705323 | 0.070827195665596 |
| response\_to\_nutrient | 0.0838733142704013 | 0.0678218998794363 | 0.0698040172874817 |
| focal\_adhesion | 0.014057246678465 | 0.0103487338439821 | 0.0698040172874817 |
| defense\_response\_to\_bacterium | 0.014057246678465 | 0.0103487338439821 | 0.0698040172874817 |
| actin\_cytoskeleton\_organization | 0.0838733142704013 | 0.0678218998794363 | 0.0698040172874817 |
| organ\_morphogenesis | 0.0823682327252854 | 0.0665805838869828 | 0.0693822182616175 |
| innate\_immune\_response | 0.00200999030204701 | 0.00976410000622662 | 0.00899082534621069 |
| response\_to\_organic\_substance | 3.08887308918026e-06 | 2.18276235831451e-05 | 0.00883608250282438 |
| anchored\_to\_membrane | 0.0773689411778704 | 0.0627136662421562 | 0.0671284374356943 |
| ubiquitin\_protein\_ligase\_binding | 0.0120436432682083 | 0.00886632921087397 | 0.0664043923683723 |
| protein\_homooligomerization | 0.0120436432682083 | 0.00886632921087397 | 0.0664043923683723 |
| protein\_dimerization\_activity | 0.00182691147164677 | 0.00121306922394493 | 0.0664043923683723 |
| stored\_secretory\_granule | 0.0117004068522328 | 0.0086897989580069 | 0.0659564525013399 |
| endocytosis | 0.0741223185716178 | 0.0600340080123212 | 0.0659564525013399 |
| symporter\_activity | 0.0725621983098562 | 0.0587477728325821 | 0.0653170412030411 |
| electron\_transport\_chain | 0.0711040578022191 | 0.0576241043825194 | 0.0645815754590816 |
| flavin\_adenine\_dinucleotide\_binding | 0.069651356762567 | 0.0563509846630281 | 0.0638434664347749 |
| extracellular\_matrix\_organization | 0.0106980182775552 | 0.00789911363806906 | 0.0638434664347749 |
| response\_to\_cytokine\_stimulus | 2.7679213127304e-05 | 1.42506778694617e-05 | 0.0631927206125767 |
| negative\_regulation\_of\_neuron\_apoptosis | 0.068294422468844 | 0.0550862970800286 | 0.0631927206125767 |
| cell\_cycle\_arrest | 0.0103264609535986 | 0.0550862970800286 | 0.0085735738089241 |
| regulation\_of\_cell\_cycle | 0.000214942174387806 | 0.000122550750531716 | 0.0626272917195941 |
| post\_embryonic\_development | 0.0651553756196447 | 0.0527254142193669 | 0.0618783559194631 |
| heat\_shock\_protein\_binding | 0.0651553756196447 | 0.0527254142193669 | 0.0618783559194631 |
| transcription\_from\_RNA\_polymerase\_II\_promoter | 0.0638147298079556 | 0.0516239952353348 | 0.0612146622671658 |
| Rho\_guanyl\_nucleotide\_exchange\_factor\_activity | 0.0623114400112842 | 0.0503884699467073 | 0.0604593342638056 |
| protein\_N\_terminus\_binding | 0.0623114400112842 | 0.0503884699467073 | 0.0604593342638056 |
| positive\_regulation\_of\_protein\_phosphorylation | 0.00904689014893337 | 0.00672152409282461 | 0.0604593342638056 |
| elevation\_of\_cytosolic\_calcium\_ion\_concentration | 0.00904689014893337 | 0.00672152409282461 | 0.0604593342638056 |
| lysosomal\_membrane | 0.0608996601595438 | 0.0492294578252151 | 0.0599608361525915 |
| chemotaxis | 1.74473931684862e-09 | 1.92552404421393e-08 | 0.0085735738089241 |
| regulation\_of\_Rho\_protein\_signal\_transduction | 0.0594155762459452 | 0.0482090960731575 | 0.0591970782777737 |
| intracellular\_protein\_kinase\_cascade | 0.0594155762459452 | 0.0482090960731575 | 0.0591970782777737 |
| endoplasmic\_reticulum\_lumen | 0.0594155762459452 | 0.0482090960731575 | 0.0591970782777737 |
| ubiquitin\_thiolesterase\_activity | 0.0580977439407584 | 0.0471255462667929 | 0.0586008858634371 |
| response\_to\_toxin | 0.0580977439407584 | 0.0471255462667929 | 0.0586008858634371 |
| double\_stranded\_DNA\_binding | 0.0580977439407584 | 0.0471255462667929 | 0.0586008858634371 |
| cell\_redox\_homeostasis | 0.0580977439407584 | 0.0471255462667929 | 0.0586008858634371 |
| trans\_Golgi\_network | 0.0538801034878148 | 0.0435974320846481 | 0.0563608977734263 |
| response\_to\_estrogen\_stimulus | 0.00093775699438799 | 0.000603616922005678 | 0.0555800202061824 |
| motor\_activity | 0.0524457355253556 | 0.0424795566268819 | 0.0555800202061824 |
| response\_to\_hormone\_stimulus | 0.0512315598742684 | 0.0413695179761447 | 0.0548769732147514 |
| cytoplasmic\_vesicle\_membrane | 0.0512315598742684 | 0.0413695179761447 | 0.0548769732147514 |
| fatty\_acid\_metabolic\_process | 0.0485463318170241 | 0.255357011946794 | 0.00753907447554064 |
| response\_to\_insulin\_stimulus | 0.000746240147514607 | 0.000530577299339252 | 0.052427670592734 |
| induction\_of\_apoptosis\_by\_extracellular\_signals | 0.047217995364811 | 0.0379818309696216 | 0.052427670592734 |
| endonuclease\_activity | 0.047217995364811 | 0.0379818309696216 | 0.052427670592734 |
| cell\_cell\_junction | 0.047217995364811 | 0.0379818309696216 | 0.052427670592734 |
| pyridoxal\_phosphate\_binding | 0.0459627123804943 | 0.0369601173800488 | 0.0518624664447142 |
| neuron\_projection\_development | 0.0459627123804943 | 0.0369601173800488 | 0.0518624664447142 |
| male\_gonad\_development | 0.0459627123804943 | 0.0369601173800488 | 0.0518624664447142 |
| cytoskeleton\_organization | 0.0447764086283099 | 0.0359957254464105 | 0.051289918095572 |
| oxidoreductase\_activity\_acting\_on\_single\_donors\_with\_incorporation\_of\_molecular\_oxygen\_\_incorporation\_of\_two\_atoms\_of\_oxygen | 0.0434738630838598 | 0.0348866078587111 | 0.0504821536147177 |
| organ\_regeneration | 0.000690130696589292 | 0.000440793059324282 | 0.0504821536147177 |
| cell\_cell\_adhesion | 0.0434738630838598 | 0.0348866078587111 | 0.0504821536147177 |
| response\_to\_cAMP | 0.000645715431330164 | 0.000415532941492481 | 0.0498207667678922 |
| wound\_healing | 0.000609716976705656 | 0.000415532941492481 | 0.0490047422004869 |
| ossification | 0.00479081412151727 | 0.0035860009900789 | 0.0490047422004869 |
| retina\_development\_in\_camera\_type\_eye | 0.0395221949710416 | 0.0318153745679297 | 0.0482584518626148 |
| positive\_regulation\_of\_NF\_kappaB\_transcription\_factor\_activity | 1.91641330579548e-07 | 8.14927941799345e-08 | 0.0482584518626148 |
| cytokine\_mediated\_signaling\_pathway | 0.0395221949710416 | 0.0318153745679297 | 0.0482584518626148 |
| activation\_of\_MAPK\_activity | 0.00441466863713804 | 0.00327546821840804 | 0.0475791329184956 |
| activation\_of\_caspase\_activity | 0.00441466863713804 | 0.00327546821840804 | 0.0475791329184956 |
| ruffle | 0.0369747302118471 | 0.224672011837908 | 0.00666536274069951 |
| response\_to\_heat | 0.00419182561433567 | 0.00310956631694629 | 0.0468216270297582 |
| response\_to\_calcium\_ion | 0.0369747302118471 | 0.0297856531167195 | 0.0468216270297582 |
| mitochondrial\_membrane | 0.00419182561433567 | 0.0297856531167195 | 0.00666536274069951 |
| MyD88\_dependent\_toll\_like\_receptor\_signaling\_pathway | 5.65579039201229e-05 | 3.26033788573406e-05 | 0.0460588946342217 |
| integrin\_binding | 0.0357471257433936 | 0.0289541870158003 | 0.0460588946342217 |
| female\_pregnancy | 0.00403655750827302 | 0.0289541870158003 | 0.00666536274069951 |
| caveola | 0.00403655750827302 | 0.00294255688664503 | 0.0460588946342217 |
| transforming\_growth\_factor\_beta\_receptor\_signaling\_pathway | 0.0038237531456918 | 0.0279611305469009 | 0.00666536274069951 |
| response\_to\_mechanical\_stimulus | 5.20929623792665e-05 | 3.03920104810195e-05 | 0.0453603518869884 |
| regulation\_of\_growth | 0.0344838217251713 | 0.0279611305469009 | 0.0453603518869884 |
| regulation\_of\_cell\_shape | 0.0344838217251713 | 0.0279611305469009 | 0.0453603518869884 |
| PML\_body | 0.0344838217251713 | 0.0279611305469009 | 0.0453603518869884 |
| fatty\_acid\_biosynthetic\_process | 0.0038237531456918 | 0.00286905801999993 | 0.0453603518869884 |
| skeletal\_muscle\_tissue\_development | 0.0333308810779222 | 0.0270982839790266 | 0.0447927806020869 |
| response\_to\_retinoic\_acid | 0.0333308810779222 | 0.0270982839790266 | 0.0447927806020869 |
| positive\_regulation\_of\_cell\_growth | 0.0333308810779222 | 0.0270982839790266 | 0.0447927806020869 |
| phosphoric\_diester\_hydrolase\_activity | 0.0333308810779222 | 0.0270982839790266 | 0.0447927806020869 |
| myosin\_complex | 0.0333308810779222 | 0.0270982839790266 | 0.0447927806020869 |
| hemopoiesis | 0.0333308810779222 | 0.0270982839790266 | 0.0447927806020869 |
| Z\_disc | 0.0323722252889096 | 0.0262791000240143 | 0.0442847341642326 |
| positive\_regulation\_of\_peptidyl\_tyrosine\_phosphorylation | 0.00351329559759106 | 0.00254758218523444 | 0.0442847341642326 |
| glucose\_metabolic\_process | 0.0323722252889096 | 0.0262791000240143 | 0.0442847341642326 |
| blood\_vessel\_development | 0.00351329559759106 | 0.00254758218523444 | 0.0442847341642326 |
| response\_to\_wounding | 0.0312800198091346 | 0.0253118041154775 | 0.0436976506847505 |
| Ras\_protein\_signal\_transduction | 0.0312800198091346 | 0.0253118041154775 | 0.0436976506847505 |
| negative\_regulation\_of\_cell\_migration | 0.0312800198091346 | 0.0253118041154775 | 0.0436976506847505 |
| NAD\_binding | 0.0312800198091346 | 0.207190249470829 | 0.00666536274069951 |
| chemokine\_activity | 1.9355791511522e-06 | 1.00442627076513e-06 | 0.0436976506847505 |
| response\_to\_ionizing\_radiation | 0.0302401374247521 | 0.0243931529554277 | 0.0430324406868819 |
| positive\_regulation\_of\_sequence\_specific\_DNA\_binding\_transcription\_factor\_activity | 0.00313470874158206 | 0.00235406302807975 | 0.0430324406868819 |
| insoluble\_fraction | 0.00313470874158206 | 0.00235406302807975 | 0.0430324406868819 |
| early\_endosome\_membrane | 0.0302401374247521 | 0.0243931529554277 | 0.0430324406868819 |
| cysteine\_type\_endopeptidase\_activity | 0.00313470874158206 | 0.00235406302807975 | 0.0430324406868819 |
| cholesterol\_metabolic\_process | 0.00313470874158206 | 0.0243931529554277 | 0.00666536274069951 |
| protein\_tyrosine\_serine\_threonine\_phosphatase\_activity | 3.34965296093605e-05 | 1.96374840224565e-05 | 0.0424256848338654 |
| positive\_regulation\_of\_MAPKKK\_cascade | 0.00307496236909759 | 0.0022470252380005 | 0.0424256848338654 |
| positive\_regulation\_of\_angiogenesis | 0.00307496236909759 | 0.0022470252380005 | 0.0424256848338654 |
| palate\_development | 0.02907987671942 | 0.0236619705101444 | 0.0424256848338654 |
| cytoskeletal\_protein\_binding | 0.02907987671942 | 0.0236619705101444 | 0.0424256848338654 |
| response\_to\_hydrogen\_peroxide | 0.00288864471324536 | 0.00214197251462212 | 0.0418085389409436 |
| MyD88\_independent\_toll\_like\_receptor\_signaling\_pathway | 3.01340330303543e-05 | 1.82048873981812e-05 | 0.0418085389409436 |
| osteoblast\_differentiation | 0.0267275323554831 | 0.0217980124473289 | 0.0410476942824955 |
| negative\_regulation\_of\_gene\_expression | 0.00278907631974626 | 0.00200391007781613 | 0.0410476942824955 |
| vasculogenesis | 0.025617200452952 | 0.0208545000268568 | 0.0402135150161103 |
| regulation\_of\_translation | 0.025617200452952 | 0.0208545000268568 | 0.0402135150161103 |
| positive\_regulation\_of\_ERK1\_and\_ERK2\_cascade | 0.00261451245529628 | 0.00192129361855975 | 0.0402135150161103 |
| peptidyl\_serine\_phosphorylation | 0.025617200452952 | 0.0208545000268568 | 0.0402135150161103 |
| ovarian\_follicle\_development | 0.00261451245529628 | 0.00192129361855975 | 0.0402135150161103 |
| negative\_regulation\_of\_sequence\_specific\_DNA\_binding\_transcription\_factor\_activity | 0.025617200452952 | 0.0208545000268568 | 0.0402135150161103 |
| cellular\_response\_to\_mechanical\_stimulus | 0.025617200452952 | 0.0208545000268568 | 0.0402135150161103 |
| protease\_binding | 0.00028245262525049 | 0.000178858724948798 | 0.039695998342075 |
| ureteric\_bud\_development | 0.0223864649421446 | 0.018264163996962 | 0.0377980478079793 |
| response\_to\_organic\_nitrogen | 0.00023504902476083 | 0.000148027418332172 | 0.0377980478079793 |
| cellular\_response\_to\_lipopolysaccharide | 0.00023504902476083 | 0.000148027418332172 | 0.0377980478079793 |
| regulation\_of\_blood\_pressure | 0.00203429689156678 | 0.00151988946365714 | 0.036997759946152 |
| positive\_regulation\_of\_endothelial\_cell\_proliferation | 0.021312966334371 | 0.01738208933551 | 0.036997759946152 |
| negative\_regulation\_of\_protein\_kinase\_activity | 0.021312966334371 | 0.01738208933551 | 0.036997759946152 |
| negative\_regulation\_of\_caspase\_activity | 0.00203429689156678 | 0.00151988946365714 | 0.036997759946152 |
| fat\_cell\_differentiation | 0.021312966334371 | 0.01738208933551 | 0.036997759946152 |
| cartilage\_development | 0.021312966334371 | 0.01738208933551 | 0.036997759946152 |
| skin\_development | 0.0203505989532275 | 0.0166205884262033 | 0.0363697952976865 |
| positive\_regulation\_of\_T\_cell\_proliferation | 0.0203505989532275 | 0.0166205884262033 | 0.0363697952976865 |
| G1\_S\_transition\_of\_mitotic\_cell\_cycle | 0.0203505989532275 | 0.0166205884262033 | 0.0363697952976865 |
| cholesterol\_homeostasis | 0.0203505989532275 | 0.168110100771671 | 0.00666536274069951 |
| skeletal\_system\_morphogenesis | 0.0194595538862821 | 0.0158186112914054 | 0.035548936052361 |
| positive\_regulation\_of\_smooth\_muscle\_cell\_proliferation | 1.19085109286671e-05 | 6.6530678213326e-06 | 0.035548936052361 |
| positive\_regulation\_of\_DNA\_replication | 0.0194595538862821 | 0.0158186112914054 | 0.035548936052361 |
| memory | 0.00182876401433674 | 0.00132616428917994 | 0.035548936052361 |
| defense\_response\_to\_Gram\_positive\_bacterium | 0.00182876401433674 | 0.00132616428917994 | 0.035548936052361 |
| response\_to\_amino\_acid\_stimulus | 0.0184940877329524 | 0.0150068163613171 | 0.0348364108372264 |
| positive\_regulation\_of\_osteoblast\_differentiation | 0.0184940877329524 | 0.0150068163613171 | 0.0348364108372264 |
| negative\_regulation\_of\_signal\_transduction | 0.0184940877329524 | 0.0150068163613171 | 0.0348364108372264 |
| negative\_regulation\_of\_angiogenesis | 0.00169098269619842 | 0.00124925564394447 | 0.0348364108372264 |
| hydrolase\_activity\_acting\_on\_acid\_anhydrides | 0.0184940877329524 | 0.0150068163613171 | 0.0348364108372264 |
| positive\_regulation\_of\_protein\_ubiquitination | 0.00161995487607381 | 0.00114907652074298 | 0.034111070033959 |
| positive\_regulation\_of\_neuron\_apoptosis | 0.0175720771185005 | 0.0142562910109364 | 0.034111070033959 |
| positive\_regulation\_of\_anti\_apoptosis | 8.99757525048186e-06 | 0.000112657819014963 | 0.00587466856886691 |
| peptidyl\_tyrosine\_phosphorylation | 0.0175720771185005 | 0.0142562910109364 | 0.034111070033959 |
| manganese\_ion\_binding | 0.0175720771185005 | 0.0142562910109364 | 0.034111070033959 |
| keratinocyte\_differentiation | 0.0175720771185005 | 0.0142562910109364 | 0.034111070033959 |
| growth | 0.0175720771185005 | 0.0142562910109364 | 0.034111070033959 |
| cellular\_process | 0.0175720771185005 | 0.0142562910109364 | 0.034111070033959 |
| peptidyl\_tyrosine\_dephosphorylation | 0.016715852355423 | 0.0135828843533079 | 0.0334876607934358 |
| negative\_regulation\_of\_NF\_kappaB\_transcription\_factor\_activity | 0.00014187411301032 | 0.000110848165867642 | 0.0334876607934358 |
| response\_to\_UV | 0.0157494901647731 | 0.0128340193188597 | 0.0325641702773681 |
| iron\_sulfur\_cluster\_binding | 0.0157494901647731 | 0.0128340193188597 | 0.0325641702773681 |
| germ\_cell\_development | 0.0157494901647731 | 0.0128340193188597 | 0.0325641702773681 |
| coated\_pit | 0.0157494901647731 | 0.0128340193188597 | 0.0325641702773681 |
| cell\_growth | 0.0157494901647731 | 0.0128340193188597 | 0.0325641702773681 |
| calcium\_dependent\_protein\_binding | 0.0157494901647731 | 0.0128340193188597 | 0.0325641702773681 |
| response\_to\_testosterone\_stimulus | 0.0149212153669188 | 0.0121593971339812 | 0.031854789063065 |
| protein\_K48\_linked\_ubiquitination | 0.0149212153669188 | 0.0121593971339812 | 0.031854789063065 |
| positive\_regulation\_of\_JNK\_cascade | 0.00131132572837541 | 0.000937282809107365 | 0.031854789063065 |
| positive\_regulation\_of\_fibroblast\_proliferation | 0.000135285298034101 | 8.81527741315143e-05 | 0.031854789063065 |
| myelination | 0.0149212153669188 | 0.0121593971339812 | 0.031854789063065 |
| filopodium | 0.0149212153669188 | 0.0121593971339812 | 0.031854789063065 |
| defense\_response\_to\_virus | 0.000135285298034101 | 8.81527741315143e-05 | 0.031854789063065 |
| cellular\_response\_to\_hypoxia | 0.0149212153669188 | 0.0121593971339812 | 0.031854789063065 |
| activation\_of\_protein\_kinase\_C\_activity\_by\_G\_protein\_coupled\_receptor\_protein\_signaling\_pathway | 0.0149212153669188 | 0.0121593971339812 | 0.031854789063065 |
| ubiquitin\_specific\_protease\_activity | 0.0141052284873313 | 0.0115136625685882 | 0.0312955413589317 |
| response\_to\_interleukin\_1 | 0.0141052284873313 | 0.0115136625685882 | 0.0312955413589317 |
| response\_to\_chemical\_stimulus | 0.0141052284873313 | 0.0115136625685882 | 0.0312955413589317 |
| response\_to\_activity | 0.0141052284873313 | 0.0115136625685882 | 0.0312955413589317 |
| positive\_regulation\_of\_peptidyl\_serine\_phosphorylation | 0.00118810006557014 | 0.000851417669344871 | 0.0312955413589317 |
| positive\_regulation\_of\_cell\_division | 0.00118810006557014 | 0.000851417669344871 | 0.0312955413589317 |
| NADP\_binding | 0.0141052284873313 | 0.0115136625685882 | 0.0312955413589317 |
| lung\_alveolus\_development | 0.00118810006557014 | 0.000851417669344871 | 0.0312955413589317 |
| cellular\_response\_to\_hormone\_stimulus | 0.00118810006557014 | 0.000851417669344871 | 0.0312955413589317 |
| recycling\_endosome | 0.00108449010465551 | 0.0108409100133046 | 0.00433480283676363 |
| negative\_regulation\_of\_cell\_cycle | 0.0133231159952021 | 0.134652784732647 | 0.00433480283676363 |
| intrinsic\_to\_membrane | 0.0133231159952021 | 0.134652784732647 | 0.00433480283676363 |
| collagen\_binding | 0.0133231159952021 | 0.0108409100133046 | 0.0307174034238511 |
| response\_to\_cold | 0.012473295876244 | 0.0101642550747346 | 0.0296837147063588 |
| protein\_self\_association | 0.00101520763297025 | 0.000722427597525447 | 0.0296837147063588 |
| positive\_regulation\_of\_interleukin\_6\_production | 9.06876855842707e-05 | 5.91980786198842e-05 | 0.0296837147063588 |
| positive\_regulation\_of\_cell\_differentiation | 0.00101520763297025 | 0.000722427597525447 | 0.0296837147063588 |
| neuron\_development | 0.012473295876244 | 0.0101642550747346 | 0.0296837147063588 |
| embryonic\_hindlimb\_morphogenesis | 0.012473295876244 | 0.0101642550747346 | 0.0296837147063588 |
| response\_to\_progesterone\_stimulus | 0.000919527055872708 | 0.000652448527476547 | 0.0289073999007002 |
| response\_to\_amphetamine | 0.0117004068522328 | 0.00950311422318605 | 0.0289073999007002 |
| regulation\_of\_protein\_phosphorylation | 0.000919527055872708 | 0.000652448527476547 | 0.0289073999007002 |
| regulation\_of\_cyclin\_dependent\_protein\_kinase\_activity | 0.0117004068522328 | 0.00950311422318605 | 0.0289073999007002 |
| positive\_regulation\_of\_B\_cell\_proliferation | 0.000919527055872708 | 0.000652448527476547 | 0.0289073999007002 |
| learning\_or\_memory | 0.000919527055872708 | 0.000652448527476547 | 0.0289073999007002 |
| fertilization | 0.0117004068522328 | 0.00950311422318605 | 0.0289073999007002 |
| tumor\_necrosis\_factor\_receptor\_binding | 0.000829047623228615 | 0.000597494840585014 | 0.0281651884982738 |
| T\_cell\_receptor\_signaling\_pathway | 0.0109075273077917 | 0.00886632921087397 | 0.0281651884982738 |
| T\_cell\_activation | 0.0109075273077917 | 0.00886632921087397 | 0.0281651884982738 |
| ruffle\_membrane | 0.0109075273077917 | 0.00886632921087397 | 0.0281651884982738 |
| protein\_kinase\_C\_binding | 0.0109075273077917 | 0.00886632921087397 | 0.0281651884982738 |
| positive\_regulation\_of\_vasoconstriction | 6.82215935444005e-05 | 4.48223428413853e-05 | 0.0281651884982738 |
| positive\_regulation\_of\_protein\_kinase\_activity | 0.0109075273077917 | 0.00886632921087397 | 0.0281651884982738 |
| endocytic\_vesicle | 0.0109075273077917 | 0.00886632921087397 | 0.0281651884982738 |
| embryo\_implantation | 0.000829047623228615 | 0.000597494840585014 | 0.0281651884982738 |
| brown\_fat\_cell\_differentiation | 0.0109075273077917 | 0.00886632921087397 | 0.0281651884982738 |
| response\_to\_corticosterone\_stimulus | 0.000746240147514607 | 0.000584949015112981 | 0.0275578417277051 |
| response\_to\_bacterium | 0.0102341151082666 | 0.0083141741785127 | 0.0275578417277051 |
| protein\_oligomerization | 0.0102341151082666 | 0.0083141741785127 | 0.0275578417277051 |
| positive\_regulation\_of\_phosphorylation | 0.000746240147514607 | 0.000584949015112981 | 0.0275578417277051 |
| positive\_regulation\_of\_cell\_adhesion | 0.0102341151082666 | 0.0083141741785127 | 0.0275578417277051 |
| negative\_regulation\_of\_osteoblast\_differentiation | 0.0102341151082666 | 0.0083141741785127 | 0.0275578417277051 |
| amino\_acid\_binding | 0.0102341151082666 | 0.0083141741785127 | 0.0275578417277051 |
| 3\_5\_\_cyclic\_nucleotide\_phosphodiesterase\_activity | 0.0102341151082666 | 0.0083141741785127 | 0.0275578417277051 |
| ubiquitin\_binding | 0.00952267158311468 | 0.00773727191637304 | 0.0268232001500029 |
| response\_to\_radiation | 0.00952267158311468 | 0.00773727191637304 | 0.0268232001500029 |
| protein\_disulfide\_oxidoreductase\_activity | 0.00952267158311468 | 0.00773727191637304 | 0.0268232001500029 |
| positive\_regulation\_of\_protein\_catabolic\_process | 0.00952267158311468 | 0.00773727191637304 | 0.0268232001500029 |
| positive\_regulation\_of\_MAP\_kinase\_activity | 0.000730176017012143 | 0.000519176163913021 | 0.0268232001500029 |
| blood\_vessel\_remodeling | 0.00952267158311468 | 0.00773727191637304 | 0.0268232001500029 |
| response\_to\_morphine | 0.00882685347351023 | 0.00714792267412924 | 0.0259638849409864 |
| response\_to\_gamma\_radiation | 4.37817919639516e-05 | 2.92181663232368e-05 | 0.0259638849409864 |
| positive\_regulation\_of\_nitric\_oxide\_biosynthetic\_process | 1.28409183732216e-06 | 7.03418609336754e-07 | 0.0259638849409864 |
| positive\_regulation\_of\_neuron\_projection\_development | 0.00882685347351023 | 0.00714792267412924 | 0.0259638849409864 |
| positive\_regulation\_of\_interferon\_gamma\_production | 0.000644099265172772 | 0.000454648647981594 | 0.0259638849409864 |
| platelet\_activation | 0.00882685347351023 | 0.00714792267412924 | 0.0259638849409864 |
| negative\_regulation\_of\_smooth\_muscle\_cell\_proliferation | 0.00882685347351023 | 0.00714792267412924 | 0.0259638849409864 |
| metallocarboxypeptidase\_activity | 0.00882685347351023 | 0.00714792267412924 | 0.0259638849409864 |
| cortical\_cytoskeleton | 0.000644099265172772 | 0.000454648647981594 | 0.0259638849409864 |
| cell\_aging | 0.00882685347351023 | 0.00714792267412924 | 0.0259638849409864 |
| vesicle | 0.0081475836449886 | 0.00668144206998472 | 0.0252818543911357 |
| spleen\_development | 0.0081475836449886 | 0.00668144206998472 | 0.0252818543911357 |
| regulation\_of\_insulin\_secretion | 3.77862786049452e-05 | 2.42556100131846e-05 | 0.0252818543911357 |
| regulation\_of\_cell\_adhesion | 0.0081475836449886 | 0.00668144206998472 | 0.0252818543911357 |
| positive\_regulation\_of\_mitotic\_cell\_cycle | 0.0081475836449886 | 0.00668144206998472 | 0.0252818543911357 |
| positive\_regulation\_of\_mitosis | 3.77862786049452e-05 | 2.42556100131846e-05 | 0.0252818543911357 |
| platelet\_derived\_growth\_factor\_receptor\_signaling\_pathway | 0.000566359659258799 | 0.000415532941492481 | 0.0252818543911357 |
| eukaryotic\_cell\_surface\_binding | 0.0081475836449886 | 0.00668144206998472 | 0.0252818543911357 |
| endoplasmic\_reticulum\_unfolded\_protein\_response | 0.0081475836449886 | 0.00668144206998472 | 0.0252818543911357 |
| embryonic\_placenta\_development | 0.0081475836449886 | 0.00668144206998472 | 0.0252818543911357 |
| calcium\_mediated\_signaling | 3.77862786049452e-05 | 2.42556100131846e-05 | 0.0252818543911357 |
| activation\_of\_MAPKK\_activity | 0.0081475836449886 | 0.00668144206998472 | 0.0252818543911357 |
| social\_behavior | 0.00749865033322356 | 0.00616294168981821 | 0.0247210371353784 |
| response\_to\_hyperoxia | 0.00749865033322356 | 0.00616294168981821 | 0.0247210371353784 |
| regulation\_of\_cell\_differentiation | 0.000520415395319939 | 0.000405442924715895 | 0.0247210371353784 |
| recycling\_endosome\_membrane | 0.00749865033322356 | 0.00616294168981821 | 0.0247210371353784 |
| protein\_ubiquitination\_involved\_in\_ubiquitin\_dependent\_protein\_catabolic\_process | 0.00749865033322356 | 0.0994535178509179 | 0.00239099804877283 |
| positive\_regulation\_of\_JUN\_kinase\_activity | 0.00749865033322356 | 0.00616294168981821 | 0.0247210371353784 |
| positive\_regulation\_of\_inflammatory\_response | 0.000520415395319939 | 0.000405442924715895 | 0.0247210371353784 |
| JNK\_cascade | 0.00749865033322356 | 0.00616294168981821 | 0.0247210371353784 |
| cholesterol\_efflux | 0.00749865033322356 | 0.0994535178509179 | 0.00239099804877283 |
| release\_of\_cytochrome\_c\_from\_mitochondria | 0.00686637808643375 | 0.00563541352751876 | 0.0238311179819602 |
| peroxidase\_activity | 0.00686637808643375 | 0.00563541352751876 | 0.0238311179819602 |
| neutrophil\_chemotaxis | 6.01655972935787e-07 | 3.24551670644384e-07 | 0.0238311179819602 |
| leukocyte\_cell\_cell\_adhesion | 0.000494822812770528 | 0.000348847824811438 | 0.0238311179819602 |
| epidermal\_growth\_factor\_receptor\_signaling\_pathway | 0.00686637808643375 | 0.00563541352751876 | 0.0238311179819602 |
| B\_cell\_activation | 0.000494822812770528 | 0.000348847824811438 | 0.0238311179819602 |
| stem\_cell\_maintenance | 0.00620814503456133 | 0.00508574134899153 | 0.0229622890553296 |
| response\_to\_vitamin\_D | 2.25168429430437e-05 | 1.42597176180038e-05 | 0.0229622890553296 |
| response\_to\_copper\_ion | 0.00620814503456133 | 0.00508574134899153 | 0.0229622890553296 |
| regulation\_of\_neuronal\_synaptic\_plasticity | 0.00620814503456133 | 0.00508574134899153 | 0.0229622890553296 |
| regulation\_of\_mitotic\_cell\_cycle | 0.00620814503456133 | 0.00508574134899153 | 0.0229622890553296 |
| phospholipid\_transport | 0.00620814503456133 | 0.0908480779972051 | 0.00205543292895224 |
| microtubule\_associated\_complex | 0.00620814503456133 | 0.00508574134899153 | 0.0229622890553296 |
| cellular\_response\_to\_tumor\_necrosis\_factor | 0.00620814503456133 | 0.00508574134899153 | 0.0229622890553296 |
| response\_to\_light\_stimulus | 0.000352940423873158 | 0.000251200728326204 | 0.0220161858753813 |
| protein\_import\_into\_nucleus\_translocation | 0.000352940423873158 | 0.000251200728326204 | 0.0220161858753813 |
| positive\_regulation\_of\_cytokine\_secretion | 0.00560621702811922 | 0.00458549698732288 | 0.0220161858753813 |
| positive\_regulation\_of\_caspase\_activity | 0.00560621702811922 | 0.00458549698732288 | 0.0220161858753813 |
| maternal\_process\_involved\_in\_female\_pregnancy | 0.00560621702811922 | 0.00458549698732288 | 0.0220161858753813 |
| lipopolysaccharide\_mediated\_signaling\_pathway | 0.00560621702811922 | 0.00458549698732288 | 0.0220161858753813 |
| I\_kappaB\_kinase\_NF\_kappaB\_cascade | 0.00560621702811922 | 0.00458549698732288 | 0.0220161858753813 |
| gluconeogenesis | 0.00560621702811922 | 0.00458549698732288 | 0.0220161858753813 |
| G2\_M\_transition\_of\_mitotic\_cell\_cycle | 0.00560621702811922 | 0.00458549698732288 | 0.0220161858753813 |
| decidualization | 1.77924672028374e-05 | 1.14163047226767e-05 | 0.0220161858753813 |
| cellular\_response\_to\_interferon\_gamma | 0.00560621702811922 | 0.00458549698732288 | 0.0220161858753813 |
| cellular\_response\_to\_extracellular\_stimulus | 0.00560621702811922 | 0.00458549698732288 | 0.0220161858753813 |
| ADP\_binding | 0.00560621702811922 | 0.0864444606691439 | 0.00189083144679032 |
| activation\_of\_protein\_kinase\_activity | 0.00560621702811922 | 0.00458549698732288 | 0.0220161858753813 |
| T\_cell\_proliferation | 0.005062494299483 | 0.00413597449762046 | 0.0213163127239686 |
| positive\_regulation\_of\_activated\_T\_cell\_proliferation | 0.005062494299483 | 0.00413597449762046 | 0.0213163127239686 |
| lymph\_node\_development | 0.000314914210087869 | 0.000251200728326204 | 0.0213163127239686 |
| leukocyte\_migration | 2.36427813664941e-07 | 1.32089124777198e-07 | 0.0213163127239686 |
| heterophilic\_cell\_cell\_adhesion | 0.005062494299483 | 0.00413597449762046 | 0.0213163127239686 |
| endosome\_transport | 0.005062494299483 | 0.00413597449762046 | 0.0213163127239686 |
| cytokine\_production | 0.005062494299483 | 0.00413597449762046 | 0.0213163127239686 |
| cellular\_response\_to\_heat | 0.005062494299483 | 0.00413597449762046 | 0.0213163127239686 |
| cellular\_response\_to\_cAMP | 0.005062494299483 | 0.00413597449762046 | 0.0213163127239686 |
| rhythmic\_process | 0.00451028482898884 | 0.00368641664958009 | 0.0203913518060153 |
| response\_to\_ATP | 0.00451028482898884 | 0.00368641664958009 | 0.0203913518060153 |
| positive\_regulation\_of\_T\_cell\_activation | 0.00451028482898884 | 0.00368641664958009 | 0.0203913518060153 |
| phospholipase\_binding | 0.00451028482898884 | 0.00368641664958009 | 0.0203913518060153 |
| NF\_kappaB\_binding | 0.00451028482898884 | 0.00368641664958009 | 0.0203913518060153 |
| negative\_regulation\_of\_protein\_ubiquitination | 0.00451028482898884 | 0.00368641664958009 | 0.0203913518060153 |
| negative\_regulation\_of\_protein\_catabolic\_process | 0.00451028482898884 | 0.00368641664958009 | 0.0203913518060153 |
| negative\_regulation\_of\_insulin\_receptor\_signaling\_pathway | 0.00451028482898884 | 0.00368641664958009 | 0.0203913518060153 |
| negative\_regulation\_of\_growth\_of\_symbiont\_in\_host | 0.00451028482898884 | 0.00368641664958009 | 0.0203913518060153 |
| fibronectin\_binding | 0.000297509975706706 | 0.000212956109747467 | 0.0203913518060153 |
| face\_morphogenesis | 0.00451028482898884 | 0.00368641664958009 | 0.0203913518060153 |
| epidermal\_growth\_factor\_receptor\_binding | 0.00451028482898884 | 0.00368641664958009 | 0.0203913518060153 |
| diacylglycerol\_kinase\_activity | 0.00451028482898884 | 0.00368641664958009 | 0.0203913518060153 |
| cell\_chemotaxis | 0.00451028482898884 | 0.00368641664958009 | 0.0203913518060153 |
| water\_transport | 0.00404862828822035 | 0.00329420438214095 | 0.0196509436753201 |
| skeletal\_muscle\_fiber\_development | 0.000241507613113324 | 0.000172899804669081 | 0.0196509436753201 |
| protein\_export\_from\_nucleus | 0.00404862828822035 | 0.00329420438214095 | 0.0196509436753201 |
| positive\_regulation\_of\_phosphatidylinositol\_3\_kinase\_cascade | 0.00404862828822035 | 0.00329420438214095 | 0.0196509436753201 |
| positive\_regulation\_of\_osteoclast\_differentiation | 0.00404862828822035 | 0.00329420438214095 | 0.0196509436753201 |
| negative\_regulation\_of\_ERK1\_and\_ERK2\_cascade | 0.00404862828822035 | 0.00329420438214095 | 0.0196509436753201 |
| negative\_regulation\_of\_cyclin\_dependent\_protein\_kinase\_activity | 0.00404862828822035 | 0.00329420438214095 | 0.0196509436753201 |
| motor\_axon\_guidance | 0.00404862828822035 | 0.00329420438214095 | 0.0196509436753201 |
| cellular\_response\_to\_glucose\_stimulus | 0.00404862828822035 | 0.00329420438214095 | 0.0196509436753201 |
| bone\_mineralization | 0.000241507613113324 | 0.000172899804669081 | 0.0196509436753201 |
| tumor\_necrosis\_factor\_mediated\_signaling\_pathway | 0.00356321265621732 | 0.00290173410415799 | 0.0186828272765057 |
| trophectodermal\_cell\_differentiation | 0.00356321265621732 | 0.00290173410415799 | 0.0186828272765057 |
| superoxide\_metabolic\_process | 0.00356321265621732 | 0.00290173410415799 | 0.0186828272765057 |
| retinol\_dehydrogenase\_activity | 0.00356321265621732 | 0.00290173410415799 | 0.0186828272765057 |
| response\_to\_food | 0.00356321265621732 | 0.00290173410415799 | 0.0186828272765057 |
| response\_to\_arsenic\_containing\_substance | 0.00356321265621732 | 0.00290173410415799 | 0.0186828272765057 |
| reactive\_oxygen\_species\_metabolic\_process | 0.00356321265621732 | 0.00290173410415799 | 0.0186828272765057 |
| prostaglandin\_biosynthetic\_process | 0.00356321265621732 | 0.00290173410415799 | 0.0186828272765057 |
| positive\_regulation\_of\_tyrosine\_phosphorylation\_of\_Stat3\_protein | 0.00356321265621732 | 0.00290173410415799 | 0.0186828272765057 |
| positive\_regulation\_of\_synaptic\_transmission | 0.00356321265621732 | 0.00290173410415799 | 0.0186828272765057 |
| positive\_regulation\_of\_stress\_fiber\_assembly | 0.00356321265621732 | 0.00290173410415799 | 0.0186828272765057 |
| positive\_regulation\_of\_smooth\_muscle\_contraction | 0.000197947571885205 | 0.000139502044665534 | 0.0186828272765057 |
| osteoclast\_differentiation | 0.00356321265621732 | 0.00290173410415799 | 0.0186828272765057 |
| negative\_regulation\_of\_hormone\_secretion | 0.00356321265621732 | 0.00290173410415799 | 0.0186828272765057 |
| immunological\_synapse | 0.00356321265621732 | 0.00290173410415799 | 0.0186828272765057 |
| embryonic\_digestive\_tract\_development | 0.00356321265621732 | 0.00290173410415799 | 0.0186828272765057 |
| cholesterol\_transport | 0.00356321265621732 | 0.0679496365538572 | 0.0011250286952875 |
| cell\_surface\_binding | 0.000197947571885205 | 0.000139502044665534 | 0.0186828272765057 |
| ribonucleoprotein\_binding | 0.00313470874158206 | 0.00254758218523444 | 0.0180587191954625 |
| retinol\_metabolic\_process | 0.00313470874158206 | 0.00254758218523444 | 0.0180587191954625 |
| response\_to\_molecule\_of\_bacterial\_origin | 4.22514455726212e-06 | 2.61901011047866e-06 | 0.0180587191954625 |
| receptor\_internalization | 0.00313470874158206 | 0.00254758218523444 | 0.0180587191954625 |
| protein\_phosphatase\_2A\_binding | 0.00313470874158206 | 0.00254758218523444 | 0.0180587191954625 |
| positive\_regulation\_of\_smooth\_muscle\_cell\_migration | 0.00313470874158206 | 0.00254758218523444 | 0.0180587191954625 |
| positive\_regulation\_of\_phosphatidylinositol\_3\_kinase\_activity | 0.00313470874158206 | 0.00254758218523444 | 0.0180587191954625 |
| positive\_regulation\_of\_NF\_kappaB\_import\_into\_nucleus | 4.22514455726212e-06 | 2.61901011047866e-06 | 0.0180587191954625 |
| positive\_regulation\_of\_neutrophil\_chemotaxis | 0.00313470874158206 | 0.00254758218523444 | 0.0180587191954625 |
| positive\_regulation\_of\_interleukin\_8\_production | 0.00313470874158206 | 0.00254758218523444 | 0.0180587191954625 |
| positive\_regulation\_of\_interleukin\_10\_production | 0.00313470874158206 | 0.00254758218523444 | 0.0180587191954625 |
| positive\_regulation\_of\_cytokine\_production | 0.00015546848590202 | 0.000112657819014963 | 0.0180587191954625 |
| positive\_regulation\_of\_chemokine\_production | 0.00313470874158206 | 0.00254758218523444 | 0.0180587191954625 |
| positive\_regulation\_of\_cell\_cell\_adhesion | 0.00313470874158206 | 0.00254758218523444 | 0.0180587191954625 |
| osteoblast\_development | 0.00313470874158206 | 0.00254758218523444 | 0.0180587191954625 |
| negative\_regulation\_of\_interleukin\_6\_production | 0.00313470874158206 | 0.00254758218523444 | 0.0180587191954625 |
| MAP\_kinase\_tyrosine\_serine\_threonine\_phosphatase\_activity | 4.22514455726212e-06 | 2.61901011047866e-06 | 0.0180587191954625 |
| labyrinthine\_layer\_blood\_vessel\_development | 0.00313470874158206 | 0.00254758218523444 | 0.0180587191954625 |
| isotype\_switching | 0.00313470874158206 | 0.00254758218523444 | 0.0180587191954625 |
| induction\_of\_positive\_chemotaxis | 0.00313470874158206 | 0.0632856755908567 | 0.000987611159834086 |
| FMN\_binding | 0.00313470874158206 | 0.00254758218523444 | 0.0180587191954625 |
| DNA\_damage\_response\_signal\_transduction\_resulting\_in\_induction\_of\_apoptosis | 0.00313470874158206 | 0.00254758218523444 | 0.0180587191954625 |
| costamere | 0.00015546848590202 | 0.000112657819014963 | 0.0180587191954625 |
| collagen\_catabolic\_process | 0.00313470874158206 | 0.00254758218523444 | 0.0180587191954625 |
| cholesterol\_transporter\_activity | 0.00313470874158206 | 0.0632856755908567 | 0.000987611159834086 |
| cellular\_response\_to\_interleukin\_1 | 0.00313470874158206 | 0.00254758218523444 | 0.0180587191954625 |
| cellular\_response\_to\_calcium\_ion | 0.00015546848590202 | 0.000112657819014963 | 0.0180587191954625 |
| skeletal\_muscle\_tissue\_regeneration | 0.00278907631974626 | 0.0022470252380005 | 0.0176458673290131 |
| response\_to\_lipid | 0.00278907631974626 | 0.0585352194281846 | 0.000889216044733887 |
| response\_to\_exogenous\_dsRNA | 0.00278907631974626 | 0.0022470252380005 | 0.0176458673290131 |
| regulation\_of\_ossification | 0.00014187411301032 | 0.000112657819014963 | 0.0176458673290131 |
| positive\_regulation\_of\_protein\_tyrosine\_kinase\_activity | 0.00278907631974626 | 0.0022470252380005 | 0.0176458673290131 |
| positive\_regulation\_of\_blood\_vessel\_endothelial\_cell\_migration | 0.00278907631974626 | 0.0022470252380005 | 0.0176458673290131 |
| platelet\_derived\_growth\_factor\_binding | 0.00278907631974626 | 0.0022470252380005 | 0.0176458673290131 |
| phosphatidylinositol\_3\_kinase\_complex | 0.00278907631974626 | 0.0022470252380005 | 0.0176458673290131 |
| phagocytic\_cup | 0.00278907631974626 | 0.0022470252380005 | 0.0176458673290131 |
| negative\_regulation\_of\_phosphorylation | 0.00278907631974626 | 0.0022470252380005 | 0.0176458673290131 |
| NAD\_\_binding | 0.00278907631974626 | 0.0585352194281846 | 0.000889216044733887 |
| estradiol\_17\_beta\_dehydrogenase\_activity | 0.00278907631974626 | 0.0022470252380005 | 0.0176458673290131 |
| carboxy\_lyase\_activity | 0.00278907631974626 | 0.0022470252380005 | 0.0176458673290131 |
| branching\_involved\_in\_salivary\_gland\_morphogenesis | 0.00278907631974626 | 0.0022470252380005 | 0.0176458673290131 |
| 3\_5\_\_cyclic\_AMP\_phosphodiesterase\_activity | 0.00278907631974626 | 0.0022470252380005 | 0.0176458673290131 |
| water\_channel\_activity | 0.00238664553160006 | 0.00192129361855975 | 0.0166138796750628 |
| skeletal\_muscle\_contraction | 0.000125349867503251 | 9.0284678117094e-05 | 0.0166138796750628 |
| protein\_kinase\_inhibitor\_activity | 0.00238664553160006 | 0.00192129361855975 | 0.0166138796750628 |
| protein\_K63\_linked\_deubiquitination | 0.00238664553160006 | 0.00192129361855975 | 0.0166138796750628 |
| positive\_regulation\_of\_reactive\_oxygen\_species\_metabolic\_process | 0.00238664553160006 | 0.00192129361855975 | 0.0166138796750628 |
| positive\_regulation\_of\_interleukin\_2\_biosynthetic\_process | 0.00238664553160006 | 0.00192129361855975 | 0.0166138796750628 |
| positive\_regulation\_of\_interleukin\_1\_beta\_secretion | 0.00238664553160006 | 0.00192129361855975 | 0.0166138796750628 |
| platelet\_derived\_growth\_factor\_receptor\_binding | 0.00238664553160006 | 0.00192129361855975 | 0.0166138796750628 |
| phospholipid\_efflux | 0.00238664553160006 | 0.0538417276714085 | 0.000791130417774214 |
| negative\_regulation\_of\_cytokine\_secretion | 0.00238664553160006 | 0.00192129361855975 | 0.0166138796750628 |
| JAK\_STAT\_cascade | 0.00238664553160006 | 0.00192129361855975 | 0.0166138796750628 |
| humoral\_immune\_response | 0.00238664553160006 | 0.00192129361855975 | 0.0166138796750628 |
| gene\_expression | 0.00238664553160006 | 0.00192129361855975 | 0.0166138796750628 |
| extrinsic\_to\_internal\_side\_of\_plasma\_membrane | 0.00238664553160006 | 0.00192129361855975 | 0.0166138796750628 |
| cellular\_senescence | 0.00238664553160006 | 0.00192129361855975 | 0.0166138796750628 |
| cellular\_response\_to\_amino\_acid\_stimulus | 0.00238664553160006 | 0.00192129361855975 | 0.0166138796750628 |
| carboxylic\_acid\_metabolic\_process | 0.00238664553160006 | 0.00192129361855975 | 0.0166138796750628 |
| cAMP\_catabolic\_process | 0.00238664553160006 | 0.00192129361855975 | 0.0166138796750628 |
| activation\_of\_phospholipase\_C\_activity | 0.00238664553160006 | 0.00192129361855975 | 0.0166138796750628 |
| sterol\_biosynthetic\_process | 0.00200999030204701 | 0.00161694483310824 | 0.0157717379608665 |
| reverse\_cholesterol\_transport | 0.00200999030204701 | 0.0491008874142984 | 0.000661945179427008 |
| response\_to\_mercury\_ion | 0.00200999030204701 | 0.00161694483310824 | 0.0157717379608665 |
| response\_to\_manganese\_ion | 0.00200999030204701 | 0.00161694483310824 | 0.0157717379608665 |
| regulation\_of\_peptidyl\_tyrosine\_phosphorylation | 0.00200999030204701 | 0.00161694483310824 | 0.0157717379608665 |
| regulation\_of\_osteoclast\_differentiation | 0.00200999030204701 | 0.00161694483310824 | 0.0157717379608665 |
| regulation\_of\_inhibitory\_postsynaptic\_membrane\_potential | 0.00200999030204701 | 0.00161694483310824 | 0.0157717379608665 |
| positive\_regulation\_of\_protein\_autophosphorylation | 0.00200999030204701 | 0.00161694483310824 | 0.0157717379608665 |
| positive\_regulation\_of\_interleukin\_2\_production | 9.03469813433671e-05 | 6.57638148193068e-05 | 0.0157717379608665 |
| positive\_regulation\_of\_cellular\_protein\_metabolic\_process | 0.00200999030204701 | 0.00161694483310824 | 0.0157717379608665 |
| negative\_regulation\_of\_I\_kappaB\_kinase\_NF\_kappaB\_cascade | 0.00200999030204701 | 0.00161694483310824 | 0.0157717379608665 |
| high\_density\_lipoprotein\_particle\_remodeling | 0.00200999030204701 | 0.0491008874142984 | 0.000661945179427008 |
| endocytic\_recycling | 0.00200999030204701 | 0.00161694483310824 | 0.0157717379608665 |
| cyclin\_dependent\_protein\_kinase\_inhibitor\_activity | 0.00200999030204701 | 0.00161694483310824 | 0.0157717379608665 |
| cyclin\_binding | 0.00200999030204701 | 0.00161694483310824 | 0.0157717379608665 |
| cellular\_response\_to\_organic\_substance | 0.00200999030204701 | 0.00161694483310824 | 0.0157717379608665 |
| anagen | 0.00200999030204701 | 0.00161694483310824 | 0.0157717379608665 |
| activation\_of\_protein\_kinase\_B\_activity | 0.00200999030204701 | 0.00161694483310824 | 0.0157717379608665 |
| activation\_of\_NF\_kappaB\_inducing\_kinase\_activity | 0.00200999030204701 | 0.00161694483310824 | 0.0157717379608665 |
| wound\_healing\_spreading\_of\_epidermal\_cells | 0.00166985020981721 | 0.00132616428917994 | 0.0148313990507353 |
| regulation\_of\_mitochondrial\_membrane\_permeability | 0.00166985020981721 | 0.00132616428917994 | 0.0148313990507353 |
| protein\_secretion | 0.00166985020981721 | 0.00132616428917994 | 0.0148313990507353 |
| positive\_regulation\_vascular\_endothelial\_growth\_factor\_production | 6.26075250267235e-05 | 4.48223428413853e-05 | 0.0148313990507353 |
| positive\_regulation\_of\_stress\_activated\_MAPK\_cascade | 0.00166985020981721 | 0.00132616428917994 | 0.0148313990507353 |
| positive\_regulation\_of\_protein\_complex\_assembly | 0.00166985020981721 | 0.00132616428917994 | 0.0148313990507353 |
| positive\_regulation\_of\_membrane\_protein\_ectodomain\_proteolysis | 6.26075250267235e-05 | 4.48223428413853e-05 | 0.0148313990507353 |
| positive\_regulation\_of\_macrophage\_chemotaxis | 6.26075250267235e-05 | 4.48223428413853e-05 | 0.0148313990507353 |
| positive\_regulation\_of\_macrophage\_activation | 0.00166985020981721 | 0.00132616428917994 | 0.0148313990507353 |
| positive\_regulation\_of\_cyclin\_dependent\_protein\_kinase\_activity | 0.00166985020981721 | 0.00132616428917994 | 0.0148313990507353 |
| positive\_regulation\_of\_chemokine\_biosynthetic\_process | 6.26075250267235e-05 | 4.48223428413853e-05 | 0.0148313990507353 |
| ovulation | 6.26075250267235e-05 | 4.48223428413853e-05 | 0.0148313990507353 |
| negative\_regulation\_of\_protein\_secretion | 0.00166985020981721 | 0.00132616428917994 | 0.0148313990507353 |
| negative\_regulation\_of\_peptidyl\_tyrosine\_phosphorylation | 0.00166985020981721 | 0.00132616428917994 | 0.0148313990507353 |
| negative\_regulation\_of\_interferon\_gamma\_production | 0.00166985020981721 | 0.00132616428917994 | 0.0148313990507353 |
| muscle\_organ\_morphogenesis | 0.00166985020981721 | 0.00132616428917994 | 0.0148313990507353 |
| leukocyte\_tethering\_or\_rolling | 6.26075250267235e-05 | 4.48223428413853e-05 | 0.0148313990507353 |
| intercellular\_canaliculus | 0.00166985020981721 | 0.00132616428917994 | 0.0148313990507353 |
| induction\_of\_apoptosis\_via\_death\_domain\_receptors | 0.00166985020981721 | 0.00132616428917994 | 0.0148313990507353 |
| embryonic\_process\_involved\_in\_female\_pregnancy | 0.00166985020981721 | 0.00132616428917994 | 0.0148313990507353 |
| cyclin\_dependent\_protein\_kinase\_holoenzyme\_complex | 0.00166985020981721 | 0.00132616428917994 | 0.0148313990507353 |
| cell\_projection\_assembly | 0.00166985020981721 | 0.00132616428917994 | 0.0148313990507353 |
| response\_to\_peptidoglycan | 0.00131553477532275 | 0.00106019304269872 | 0.0139090353445293 |
| response\_to\_L\_ascorbic\_acid | 0.00131553477532275 | 0.00106019304269872 | 0.0139090353445293 |
| response\_to\_gonadotropin\_stimulus | 0.00131553477532275 | 0.00106019304269872 | 0.0139090353445293 |
| response\_to\_fructose\_stimulus | 0.00131553477532275 | 0.00106019304269872 | 0.0139090353445293 |
| regulation\_of\_protein\_secretion | 0.00131553477532275 | 0.00106019304269872 | 0.0139090353445293 |
| regulation\_of\_phosphoprotein\_phosphatase\_activity | 0.00131553477532275 | 0.00106019304269872 | 0.0139090353445293 |
| regulation\_of\_mitochondrial\_membrane\_potential | 0.00131553477532275 | 0.00106019304269872 | 0.0139090353445293 |
| prostaglandin\_metabolic\_process | 0.00131553477532275 | 0.0392247357326681 | 0.000425098047691902 |
| positive\_regulation\_of\_synaptic\_transmission\_glutamatergic | 0.00131553477532275 | 0.00106019304269872 | 0.0139090353445293 |
| positive\_regulation\_of\_receptor\_internalization | 0.00131553477532275 | 0.00106019304269872 | 0.0139090353445293 |
| positive\_regulation\_of\_leukocyte\_migration | 0.00131553477532275 | 0.00106019304269872 | 0.0139090353445293 |
| positive\_regulation\_of\_interleukin\_4\_production | 0.00131553477532275 | 0.00106019304269872 | 0.0139090353445293 |
| positive\_regulation\_of\_interleukin\_12\_biosynthetic\_process | 4.30967865118865e-05 | 3.10377631224965e-05 | 0.0139090353445293 |
| positive\_regulation\_of\_cholesterol\_efflux | 4.30967865118865e-05 | 0.00106019304269872 | 0.000425098047691902 |
| positive\_regulation\_of\_chemotaxis | 0.00131553477532275 | 0.00106019304269872 | 0.0139090353445293 |
| oligopeptide\_transport | 0.00131553477532275 | 0.00106019304269872 | 0.0139090353445293 |
| negative\_regulation\_of\_protein\_autophosphorylation | 0.00131553477532275 | 0.00106019304269872 | 0.0139090353445293 |
| negative\_regulation\_of\_Notch\_signaling\_pathway | 0.00131553477532275 | 0.00106019304269872 | 0.0139090353445293 |
| negative\_regulation\_of\_lipid\_catabolic\_process | 3.73855973583506e-07 | 2.34061670329883e-07 | 0.0139090353445293 |
| negative\_regulation\_of\_interleukin\_2\_production | 0.00131553477532275 | 0.00106019304269872 | 0.0139090353445293 |
| negative\_regulation\_of\_epidermal\_growth\_factor\_receptor\_signaling\_pathway | 0.00131553477532275 | 0.00106019304269872 | 0.0139090353445293 |
| mesenchymal\_cell\_differentiation | 0.00131553477532275 | 0.00106019304269872 | 0.0139090353445293 |
| lung\_vasculature\_development | 4.30967865118865e-05 | 3.10377631224965e-05 | 0.0139090353445293 |
| leukocyte\_chemotaxis | 4.30967865118865e-05 | 0.00106019304269872 | 0.000425098047691902 |
| interleukin\_1\_receptor\_binding | 0.00131553477532275 | 0.00106019304269872 | 0.0139090353445293 |
| estrogen\_biosynthetic\_process | 0.00131553477532275 | 0.00106019304269872 | 0.0139090353445293 |
| cellular\_response\_to\_ionizing\_radiation | 0.00131553477532275 | 0.00106019304269872 | 0.0139090353445293 |
| cellular\_response\_to\_gamma\_radiation | 0.00131553477532275 | 0.00106019304269872 | 0.0139090353445293 |
| cell\_activation | 0.00131553477532275 | 0.00106019304269872 | 0.0139090353445293 |
| attachment\_of\_GPI\_anchor\_to\_protein | 0.00131553477532275 | 0.00106019304269872 | 0.0139090353445293 |
| trophoblast\_giant\_cell\_differentiation | 2.7679213127304e-05 | 1.96374840224565e-05 | 0.013118571060022 |
| response\_to\_muramyl\_dipeptide | 0.00103136460248285 | 0.000832159268175552 | 0.013118571060022 |
| regulation\_of\_I\_kappaB\_kinase\_NF\_kappaB\_cascade | 2.7679213127304e-05 | 1.96374840224565e-05 | 0.013118571060022 |
| positive\_regulation\_of\_T\_cell\_cytokine\_production | 0.00103136460248285 | 0.000832159268175552 | 0.013118571060022 |
| positive\_regulation\_of\_immunoglobulin\_secretion | 0.00103136460248285 | 0.000832159268175552 | 0.013118571060022 |
| positive\_regulation\_of\_fever\_generation | 2.7679213127304e-05 | 1.96374840224565e-05 | 0.013118571060022 |
| positive\_regulation\_of\_B\_cell\_activation | 0.00103136460248285 | 0.000832159268175552 | 0.013118571060022 |
| phospholipid\_homeostasis | 0.00103136460248285 | 0.0343229969759492 | 0.000357372358029461 |
| negative\_regulation\_of\_viral\_genome\_replication | 0.00103136460248285 | 0.000832159268175552 | 0.013118571060022 |
| negative\_regulation\_of\_lipid\_storage | 2.7679213127304e-05 | 0.000832159268175552 | 0.000357372358029461 |
| negative\_regulation\_of\_glutamate\_secretion | 0.00103136460248285 | 0.000832159268175552 | 0.013118571060022 |
| negative\_regulation\_of\_glucose\_import | 0.00103136460248285 | 0.000832159268175552 | 0.013118571060022 |
| mammary\_gland\_branching\_involved\_in\_thelarche | 0.00103136460248285 | 0.000832159268175552 | 0.013118571060022 |
| epithelial\_cell\_proliferation\_involved\_in\_salivary\_gland\_morphogenesis | 2.7679213127304e-05 | 1.96374840224565e-05 | 0.013118571060022 |
| cellular\_response\_to\_drug | 0.00103136460248285 | 0.000832159268175552 | 0.013118571060022 |
| branching\_involved\_in\_embryonic\_placenta\_morphogenesis | 0.00103136460248285 | 0.000832159268175552 | 0.013118571060022 |
| astrocyte\_differentiation | 0.00103136460248285 | 0.000832159268175552 | 0.013118571060022 |
| adhesion\_to\_symbiont | 0.00103136460248285 | 0.000832159268175552 | 0.013118571060022 |
| sphingosine\_metabolic\_process | 0.000746240147514607 | 0.000597494840585014 | 0.0116870704924411 |
| skin\_morphogenesis | 0.000746240147514607 | 0.000597494840585014 | 0.0116870704924411 |
| Schwann\_cell\_differentiation | 0.000746240147514607 | 0.000597494840585014 | 0.0116870704924411 |
| response\_to\_ozone | 0.000746240147514607 | 0.000597494840585014 | 0.0116870704924411 |
| regulation\_of\_immunoglobulin\_secretion | 0.000746240147514607 | 0.000597494840585014 | 0.0116870704924411 |
| prostaglandin\_E\_receptor\_activity | 0.000746240147514607 | 0.0290829703132035 | 0.000270125866500665 |
| positive\_regulation\_of\_type\_2\_immune\_response | 0.000746240147514607 | 0.000597494840585014 | 0.0116870704924411 |
| positive\_regulation\_of\_leukocyte\_chemotaxis | 0.000746240147514607 | 0.000597494840585014 | 0.0116870704924411 |
| positive\_regulation\_of\_humoral\_immune\_response\_mediated\_by\_circulating\_immunoglobulin | 0.000746240147514607 | 0.000597494840585014 | 0.0116870704924411 |
| positive\_regulation\_of\_focal\_adhesion\_assembly | 0.000746240147514607 | 0.000597494840585014 | 0.0116870704924411 |
| positive\_regulation\_of\_fibroblast\_growth\_factor\_receptor\_signaling\_pathway | 0.000746240147514607 | 0.000597494840585014 | 0.0116870704924411 |
| positive\_regulation\_of\_cyclin\_dependent\_protein\_kinase\_activity\_involved\_in\_G1\_S | 0.000746240147514607 | 0.000597494840585014 | 0.0116870704924411 |
| positive\_regulation\_of\_corticotropin\_secretion | 0.000746240147514607 | 0.000597494840585014 | 0.0116870704924411 |
| positive\_regulation\_of\_cholesterol\_biosynthetic\_process | 0.000746240147514607 | 0.0290829703132035 | 0.000270125866500665 |
| nuclear\_export | 0.000746240147514607 | 0.000597494840585014 | 0.0116870704924411 |
| nitric\_oxide\_biosynthetic\_process | 0.000746240147514607 | 0.000597494840585014 | 0.0116870704924411 |
| negative\_regulation\_of\_vasoconstriction | 0.000746240147514607 | 0.000597494840585014 | 0.0116870704924411 |
| negative\_regulation\_of\_macrophage\_derived\_foam\_cell\_differentiation | 1.52170396956933e-05 | 0.000597494840585014 | 0.000270125866500665 |
| negative\_regulation\_of\_interleukin\_4\_production | 0.000746240147514607 | 0.000597494840585014 | 0.0116870704924411 |
| negative\_regulation\_of\_epidermal\_growth\_factor\_receptor\_activity | 0.000746240147514607 | 0.000597494840585014 | 0.0116870704924411 |
| monocyte\_chemotaxis | 0.000746240147514607 | 0.000597494840585014 | 0.0116870704924411 |
| metabotropic\_glutamate\_receptor\_signaling\_pathway | 0.000746240147514607 | 0.000597494840585014 | 0.0116870704924411 |
| membrane\_to\_membrane\_docking | 0.000746240147514607 | 0.000597494840585014 | 0.0116870704924411 |
| lung\_lobe\_morphogenesis | 0.000746240147514607 | 0.000597494840585014 | 0.0116870704924411 |
| low\_density\_lipoprotein\_particle\_remodeling | 0.000746240147514607 | 0.0290829703132035 | 0.000270125866500665 |
| kinase\_activator\_activity | 0.000746240147514607 | 0.000597494840585014 | 0.0116870704924411 |
| ISG15\_protein\_conjugation | 0.000746240147514607 | 0.000597494840585014 | 0.0116870704924411 |
| germinal\_center\_formation | 0.000746240147514607 | 0.000597494840585014 | 0.0116870704924411 |
| fever\_generation | 0.000746240147514607 | 0.000597494840585014 | 0.0116870704924411 |
| epithelial\_cell\_differentiation\_involved\_in\_prostate\_gland\_development | 0.000746240147514607 | 0.000597494840585014 | 0.0116870704924411 |
| DNA\_damage\_response\_signal\_transduction\_by\_p53\_class\_mediator\_resulting\_in\_cell\_cycle\_arrest | 0.000746240147514607 | 0.000597494840585014 | 0.0116870704924411 |
| cell\_motility | 0.000746240147514607 | 0.000597494840585014 | 0.0116870704924411 |
| cell\_adhesion\_mediated\_by\_integrin | 0.000746240147514607 | 0.000597494840585014 | 0.0116870704924411 |
| amyloid\_precursor\_protein\_catabolic\_process | 0.000746240147514607 | 0.0290829703132035 | 0.000270125866500665 |
| transformed\_cell\_apoptosis | 0.000520415395319939 | 0.000415532941492481 | 0.0105168255485638 |
| toxin\_transporter\_activity | 0.000520415395319939 | 0.0242306547198831 | 0.000243213399299586 |
| spongiotrophoblast\_differentiation | 6.41619770055183e-06 | 4.52673798906918e-06 | 0.0105168255485638 |
| serine\_type\_carboxypeptidase\_activity | 0.000520415395319939 | 0.000415532941492481 | 0.0105168255485638 |
| RNA\_polymerase\_II\_activating\_transcription\_factor\_binding | 0.000520415395319939 | 0.000415532941492481 | 0.0105168255485638 |
| response\_to\_fungus | 0.000520415395319939 | 0.000415532941492481 | 0.0105168255485638 |
| purinergic\_nucleotide\_receptor\_signaling\_pathway | 0.000520415395319939 | 0.000415532941492481 | 0.0105168255485638 |
| purinergic\_nucleotide\_receptor\_activity | 0.000520415395319939 | 0.000415532941492481 | 0.0105168255485638 |
| positive\_regulation\_of\_tyrosine\_phosphorylation\_of\_Stat1\_protein | 0.000520415395319939 | 0.000415532941492481 | 0.0105168255485638 |
| positive\_regulation\_of\_protein\_transport | 0.000520415395319939 | 0.000415532941492481 | 0.0105168255485638 |
| positive\_regulation\_of\_programmed\_cell\_death | 0.000520415395319939 | 0.000415532941492481 | 0.0105168255485638 |
| positive\_regulation\_of\_interleukin\_6\_biosynthetic\_process | 0.000520415395319939 | 0.000415532941492481 | 0.0105168255485638 |
| positive\_regulation\_of\_hair\_follicle\_development | 0.000520415395319939 | 0.000415532941492481 | 0.0105168255485638 |
| positive\_regulation\_of\_granulocyte\_macrophage\_colony\_stimulating\_factor\_production | 0.000520415395319939 | 0.000415532941492481 | 0.0105168255485638 |
| positive\_regulation\_of\_chemokine\_secretion | 0.000520415395319939 | 0.000415532941492481 | 0.0105168255485638 |
| positive\_regulation\_of\_cell\_migration\_involved\_in\_sprouting\_angiogenesis | 0.000520415395319939 | 0.000415532941492481 | 0.0105168255485638 |
| positive\_regulation\_of\_CD4\_positive\_alpha\_beta\_T\_cell\_differentiation | 0.000520415395319939 | 0.000415532941492481 | 0.0105168255485638 |
| placenta\_blood\_vessel\_development | 0.000520415395319939 | 0.000415532941492481 | 0.0105168255485638 |
| peptide\_catabolic\_process | 0.000520415395319939 | 0.000415532941492481 | 0.0105168255485638 |
| parturition | 0.000520415395319939 | 0.0242306547198831 | 0.000243213399299586 |
| osteoblast\_proliferation | 0.000520415395319939 | 0.000415532941492481 | 0.0105168255485638 |
| nucleotide\_binding\_oligomerization\_domain\_containing\_2\_signaling\_pathway | 0.000520415395319939 | 0.000415532941492481 | 0.0105168255485638 |
| NF\_kappaB\_inducing\_kinase\_activity | 0.000520415395319939 | 0.000415532941492481 | 0.0105168255485638 |
| negative\_regulation\_of\_vasodilation | 0.000520415395319939 | 0.000415532941492481 | 0.0105168255485638 |
| negative\_regulation\_of\_smooth\_muscle\_cell\_differentiation | 0.000520415395319939 | 0.000415532941492481 | 0.0105168255485638 |
| negative\_regulation\_of\_platelet\_activation | 0.000520415395319939 | 0.000415532941492481 | 0.0105168255485638 |
| negative\_regulation\_of\_NF\_kappaB\_import\_into\_nucleus | 0.000520415395319939 | 0.000415532941492481 | 0.0105168255485638 |
| negative\_regulation\_of\_myeloid\_cell\_differentiation | 0.000520415395319939 | 0.000415532941492481 | 0.0105168255485638 |
| negative\_regulation\_of\_leukocyte\_migration | 0.000520415395319939 | 0.000415532941492481 | 0.0105168255485638 |
| negative\_regulation\_of\_calcium\_ion\_transport | 6.41619770055183e-06 | 4.52673798906918e-06 | 0.0105168255485638 |
| necrotic\_cell\_death | 0.000520415395319939 | 0.000415532941492481 | 0.0105168255485638 |
| lung\_epithelium\_development | 0.000520415395319939 | 0.000415532941492481 | 0.0105168255485638 |
| leukemia\_inhibitory\_factor\_signaling\_pathway | 0.000520415395319939 | 0.000415532941492481 | 0.0105168255485638 |
| intracellular\_cholesterol\_transport | 0.000520415395319939 | 0.0242306547198831 | 0.000243213399299586 |
| interleukin\_1\_receptor\_antagonist\_activity | 0.000520415395319939 | 0.000415532941492481 | 0.0105168255485638 |
| epithelial\_cell\_proliferation\_involved\_in\_mammary\_gland\_duct\_elongation | 0.000520415395319939 | 0.000415532941492481 | 0.0105168255485638 |
| cytoplasmic\_sequestering\_of\_NF\_kappaB | 0.000520415395319939 | 0.000415532941492481 | 0.0105168255485638 |
| cellular\_response\_to\_nutrient\_levels | 0.000520415395319939 | 0.000415532941492481 | 0.0105168255485638 |
| cellular\_response\_to\_nicotine | 0.000520415395319939 | 0.000415532941492481 | 0.0105168255485638 |
| CD95\_death\_inducing\_signaling\_complex | 0.000520415395319939 | 0.000415532941492481 | 0.0105168255485638 |
| cardiac\_vascular\_smooth\_muscle\_cell\_differentiation | 0.000520415395319939 | 0.000415532941492481 | 0.0105168255485638 |
| BH3\_domain\_binding | 0.000520415395319939 | 0.000415532941492481 | 0.0105168255485638 |
| activation\_of\_MAPKKK\_activity | 0.000520415395319939 | 0.000415532941492481 | 0.0105168255485638 |
| toll\_like\_receptor\_4\_signaling\_pathway | 0.000314914210087869 | 0.000251200728326204 | 0.00957512134432181 |
| tolerance\_induction | 0.000314914210087869 | 0.000251200728326204 | 0.00957512134432181 |
| thrombospondin\_receptor\_activity | 0.000314914210087869 | 0.000251200728326204 | 0.00957512134432181 |
| tetrahydrobiopterin\_binding | 0.000314914210087869 | 0.000251200728326204 | 0.00957512134432181 |
| superoxide\_generating\_NADPH\_oxidase\_activator\_activity | 0.000314914210087869 | 0.000251200728326204 | 0.00957512134432181 |
| steroid\_hydroxylase\_activity | 0.000314914210087869 | 0.000251200728326204 | 0.00957512134432181 |
| sialic\_acid\_binding | 0.000314914210087869 | 0.000251200728326204 | 0.00957512134432181 |
| sequestering\_of\_triglyceride | 1.84038358487718e-06 | 1.28809090616861e-06 | 0.00957512134432181 |
| rhombomere\_3\_development | 0.000314914210087869 | 0.000251200728326204 | 0.00957512134432181 |
| response\_to\_cycloheximide | 0.000314914210087869 | 0.000251200728326204 | 0.00957512134432181 |
| regulation\_of\_T\_cell\_receptor\_signaling\_pathway | 0.000314914210087869 | 0.000251200728326204 | 0.00957512134432181 |
| regulation\_of\_protein\_import\_into\_nucleus\_translocation | 0.000314914210087869 | 0.000251200728326204 | 0.00957512134432181 |
| regulation\_of\_interleukin\_1\_beta\_production | 0.000314914210087869 | 0.000251200728326204 | 0.00957512134432181 |
| receptor\_biosynthetic\_process | 0.000314914210087869 | 0.000251200728326204 | 0.00957512134432181 |
| positive\_regulation\_of\_transcription\_from\_RNA\_polymerase\_II\_promoter\_during\_mitosis | 0.000314914210087869 | 0.000251200728326204 | 0.00957512134432181 |
| positive\_regulation\_of\_toll\_like\_receptor\_4\_signaling\_pathway | 0.000314914210087869 | 0.000251200728326204 | 0.00957512134432181 |
| positive\_regulation\_of\_protein\_complex\_disassembly | 0.000314914210087869 | 0.000251200728326204 | 0.00957512134432181 |
| positive\_regulation\_of\_podosome\_assembly | 0.000314914210087869 | 0.000251200728326204 | 0.00957512134432181 |
| positive\_regulation\_of\_mesenchymal\_to\_epithelial\_transition\_involved\_in\_metanephros\_morphogenesis | 0.000314914210087869 | 0.000251200728326204 | 0.00957512134432181 |
| positive\_regulation\_of\_killing\_of\_cells\_of\_other\_organism | 0.000314914210087869 | 0.000251200728326204 | 0.00957512134432181 |
| positive\_regulation\_of\_interleukin\_13\_production | 0.000314914210087869 | 0.000251200728326204 | 0.00957512134432181 |
| positive\_regulation\_of\_calcium\_ion\_transport\_via\_store\_operated\_calcium\_channel\_activity | 0.000314914210087869 | 0.000251200728326204 | 0.00957512134432181 |
| positive\_regulation\_of\_calcium\_ion\_import | 0.000314914210087869 | 0.000251200728326204 | 0.00957512134432181 |
| positive\_regulation\_of\_calcidiol\_1\_monooxygenase\_activity | 1.84038358487718e-06 | 1.28809090616861e-06 | 0.00957512134432181 |
| positive\_regulation\_of\_astrocyte\_differentiation | 0.000314914210087869 | 0.000251200728326204 | 0.00957512134432181 |
| phospholipid\_transporter\_activity | 0.000314914210087869 | 0.0186634777017422 | 0.000173795401303742 |
| peptidyl\_cysteine\_S\_nitrosylation | 0.000314914210087869 | 0.000251200728326204 | 0.00957512134432181 |
| oligosaccharide\_binding | 0.000314914210087869 | 0.000251200728326204 | 0.00957512134432181 |
| nuclear\_localization\_sequence\_binding | 0.000314914210087869 | 0.000251200728326204 | 0.00957512134432181 |
| nitric\_oxide\_synthase\_activity | 0.000314914210087869 | 0.000251200728326204 | 0.00957512134432181 |
| negative\_regulation\_of\_synaptic\_transmission\_dopaminergic | 0.000314914210087869 | 0.000251200728326204 | 0.00957512134432181 |
| negative\_regulation\_of\_establishment\_of\_protein\_localization\_in\_plasma\_membrane | 0.000314914210087869 | 0.000251200728326204 | 0.00957512134432181 |
| negative\_regulation\_of\_cytokine\_secretion\_involved\_in\_immune\_response | 0.000314914210087869 | 0.000251200728326204 | 0.00957512134432181 |
| modification\_dependent\_protein\_catabolic\_process | 0.000314914210087869 | 0.000251200728326204 | 0.00957512134432181 |
| mitochondrial\_part | 0.000314914210087869 | 0.000251200728326204 | 0.00957512134432181 |
| leukemia\_inhibitory\_factor\_receptor\_binding | 0.000314914210087869 | 0.000251200728326204 | 0.00957512134432181 |
| induction\_of\_necroptosis\_by\_extracellular\_signals | 0.000314914210087869 | 0.000251200728326204 | 0.00957512134432181 |
| fibrillar\_collagen | 0.000314914210087869 | 0.000251200728326204 | 0.00957512134432181 |
| facial\_nerve\_structural\_organization | 0.000314914210087869 | 0.000251200728326204 | 0.00957512134432181 |
| cytoplasmic\_sequestering\_of\_transcription\_factor | 0.000314914210087869 | 0.000251200728326204 | 0.00957512134432181 |
| chronic\_inflammatory\_response\_to\_antigenic\_stimulus | 1.84038358487718e-06 | 1.28809090616861e-06 | 0.00957512134432181 |
| cellular\_response\_to\_alkaloid | 1.84038358487718e-06 | 1.28809090616861e-06 | 0.00957512134432181 |
| cellular\_process\_regulating\_host\_cell\_cycle\_in\_response\_to\_virus | 0.000314914210087869 | 0.000251200728326204 | 0.00957512134432181 |
| canalicular\_bile\_acid\_transport | 0.000314914210087869 | 0.000251200728326204 | 0.00957512134432181 |
| brain\_segmentation | 0.000314914210087869 | 0.000251200728326204 | 0.00957512134432181 |
| arginine\_catabolic\_process | 0.000314914210087869 | 0.000251200728326204 | 0.00957512134432181 |
| arginine\_binding | 0.000314914210087869 | 0.000251200728326204 | 0.00957512134432181 |
| 1\_phosphatidylinositol\_3\_kinase\_regulator\_activity | 0.000314914210087869 | 0.000251200728326204 | 0.00957512134432181 |
| virion\_attachment\_binding\_of\_host\_cell\_surface\_receptor | 0.00014187411301032 | 0.000112657819014963 | 0.0085735738089241 |
| tyrosine\_phosphorylation\_of\_Stat3\_protein | 0.00014187411301032 | 0.000112657819014963 | 0.0085735738089241 |
| transdifferentiation | 0.00014187411301032 | 0.000112657819014963 | 0.0085735738089241 |
| T\_cell\_activation\_via\_T\_cell\_receptor\_contact\_with\_antigen\_bound\_to\_MHC\_molecule\_on\_antigen\_presenting\_cell | 0.00014187411301032 | 0.000112657819014963 | 0.0085735738089241 |
| substrate\_dependent\_cell\_migration | 0.00014187411301032 | 0.000112657819014963 | 0.0085735738089241 |
| sphinganine\_kinase\_activity | 0.00014187411301032 | 0.000112657819014963 | 0.0085735738089241 |
| rhythmic\_behavior | 0.00014187411301032 | 0.000112657819014963 | 0.0085735738089241 |
| response\_to\_high\_density\_lipoprotein\_particle\_stimulus | 0.00014187411301032 | 0.0129493024363204 | 6.76149354016128e-05 |
| response\_to\_dsRNA | 0.00014187411301032 | 0.000112657819014963 | 0.0085735738089241 |
| response\_to\_auditory\_stimulus | 0.00014187411301032 | 0.000112657819014963 | 0.0085735738089241 |
| regulation\_of\_tumor\_necrosis\_factor\_mediated\_signaling\_pathway | 0.00014187411301032 | 0.000112657819014963 | 0.0085735738089241 |
| regulation\_of\_phosphatidylinositol\_3\_kinase\_activity | 0.00014187411301032 | 0.000112657819014963 | 0.0085735738089241 |
| regulation\_of\_metanephric\_nephron\_tubule\_epithelial\_cell\_differentiation | 0.00014187411301032 | 0.000112657819014963 | 0.0085735738089241 |
| regulation\_of\_keratinocyte\_differentiation | 0.00014187411301032 | 0.000112657819014963 | 0.0085735738089241 |
| regulation\_of\_DNA\_biosynthetic\_process | 0.00014187411301032 | 0.000112657819014963 | 0.0085735738089241 |
| regulation\_of\_cholesterol\_esterification | 0.00014187411301032 | 0.0129493024363204 | 6.76149354016128e-05 |
| protein\_tyrosine\_threonine\_phosphatase\_activity | 0 | 0 | 0.0085735738089241 |
| protein\_tag | 0.00014187411301032 | 0.000112657819014963 | 0.0085735738089241 |
| protein\_disulfide\_reductase\_\_glutathione\_\_activity | 0.00014187411301032 | 0.000112657819014963 | 0.0085735738089241 |
| prostaglandin\_endoperoxide\_synthase\_activity | 0.00014187411301032 | 0.000112657819014963 | 0.0085735738089241 |
| positive\_regulation\_of\_vitamin\_D\_biosynthetic\_process | 0.00014187411301032 | 0.000112657819014963 | 0.0085735738089241 |
| positive\_regulation\_of\_synaptic\_plasticity | 0.00014187411301032 | 0.000112657819014963 | 0.0085735738089241 |
| positive\_regulation\_of\_peptidyl\_serine\_phosphorylation\_of\_STAT\_protein | 0.00014187411301032 | 0.000112657819014963 | 0.0085735738089241 |
| positive\_regulation\_of\_NFAT\_protein\_import\_into\_nucleus | 0.00014187411301032 | 0.000112657819014963 | 0.0085735738089241 |
| positive\_regulation\_of\_monocyte\_chemotactic\_protein\_1\_production | 0.00014187411301032 | 0.000112657819014963 | 0.0085735738089241 |
| positive\_regulation\_of\_macrophage\_differentiation | 0.00014187411301032 | 0.000112657819014963 | 0.0085735738089241 |
| positive\_regulation\_of\_interleukin\_5\_production | 0.00014187411301032 | 0.000112657819014963 | 0.0085735738089241 |
| positive\_regulation\_of\_interleukin\_4\_biosynthetic\_process | 0.00014187411301032 | 0.000112657819014963 | 0.0085735738089241 |
| positive\_regulation\_of\_DNA\_biosynthetic\_process | 0.00014187411301032 | 0.000112657819014963 | 0.0085735738089241 |
| positive\_regulation\_of\_chronic\_inflammatory\_response\_to\_antigenic\_stimulus | 0.00014187411301032 | 0.000112657819014963 | 0.0085735738089241 |
| positive\_regulation\_of\_cellular\_extravasation | 0.00014187411301032 | 0.000112657819014963 | 0.0085735738089241 |
| peptide\_hydrogen\_symporter\_activity | 0.00014187411301032 | 0.000112657819014963 | 0.0085735738089241 |
| PCNA\_p21\_complex | 0.00014187411301032 | 0.000112657819014963 | 0.0085735738089241 |
| nucleotide\_binding\_oligomerization\_domain\_containing\_1\_signaling\_pathway | 0.00014187411301032 | 0.000112657819014963 | 0.0085735738089241 |
| negative\_regulation\_of\_phosphatidylinositol\_biosynthetic\_process | 0.00014187411301032 | 0.000112657819014963 | 0.0085735738089241 |
| negative\_regulation\_of\_meiosis | 0.00014187411301032 | 0.000112657819014963 | 0.0085735738089241 |
| IkappaB\_kinase\_activity | 0.00014187411301032 | 0.000112657819014963 | 0.0085735738089241 |
| histidine\_metabolic\_process | 0.00014187411301032 | 0.000112657819014963 | 0.0085735738089241 |
| glial\_cell\_proliferation | 0.00014187411301032 | 0.000112657819014963 | 0.0085735738089241 |
| follicular\_dendritic\_cell\_differentiation | 0.00014187411301032 | 0.000112657819014963 | 0.0085735738089241 |
| D\_erythro\_sphingosine\_kinase\_activity | 0.00014187411301032 | 0.000112657819014963 | 0.0085735738089241 |
| cyclooxygenase\_pathway | 0.00014187411301032 | 0.000112657819014963 | 0.0085735738089241 |
| complement\_receptor\_mediated\_signaling\_pathway | 0.00014187411301032 | 0.000112657819014963 | 0.0085735738089241 |
| cellular\_response\_to\_mycophenolic\_acid | 0.00014187411301032 | 0.000112657819014963 | 0.0085735738089241 |
| cellular\_extravasation | 0.00014187411301032 | 0.000112657819014963 | 0.0085735738089241 |
| CD40\_signaling\_pathway | 0.00014187411301032 | 0.000112657819014963 | 0.0085735738089241 |
| C5a\_anaphylatoxin\_receptor\_activity | 0.00014187411301032 | 0.000112657819014963 | 0.0085735738089241 |
| Bcl3\_NF\_kappaB2\_complex | 0.00014187411301032 | 0.000112657819014963 | 0.0085735738089241 |
| actin\_filament\_based\_process | 0.00014187411301032 | 0.000112657819014963 | 0.0085735738089241 |
| U\_plasminogen\_activator\_receptor\_activity | 0 | 0 | 0.00666536274069951 |
| tolerance\_induction\_to\_nonself\_antigen | 0 | 0 | 0.00666536274069951 |
| T\_cell\_antigen\_processing\_and\_presentation | 0 | 0 | 0.00666536274069951 |
| sterol\_transporting\_ATPase\_activity | 0 | 0.00714792267412924 | 0 |
| rhombomere\_5\_formation | 0 | 0 | 0.00666536274069951 |
| rhombomere\_3\_formation | 0 | 0 | 0.00666536274069951 |
| response\_to\_sulfur\_dioxide | 0 | 0 | 0.00666536274069951 |
| regulation\_of\_branching\_involved\_in\_salivary\_gland\_morphogenesis | 0 | 0 | 0.00666536274069951 |
| pyrimidine\_base\_transmembrane\_transporter\_activity | 0 | 0 | 0.00666536274069951 |
| purine\_base\_transmembrane\_transporter\_activity | 0 | 0 | 0.00666536274069951 |
| propionate\_catabolic\_process | 0 | 0 | 0.00666536274069951 |
| positive\_regulation\_of\_translational\_initiation\_by\_iron | 0 | 0 | 0.00666536274069951 |
| positive\_regulation\_of\_transforming\_growth\_factor\_beta\_production | 0 | 0 | 0.00666536274069951 |
| positive\_regulation\_of\_toll\_like\_receptor\_3\_signaling\_pathway | 0 | 0 | 0.00666536274069951 |
| positive\_regulation\_of\_platelet\_derived\_growth\_factor\_production | 0 | 0 | 0.00666536274069951 |
| positive\_regulation\_of\_neutrophil\_apoptosis | 0 | 0 | 0.00666536274069951 |
| positive\_regulation\_of\_myosin\_light\_chain\_kinase\_activity | 0 | 0 | 0.00666536274069951 |
| positive\_regulation\_of\_metanephric\_mesenchymal\_cell\_migration | 0 | 0 | 0.00666536274069951 |
| positive\_regulation\_of\_metanephric\_mesenchymal\_cell\_migration\_by\_platelet\_derived\_growth\_factor\_receptor\_beta\_signaling\_pathway | 0 | 0 | 0.00666536274069951 |
| positive\_regulation\_of\_heterotypic\_cell\_cell\_adhesion | 0 | 0 | 0.00666536274069951 |
| positive\_regulation\_of\_glomerular\_mesangial\_cell\_proliferation | 0 | 0 | 0.00666536274069951 |
| positive\_regulation\_of\_glomerular\_filtration | 0 | 0 | 0.00666536274069951 |
| positive\_regulation\_of\_fibroblast\_growth\_factor\_production | 0 | 0 | 0.00666536274069951 |
| positive\_regulation\_of\_calcium\_independent\_cell\_cell\_adhesion | 0 | 0 | 0.00666536274069951 |
| positive\_regulation\_of\_brown\_fat\_cell\_differentiation | 0 | 0 | 0.00666536274069951 |
| positive\_regulation\_of\_adiponectin\_secretion | 0 | 0 | 0.00666536274069951 |
| polyol\_transport | 0 | 0 | 0.00666536274069951 |
| paracrine\_signaling | 0 | 0 | 0.00666536274069951 |
| nicotinic\_acid\_receptor\_activity | 0 | 0 | 0.00666536274069951 |
| negative\_regulation\_of\_toll\_like\_receptor\_3\_signaling\_pathway | 0 | 0 | 0.00666536274069951 |
| negative\_regulation\_of\_T\_helper\_1\_type\_immune\_response | 0 | 0 | 0.00666536274069951 |
| negative\_regulation\_of\_L\_glutamate\_transport | 0 | 0 | 0.00666536274069951 |
| negative\_regulation\_of\_immunoglobulin\_secretion | 0 | 0 | 0.00666536274069951 |
| negative\_regulation\_of\_branching\_involved\_in\_lung\_morphogenesis | 0 | 0 | 0.00666536274069951 |
| metanephric\_glomerular\_mesangial\_cell\_proliferation\_involved\_in\_metanephros\_development | 0 | 0 | 0.00666536274069951 |
| metanephric\_glomerular\_mesangial\_cell\_development | 0 | 0 | 0.00666536274069951 |
| metanephric\_glomerular\_endothelium\_development | 0 | 0 | 0.00666536274069951 |
| interleukin\_1\_secretion | 0 | 0 | 0.00666536274069951 |
| interleukin\_18\_production | 0 | 0 | 0.00666536274069951 |
| histamine\_metabolic\_process | 0 | 0 | 0.00666536274069951 |
| glycoprotein\_transporter\_activity | 0 | 0.00714792267412924 | 0 |
| glycoprotein\_transport | 0 | 0.00714792267412924 | 0 |
| glutathione\_disulfide\_oxidoreductase\_activity | 0 | 0 | 0.00666536274069951 |
| FasL\_biosynthetic\_process | 0 | 0 | 0.00666536274069951 |
| dichotomous\_subdivision\_of\_terminal\_units\_involved\_in\_mammary\_gland\_duct\_morphogenesis | 0 | 0 | 0.00666536274069951 |
| complement\_component\_C3a\_binding | 0 | 0 | 0.00666536274069951 |
| cholesterol\_25\_hydroxylase\_activity | 0 | 0 | 0.00666536274069951 |
| cellular\_response\_to\_interleukin\_2 | 0 | 0 | 0.00666536274069951 |
| C3a\_anaphylatoxin\_receptor\_activity | 0 | 0 | 0.00666536274069951 |
| B\_1\_B\_cell\_differentiation | 0 | 0 | 0.00666536274069951 |
| amine\_transport | 0 | 0 | 0.00666536274069951 |
| 2\_methylcitrate\_dehydratase\_activity | 0 | 0 | 0.00666536274069951 |
| 15\_hydroxyprostaglandin\_dehydrogenase\_\_NAD\_\_\_activity | 0 | 0.00714792267412924 | 0 |
