# Supplementary material for: Inferring active regulatory networks from gene expression data using a combination of prior knowledge and enrichment analysis
Source: BMC Bioinformatics. 2016 Jun 6;17(Suppl 5):181. doi: 10.1186/s12859-016-1040-7 (PMC4905609; doi:10.1186/s12859-016-1040-7)
Supplement: Additional file 2: — Mouse Test case results. Additional file 2 is a folder containing the detailed results of the Mouse Test case in HTML format. Each file includes the respective calculated enrichments for TFs, miRNAs, KEGG pathways, KEGG pathway categories and GO terms. In order to view the results a standard web-browser is needed (Chrome and Mozilla Firefox have been tested). The HTML files must be opened from inside the folder because additional files (images and javascripts) which are needed for the correct view of the results are included. (ZIP 83 kb) [file 12859_2016_1040_MOESM2_ESM.zip › AdditionalFiles2/GSE63889 KEGG_Enrichment.html]

GSE63889 KEGG\_Enrichment


| KEGG\_pathway | DE\_qvalue | UP\_qvalue | DOWN\_qvalue |
| --- | --- | --- | --- |
| PI3K-Akt\_signaling\_pathway | 0.0192082256242855 | 0.0107760076700692 | 0.220969404551054 |
| Pathways\_in\_cancer | 4.31931168408889e-05 | 1.53304102515094e-05 | 0.206920580896907 |
| Neuroactive\_ligand-receptor\_interaction | 0.197906009452957 | 0.153491892797805 | 0.184625092627552 |
| HTLV-I\_infection | 3.30782663236756e-06 | 8.60088474282627e-07 | 0.184414391295085 |
| MicroRNAs\_in\_cancer | 0.000297862077743698 | 0.000122518665196463 | 0.179320760471347 |
| Cytokine-cytokine\_receptor\_interaction | 1.82308562056016e-06 | 4.61779588033582e-07 | 0.175948952962731 |
| MAPK\_signaling\_pathway | 1.80234906902083e-06 | 4.55698684524259e-07 | 0.175628415712424 |
| Viral\_carcinogenesis | 0.000124153457800366 | 4.92442659923301e-05 | 0.158042191354586 |
| Proteoglycans\_in\_cancer | 0.00055892001960025 | 0.000267573022743882 | 0.158042191354586 |
| Endocytosis | 0.326480362504055 | 0.273053065428392 | 0.155648579448428 |
| Regulation\_of\_actin\_cytoskeleton | 0.041760888001309 | 0.0285733913148372 | 0.155648579448428 |
| Epstein-Barr\_virus\_infection | 7.67172511377199e-05 | 3.00609723381388e-05 | 0.152461356046416 |
| Focal\_adhesion | 0.0376583381535223 | 0.0250744326000783 | 0.151151570309088 |
| Herpes\_simplex\_infection | 8.75970877346454e-06 | 3.17557084782348e-06 | 0.150502341097403 |
| Chemokine\_signaling\_pathway | 4.31931168408889e-05 | 1.74823621328126e-05 | 0.144922142215842 |
| Calcium\_signaling\_pathway | 0.0903944335722541 | 0.0671998479331747 | 0.140616701867457 |
| Transcriptional\_misregulation\_in\_cancer | 2.93679227182776e-05 | 8.01944147929016e-05 | 0.109537888928335 |
| Tuberculosis | 0.000171165546279067 | 7.9857406036262e-05 | 0.137646021747607 |
| Phagosome | 0.241800565030124 | 0.198987984890467 | 0.136066808873912 |
| Alzheimers\_disease | 0.082626527627214 | 0.0613496984978663 | 0.136066808873912 |
| Purine\_metabolism | 0.237857562369946 | 0.197207318153398 | 0.136066808873912 |
| Influenza\_A | 2.87421930730142e-10 | 5.13516205949287e-11 | 0.136066808873912 |
| Protein\_processing\_in\_endoplasmic\_reticulum | 0.233856183774804 | 0.193684222002211 | 0.136066808873912 |
| Cell\_adhesion\_molecules\_(CAMs) | 0.000535057068506663 | 0.000270482771716607 | 0.129900038580244 |
| Hippo\_signaling\_pathway | 0.0654224485267351 | 0.0476757374811891 | 0.129676416879463 |
| Jak-STAT\_signaling\_pathway | 0.000486736040984101 | 0.000249522411940741 | 0.12944596246942 |
| Systemic\_lupus\_erythematosus | 0.193289385747628 | 0.157372767886894 | 0.125162548675563 |
| Hepatitis\_B | 7.85105492613723e-06 | 3.17557084782348e-06 | 0.125162548675563 |
| Insulin\_signaling\_pathway | 0.0539912208421464 | 0.0395590415161499 | 0.124566458175726 |
| Ubiquitin\_mediated\_proteolysis | 0.180931891623361 | 0.148222313622001 | 0.123414351669152 |
| Measles | 4.31931168408889e-05 | 1.8649689991773e-05 | 0.123083471503302 |
| Hepatitis\_C | 5.89453274007723e-06 | 2.13020186946426e-06 | 0.122742862169863 |
| Serotonergic\_synapse | 0.0441201337443511 | 0.0321588167712368 | 0.118018312954704 |
| Osteoclast\_differentiation | 5.38565580917727e-07 | 1.95760236865523e-07 | 0.117601058413227 |
| Cell\_cycle | 0.0425497545985797 | 0.0306149466716076 | 0.117601058413227 |
| Neurotrophin\_signaling\_pathway | 0.0409880038940056 | 0.0294585240081223 | 0.116729838870633 |
| Natural\_killer\_cell\_mediated\_cytotoxicity | 0.00828343632713525 | 0.00529466080790893 | 0.116729838870633 |
| Leukocyte\_transendothelial\_migration | 0.0398953443375684 | 0.0285733913148372 | 0.116729838870633 |
| Amoebiasis | 0.000158769308476425 | 7.9857406036262e-05 | 0.116729838870633 |
| Glutamatergic\_synapse | 0.135505796809147 | 0.109968593372597 | 0.115788324192398 |
| Toxoplasmosis | 0.000124153457800366 | 6.25804206927341e-05 | 0.114316081945268 |
| Cholinergic\_synapse | 0.131021440779152 | 0.106210187426788 | 0.114316081945268 |
| HIF-1\_signaling\_pathway | 0.000806124242762077 | 0.000444778736714509 | 0.114316081945268 |
| TNF\_signaling\_pathway | 9.63306976347002e-22 | 6.5870083616995e-23 | 0.114316081945268 |
| T\_cell\_receptor\_signaling\_pathway | 0.000100910952781516 | 4.91797893314993e-05 | 0.114167773692194 |
| Retrograde\_endocannabinoid\_signaling | 0.113488964337242 | 0.0916350202876522 | 0.111511380569891 |
| Chagas\_disease\_(American\_trypanosomiasis) | 1.26253005539022e-06 | 4.55698684524259e-07 | 0.111511380569891 |
| Toll-like\_receptor\_signaling\_pathway | 7.85105492613723e-06 | 3.17557084782348e-06 | 0.111511380569891 |
| NF-kappa\_B\_signaling\_pathway | 7.21286183517916e-18 | 7.83125798803285e-19 | 0.111511380569891 |
| Estrogen\_signaling\_pathway | 0.105384203232976 | 0.08492541029273 | 0.111511380569891 |
| Arachidonic\_acid\_metabolism | 0.104540479030165 | 0.084218383375957 | 0.111511380569891 |
| Chemical\_carcinogenesis | 0.101872532717013 | 0.0820092865172167 | 0.111511380569891 |
| Morphine\_addiction | 0.0973876787141996 | 0.0783097273951798 | 0.110252971189741 |
| Dilated\_cardiomyopathy | 0.0205527509027451 | 0.0143654261148053 | 0.109537888928335 |
| Viral\_myocarditis | 0.0929097481863107 | 0.0746239071451591 | 0.109537888928335 |
| Prostate\_cancer | 0.000335504500928664 | 0.000183584048072047 | 0.109537888928335 |
| Fc\_gamma\_R-mediated\_phagocytosis | 0.000335504500928664 | 0.000183584048072047 | 0.109537888928335 |
| Gap\_junction | 0.0920054531704503 | 0.0738740588196861 | 0.109537888928335 |
| Retinol\_metabolism | 0.0910947205185179 | 0.0731196170013699 | 0.109537888928335 |
| Progesterone-mediated\_oocyte\_maturation | 0.0910947205185179 | 0.0731196170013699 | 0.109537888928335 |
| Hematopoietic\_cell\_lineage | 0.00269019161149137 | 0.00164313049613612 | 0.109537888928335 |
| ErbB\_signaling\_pathway | 0.00269019161149137 | 0.00164313049613612 | 0.109537888928335 |
| ECM-receptor\_interaction | 0.0192082256242855 | 0.0134662027701772 | 0.109537888928335 |
| Small\_cell\_lung\_cancer | 3.31279455111266e-06 | 1.33772575956698e-06 | 0.109537888928335 |
| Hypertrophic\_cardiomyopathy\_(HCM) | 0.017999862713516 | 0.0124051565812788 | 0.109537888928335 |
| Apoptosis | 3.30782663236756e-06 | 1.33494918814412e-06 | 0.109537888928335 |
| Rheumatoid\_arthritis | 0.000259739618349976 | 0.000138207658181943 | 0.109537888928335 |
| TGF-beta\_signaling\_pathway | 0.0847149945490375 | 0.0671998479331747 | 0.109537888928335 |
| Phosphatidylinositol\_signaling\_system | 0.08376699830841 | 0.0670981349829848 | 0.109537888928335 |
| Peroxisome | 0.0828126214054109 | 0.0663131195452721 | 0.109537888928335 |
| Salmonella\_infection | 0.000210613754045197 | 0.000114929876228503 | 0.109537888928335 |
| Antigen\_processing\_and\_presentation | 0.0808847006996455 | 0.064055273316944 | 0.109537888928335 |
| Complement\_and\_coagulation\_cascades | 0.000191384429618273 | 0.000104403310836701 | 0.109537888928335 |
| Bacterial\_invasion\_of\_epithelial\_cells | 0.0144557768887307 | 0.0101933653408945 | 0.109537888928335 |
| B\_cell\_receptor\_signaling\_pathway | 0.0016843564192152 | 0.00102292555489902 | 0.109537888928335 |
| Prolactin\_signaling\_pathway | 0.0133577621675778 | 0.00926515145382298 | 0.109537888928335 |
| Pertussis | 1.59140670321051e-05 | 7.34125829638935e-06 | 0.109537888928335 |
| Arrhythmogenic\_right\_ventricular\_cardiomyopathy\_(ARVC) | 0.0745220333021727 | 0.0595452079863424 | 0.109537888928335 |
| Chronic\_myeloid\_leukemia | 0.000158769308476425 | 8.29227172757756e-05 | 0.109537888928335 |
| Bile\_secretion | 0.071758174133214 | 0.0572955111866716 | 0.109537888928335 |
| Melanoma | 0.00132754067097999 | 0.00080413350612009 | 0.109537888928335 |
| Fc\_epsilon\_RI\_signaling\_pathway | 0.0121003679302189 | 0.00838088615400053 | 0.109537888928335 |
| Adipocytokine\_signaling\_pathway | 0.000145544443546142 | 7.9857406036262e-05 | 0.109537888928335 |
| RIG-I-like\_receptor\_signaling\_pathway | 1.26253005539022e-06 | 4.55698684524259e-07 | 0.109537888928335 |
| p53\_signaling\_pathway | 0.000124153457800366 | 0.000694891298817105 | 0.0615814196016013 |
| Renal\_cell\_carcinoma | 0.0104629595458228 | 0.00723282223230343 | 0.109537888928335 |
| Pancreatic\_cancer | 0.0101948794777292 | 0.00704410045350507 | 0.109537888928335 |
| Leishmaniasis | 8.75970877346454e-06 | 3.89666694279118e-06 | 0.109537888928335 |
| Glioma | 0.000967036476832773 | 0.000595852853816475 | 0.109537888928335 |
| Colorectal\_cancer | 0.0594272983120531 | 0.0473022683067657 | 0.109537888928335 |
| VEGF\_signaling\_pathway | 0.000823116707761357 | 0.000506592174262284 | 0.109537888928335 |
| mTOR\_signaling\_pathway | 0.00869716408065242 | 0.00599764602228232 | 0.109537888928335 |
| Cytosolic\_DNA-sensing\_pathway | 6.39855459960954e-06 | 2.92844877370801e-06 | 0.109537888928335 |
| Type\_I\_diabetes\_mellitus | 0.00781925811671322 | 0.00529466080790893 | 0.109537888928335 |
| Inflammatory\_bowel\_disease\_(IBD) | 0.00781925811671322 | 0.00529466080790893 | 0.109537888928335 |
| Ovarian\_steroidogenesis | 0.050842246978311 | 0.0399278905183522 | 0.109537888928335 |
| NOD-like\_receptor\_signaling\_pathway | 2.37472560622117e-08 | 8.19784499862559e-09 | 0.109537888928335 |
| Legionellosis | 5.38565580917727e-07 | 1.95760236865523e-07 | 0.109537888928335 |
| Acute\_myeloid\_leukemia | 0.00734120671334612 | 0.00514049203172994 | 0.109537888928335 |
| Arginine\_and\_proline\_metabolism | 0.0482281581403406 | 0.0382729883657622 | 0.109537888928335 |
| Non-small\_cell\_lung\_cancer | 0.045635739002144 | 0.0361896978124656 | 0.109537888928335 |
| Graft-versus-host\_disease | 0.00652470893633065 | 0.00448206982205949 | 0.109537888928335 |
| Amyotrophic\_lateral\_sclerosis\_(ALS) | 0.000525084736594205 | 0.000308396685192545 | 0.109537888928335 |
| Allograft\_rejection | 0.0446034184028965 | 0.0353607279466023 | 0.109537888928335 |
| Staphylococcus\_aureus\_infection | 0.000474284617990736 | 0.000271810193159928 | 0.109537888928335 |
| Endometrial\_cancer | 0.0440812070230973 | 0.0345303809716444 | 0.109537888928335 |
| Type\_II\_diabetes\_mellitus | 0.000416479813038949 | 0.000249522411940741 | 0.109537888928335 |
| Sphingolipid\_metabolism | 0.0398953443375684 | 0.0306149466716076 | 0.109537888928335 |
| Malaria | 2.93679227182776e-05 | 1.53304102515094e-05 | 0.109537888928335 |
| ABC\_transporters | 0.0377920013259683 | 0.197394497115668 | 0.0564672187873021 |
| Carbohydrate\_digestion\_and\_absorption | 0.0372157104995066 | 0.0285733913148372 | 0.109537888928335 |
| Intestinal\_immune\_network\_for\_IgA\_production | 0.0346296701874594 | 0.0266718210770964 | 0.109537888928335 |
| Aldosterone-regulated\_sodium\_reabsorption | 0.0306267471910075 | 0.0238684362159574 | 0.109537888928335 |
| Bladder\_cancer | 0.0281701978527406 | 0.0219351172464369 | 0.109537888928335 |
| Prion\_diseases | 0.0244209931812792 | 0.0189912107437842 | 0.109537888928335 |
| African\_trypanosomiasis | 7.85105492613723e-06 | 3.69220885754168e-06 | 0.109537888928335 |
| Histidine\_metabolism | 0.017999862713516 | 0.0135682408418205 | 0.109537888928335 |
| Asthma | 0.01409930174038 | 0.0107541657861632 | 0.109537888928335 |
| Primary\_bile\_acid\_biosynthesis | 0.006858252490295 | 0.00519675054627026 | 0.109537888928335 |
