# Supplementary material for: Inferring active regulatory networks from gene expression data using a combination of prior knowledge and enrichment analysis
Source: BMC Bioinformatics. 2016 Jun 6;17(Suppl 5):181. doi: 10.1186/s12859-016-1040-7 (PMC4905609; doi:10.1186/s12859-016-1040-7)
Supplement: Additional file 2: — Mouse Test case results. Additional file 2 is a folder containing the detailed results of the Mouse Test case in HTML format. Each file includes the respective calculated enrichments for TFs, miRNAs, KEGG pathways, KEGG pathway categories and GO terms. In order to view the results a standard web-browser is needed (Chrome and Mozilla Firefox have been tested). The HTML files must be opened from inside the folder because additional files (images and javascripts) which are needed for the correct view of the results are included. (ZIP 83 kb) [file 12859_2016_1040_MOESM2_ESM.zip › AdditionalFiles2/GSE63889 miRNA_Enrichment.html]

GSE63889 miRNA\_Enrichment


| miRNA | DE\_qvalue | UP\_qvalue | DOWN\_qvalue |
| --- | --- | --- | --- |
| mmu-let-7b-5p | 0.0788466908689693 | 0.335321596988883 | 0.00674580652671381 |
| mmu-miR-9-5p | 0.0788466908689693 | 0.0769239427186774 | 0.057006793830604 |
| mmu-miR-15a-5p | 0.0788466908689693 | 0.0769239427186774 | 0.057006793830604 |
| mmu-miR-17-5p | 0.0772102870361533 | 0.0601725282755229 | 0.0557297099854217 |
